# An array CGH based genomic instability index (G2I) is predictive of clinical outcome in breast cancer and reveals a subset of tumors without lymph node involvement but with poor prognosis

**DOI:** 10.1186/1755-8794-5-54

**Published:** 2012-11-27

**Authors:** Françoise Bonnet, Mickael Guedj, Natalie Jones, Sana Sfar, Véronique Brouste, Nabila Elarouci, Guillaume Banneau, Béatrice Orsetti, Charlotte Primois, Christine Tunon de Lara, Marc Debled, Isabelle de Mascarel, Charles Theillet, Nicolas Sévenet, Aurélien de Reynies, Gaëtan MacGrogan, Michel Longy

**Affiliations:** 1 Inserm U 916 Institut Bergonié, Université de Bordeaux, Bordeaux, France; 2 Cancer Genetics Unit, Institut Bergonié, Bordeaux, France; 3 «Cartes d’Identité des Tumeurs »(CIT) program, Ligue Nationale Contre le Cancer, Paris, France; 4 Clinical and Epidemiological Research Unit, Institut Bergonié, Bordeaux, France; 5 Inserm U 896, IRCM – Centre Val d’Aurelle – Paul Lamarque, Montpellier, France; 6 Surgery Department, Institut Bergonié, Bordeaux, France; 7 Medical Oncology Department, Institut Bergonié, Bordeaux, France; 8 Pathology Department, Institut Bergonié, Bordeaux, France; 9 Inserm U916, Institut Bergonié, 229 Cours de l’Argonne, Bordeaux cedex, 33076, France

**Keywords:** Breast cancer, Array CGH, Prognosis, Genetic instability

## Abstract

**Background:**

Despite entering complete remission after primary treatment, a substantial proportion of patients with early stage breast cancer will develop metastases. Prediction of such an outcome remains challenging despite the clinical use of several prognostic parameters. Several reports indicate that genomic instability, as reflected in specific chromosomal aneuploidies and variations in DNA content, influences clinical outcome but no precise definition of this parameter has yet been clearly established.

**Methods:**

To explore the prognostic value of genomic alterations present in primary tumors, we performed a comparative genomic hybridization study on BAC arrays with a panel of breast carcinomas from 45 patients with metastatic relapse and 95 others, matched for age and axillary node involvement, without any recurrence after at least 11 years of follow-up. Array-CGH data was used to establish a two-parameter index representative of the global level of aneusomy by chromosomal arm, and of the number of breakpoints throughout the genome.

**Results:**

Application of appropriate thresholds allowed us to distinguish three classes of tumors highly associated with metastatic relapse. This index used with the same thresholds on a published set of tumors confirms its prognostic significance with a hazard ratio of 3.24 [95CI: 1.76-5.96] p = 6.7x10^-5^ for the bad prognostic group with respect to the intermediate group. The high prognostic value of this genomic index is related to its ability to individualize a specific group of breast cancers, mainly luminal type and axillary node negative, showing very high genetic instability and poor outcome. Indirect transcriptomic validation was obtained on independent data sets.

**Conclusion:**

Accurate evaluation of genetic instability in breast cancers by a genomic instability index (G2I) helps individualizing specific tumors with previously unexpected very poor prognosis.

## Background

Despite entering complete remission after primary treatment, a substantial proportion of patients with early stage breast cancers will evolve towards metastatic relapse, sometimes after a delay of many years [1]. Such an evolution led to the concept of cell dormancy in which the metastatic process results from the migration of individual cells capable of forming a new tumoral localization, even after a long latency [2]. This model, suggesting heterogeneity of the metastatic power within the constitutive cells of a primary tumor, found a new interest with the hypothesis of the existence of cancer stem cells capable to generate such secondary localizations [3].

Eliminating these cells is the objective of adjuvant therapy which is given after optimal local treatment. Efficiency of such a therapeutic strategy is well established [4], but accurate identification of patients for adjuvant treatment requires appropriate prognostic factors that are not clearly established. The main conventional prognostic factor in early breast carcinoma is the staging of axillary node involvement reflecting the cancer cells’ ability to diffuse and the level of invasion [5]. This criterion however is not completely accurate in predicting patient outcome since 25% of patients without axillary lymph node invasion show metastatic relapse at ten years [6]. Among the many other factors that have been tested, several show proven prognostic value such as tumor size, histological grade [7], peritumoral vascular emboli, or the expression of steroid hormone receptors [8]. With the advent of gene expression profiling and the identification of five intrinsic breast cancer subtypes [9-11], prognosis in breast cancer is now considered within each molecular subtype. Subsequent gene expression studies have identified prognostic transcriptomic profiles that appear to be pertinent for the prognostication of short term relapses, specifically in estrogen receptor positive breast cancer [12,13].

The ability of gene signatures from bulk tumors to predict metastatic relapse is difficult to reconcile with the model putting forward that rare tumor stem cells mediate metastasis [14]. It is necessary to conceive that the various described prognostic signatures are the reflection of an intrinsic characteristic of cancer cells rather than a specific biological characteristic including the ability to migrate and to form cell colonies outside of the primary site [15]. Effectively, most of the proposed prognostic signatures reflect an increased expression of proliferation genes, one of the hallmarks of cancer [16]. Because another hallmark of cancer is loss of genetic stability and because gene expression signatures linked to chromosomal instability have shown some predictive value for metastatic relapse in various kinds of cancer [17,18], we explore by array-CGH analysis the prognostic value of genomic alterations in a series of breast carcinomas with known outcomes after 11 years of follow-up and confirm the main results obtained on publicly available sets of tumors.

## Methods

### Patient samples

Tumor samples are from the tumor bank of “*Institut Bergonié*” and come from 135 patients diagnosed with invasive ductal carcinoma with surgical resection as first treatment performed between 1989 and 1992. The study was performed in accordance with *Institut Bergonié*’s clinical research committee rules. All patients consented to the use of their samples for research purposes, in compliance with the French law on tumor banks (law n° 2004–800).

Forty-five tumor samples with metastatic relapse and ninety samples without metastatic relapse were selected, with a minimum follow-up of 131 months (11 years). From each group, tumors were matched for patient age at diagnosis (< or > to 55 years) and for axillary lymph node involvement (Table 1). Mean patient age across both groups was 55 years (range: 29 years-77 years).

**Table 1:** Description of pair-wise groups

	**All n = 135 (%)**		**pN0 n = 75**		**pN + n = 60**	
	**M-**	**M+**	**M-**	**M+**	**M-**	**M+**
Age < =55 n= 70	47 (35)	23 (17)	26 (35)	13 (17)	21 (35)	10 (17)
Age > 55 n = 65	43 (32)	22 (16)	24 (32)	12 (16)	19 (32)	10 (17)
All n = 135	90 (67)	45 (33)	50 (67)	25 (33)	40 (67)	20 (34)

n: Number of tumors; Age in years; pN0/pN+: absence/presence of lymph node invasion; M- / M+: absence of detection/detection of distant metastasis at 11 years.

Clinicopathological characteristics of tumors are given in Additional file 1. Patients with tumors without axillary lymph node involvement only received local treatment (lumpectomy and radiotherapy, or mastectomy with or without radiotherapy), whereas patients with tumors with lymph node involvement received adjuvant therapy, either chemotherapy or hormone therapy, according to the procedures used at the time.

### Array CGH

Sample preparation

A fragment of tumoral tissue was immediately snap frozen in liquid nitrogen after surgical removal and stored at −140°C in the tumor bank of “*Institut Bergonié*”. After grinding in liquid nitrogen, DNA was purified according to a standard methodology based on organic solvents.

Micro-array hybridization

Array-CGH was performed on human Integrachip V7 slides (Integragen SA, Evry, France, http://www.integragen.com). IntegraChip V7 is composed of 5878 BAC clones with a median of 0.5 Mb between clones. BAC clones are spotted in quadruplicate. Hybridizations were performed according to the manufacturer’s recommendations (see Additional file 2).

Data analysis

The CAPweb (Copy number Array analysis Platform on the web) developed by Institut Curie (CAPWeb, http://bioinfo-out.curie.fr/CAPweb/) was used for normalization (MANOR package), segmentation and smoothing (GLAD package) as detailed in Additional file 2. Graphical representation of genomic alterations was performed with VAMP software (http://bioinfo.curie.fr/vamp)[19]. Gains and losses were defined as values of Cy3 to Cy5 smoothed log2 ratio more than the standard deviation between normalized and smoothed log 2 ratio for all the autosomes (see Additional file 2 for details). The array-CGH data are available in the ArrayExpress database (http://www.ebi.ac.uk/arrayexpress/ accession number: E-MTAB-748).

### Expression profiling

Rneasy Mini Kits (Qiagen, Courtaboeuf, France) were used to extract total RNA from samples, ground to powder while frozen. RNA quality was assessed using the Agilent 2100 Bioanalyzer (Agilent Technologies). Gene-expression analyses were performed by the IGBMC and Génopole Alsace-Lorraine Affymetrix service using Affymetrix U133 Plus 2.0 genechip microarrays as detailed in Additional file 2. The transcriptomic data are available in ArrayExpress database (http://www.ebi.ac.uk/arrayexpress/ accession number: E-MTAB-748).

### Tissue micro-array and immunohistochemistry

Corresponding Holland Bouin-fixed paraffin-embedded tumor blocks were retrieved from the hospital files and were used to construct a tissue microarray (TMA) comprising four representative 0.6 mm cores for every tumor. The TMA was made with an Alphelys tissue arrayer. Immunohistochemical analysis was performed on a Dako autostainer as described in Additional file 2. An immunohistochemical transposition of the transcriptomic intrinsinc molecular classification of breast cancer was performed according to Nielsen et al. [20]. Luminal type A tumors were ER or PR positive (≥ 10% positive tumor cells) with a Mib1 proliferation index <20% and Her2 scores 0, 1+ or 2+. Luminal type B tumors were ER or PR positive with a Mib1 proliferation index ≥ 20% or a Her2 score 3+. Her2-enriched tumors were ER and PR negative and Her2 score 3+. Finally Basal-like tumors were ER and PR negative, Her2 0, 1+ or 2+ and CK5/6 or EGFR positive.

### TP53 mutation analysis

TP53 coding exons (2–11) were amplified as 7 amplicons (exons 2 and 3, 5 and 6, 8 and 9 respectively in a same amplicon) which were screened for point mutations through a combination of dHPLC followed by sequencing of variants (exons 4–11) or sequencing directly (exons 2–3) on a 3130XL ABI DNA sequencing machine. Primer sequences and PCR conditions are available on request.

### Statistical considerations

#### Clustering of genome copy number profile

Samples were clustered based on “gain, normal, loss” (GNL) data, using an Agglomerative Hierarchical Clustering (described in Additional file 2). The number of groups (n = 6) was assessed qualitatively by considering the shape of the clustering dendrogram and the homogeneity of the chromosomal rearrangements within each cluster.

#### Genomic instability index (G2I)

The proposed score is based on two items: (i) the overall level of genomic alteration (noted A) and (ii) the number of altered genomic regions (noted N). By applying a set of appropriate thresholds on these two items, we can define three groups with genomic scores 1, 2 and 3, characterized by an increasing level of genomic perturbation. For a given sample i, let N_i_ and A_i_ be respectively the computed values N and A. Let a_1_, a_2_, n_1,_ and n_2_ be the thresholds:

If A_i_ < a_1_ and N_i_ < n_1_ then genomic score = 1 (low level of perturbation)

If A_i_ > a_2_ and N_i_ > n_2_ then genomic score = 3 (high level of perturbation)

Else genomic score = 2 (average level of perturbation)

The calculation of A and N as well as the estimation of the thresholds a_1_, a_2_, n_1,_ and n_1_ are described in Additional file 2. The R script that allows reproducing the results is provided in supplemental data (Additional file 3).

#### Predictive analysis

A univariate logistic regression model was used to define the odd ratios between the G2I classes and metastatic relapse as well as for the classical prognostic parameters. Factors significant at p < 0.05 in univariate analysis were included in a maximum likelihood logistic regression model in ascending order.

#### Validation

An external validation using publicly available BAC arrays CGH data from 168 invasive ductal carcinomas of the breast [21] was performed. This set of tumors, including 57 cases with metastatic relapse and 111 tumors without metastatic or loco regional recurrence after a follow-up of at least 5 years (median follow-up: 130 months; range: 71–210), consists only of node negative breast cancers. Array-CGH data are from 6 distinct BAC arrays but similar to this one used in the present study. Application of the G2I to this set of tumors using the previously defined thresholds is described in Additional file 2.

#### Transcriptomic signature of the G2I-3 tumors

To identify genes differentially expressed between G2I-1/2 and G2I-3 tumors, based on the RMA log2 single-intensity expression data, we used Welch’s T-tests (t-test function, R package stats) with a threshold of 5x10^-3^ on p values leading to 300 probe sets (associated to 222 unique EntrezGene symbols). Then, samples were clustered based on this signature using an Agglomerative hierarchical clustering.

#### Comparison of four prognostic molecular signatures in three independent datasets

The molecular signature deduced from the genomic instability index (G2I) was compared to three well-known prognostic signatures: Amsterdam [22], GGI of Sotiriou [23] and the intrinsic gene sets used by Sorlie et al. to identify their five molecular subtypes [11]. The four signatures were applied to independent datasets according to an approach inspired from Fan et al. [24] and described in Additional file 2. This comparison is done in three independent datasets corresponding to i) this study, ii) the Rotterdam study [25] and iii) the Loi study [26].

## Results

### Unsupervised clustering of array-CGH data identifies six groups of tumors

**Figure 1:**
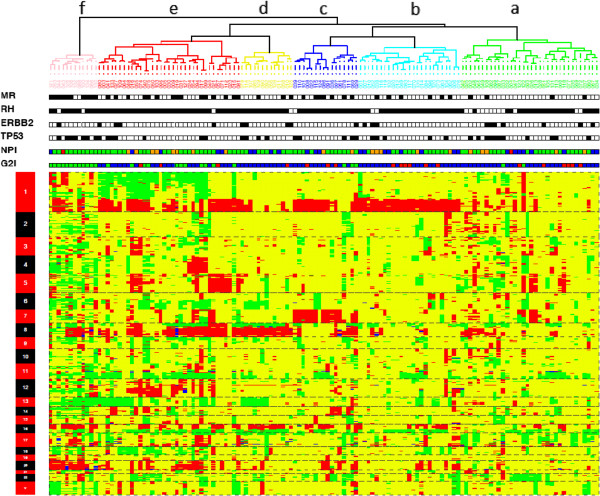
**tumoral classification according to array CGH genomic profile.** Unsupervised hierarchical clustering using genomic copy number variation enables the classification of the 135 breast carcinomas into six branches. MR, black: metastatic relapse; HR, black: positive for steroid hormonal receptors; Her2, black: Her2 score 3+; p53, black: TP53 mutation or protein overexpression; NPI (Nottingham prognostic index) red: NPI-1, blue: NPI-2, green: NPI-3, orange: NPI-4; G2I (genomic instability index) red: G2I-1, blue: G2I-2, green: G2I-3.

**Figure 2:**
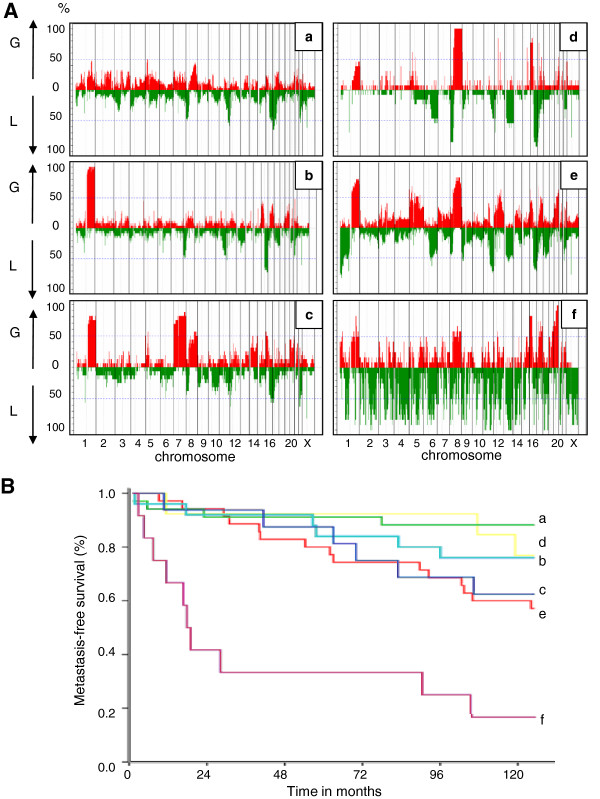
**genomic characteristics and prognostic value of each array CGH cluster.** (**A**): Frequency plot of copy number variation showing the dominant genomic changes in each array CGH cluster. (**B**): Kaplan Meier curves showing the poor outcome of cluster “f” tumors according to the other array CGH groups.

To identify broad patterns of large scale genomic rearrangement, we performed unsupervised clustering based on the “gain, normal, loss” (GNL) profile of each tumor (Figure 1). The clustering of the tumors into six main groups was driven mainly by gains or losses of whole chromosomal arms, particularly on chromosomes 1, 7, 8, 11, 16, 17 and 20. The dominant changes in each group are more readily seen in whole genome plots showing the cumulative changes at each locus (Figure 2A). The groups are labeled according to the clusters in Figure 1, which are described below.

“*Cluster a*” comprises tumors without recurrent changes affecting any particular chromosome. The only copy number change seen in more than 60% of cases was loss of 17p13 (Additional file 1 and Figure 2A-a). Copy number variations involving small genomic regions can be observed, sometimes frequently in a same tumor, but without recurrence of a specific change from one tumor to another.

“*Cluster b*” comprises tumors with gains of the long arm of chromosome 1 and losses of the long arm of chromosome 16, a common rearrangement frequently linked to the well-known unbalanced translocation t(1;16). The only other rearrangements were losses of 8p and 11q observed in nearly 50% of the tumors (Additional file 1 and Figure 2A-b).

“*Cluster c*” comprises tumors with two chromosomal rearrangements, gain of 1q and gain of the entire chromosome 7 which were present in 80% of the tumors in this cluster. To our knowledge, the specific association of both of these chromosome rearrangements was not previously noted in breast cancer. Other common changes were loss of 12p13 and gain of 8q (Additional file 1 and Figure 2A-c).

“*Cluster d*” comprises tumors characterized by the association of rearrangements of chromosomes 8 and 16 with loss of the entire 8p and 16q arms and gain of the 8q and 16p arms in nearly 100% of cases. Other chromosomal rearrangements are less frequently associated, such as loss of 6q, loss of 13q and loss of 17p. Interestingly, some specific rearrangements affecting small regions are observed with high frequency in this specific group of tumors. These are gains of 5p14, 12q13, 15q22, 17q11.2, and loss of 12p13 like in cluster c tumors (Additional file 1 and Figure 2A-d). Genes located within these tiny rearranged genomic regions are listed in Additional file 1.

“*Cluster e*” comprises tumors with a more complex pattern involving numerous chromosomal arms and regions within arms. The main rearrangements were loss of 1p with a more frequently deleted region at 1pter, gains of 1q and of 8q, and losses of 11q and of 16q. Some regions of gain or loss observed in 50% of the tumors show a more reduced segment with higher frequency of rearrangement. Specifically, these were: gain of the entire chromosome 5 with a specific gain at 5p14; loss of 6q with a specific loss at 6q16; gain of 12q with specific gain at 12q21; gain of 16p with specific gain at 16p13; gain of 17q with specific gain at 17q11; gain of 20q with a specific gain at 20q13.2. Moreover, two tiny regions show specific rearrangement. They are: gain of 4q35 and loss of 12p13 as in the two previous clusters (Additional file 1 and Figure 2A-e). Genes located at these specific loci are listed in Additional file 1.

“*Cluster f*” comprises tumors with a highly rearranged pattern. The largest recurrent changes were gain of 16p and 20q but most changes involved much smaller genomic regions scattered throughout the genomes. The large number of rearrangements did not allow any description but similar genomic regions seem involved since it is possible to identify at least 74 loci for which a genomic loss is observed in more than 80% of the tumors in this cluster (Figure 2A-f). This pattern of extreme rearrangement constituted the specter of a specific DNA breakage syndrome or DNA repair defect.

The outcome of patients belonging to these groups of tumors does not show any major difference for the five first clusters even though clusters a, b, and d show a little better prognosis than cluster c and e (Figure 2B). Conversely, patients belonging to cluster f had a very poor outcome since ten tumors out of twelve belonging to this group showed metastatic relapse during the time of the survey (Figure 2B).

### Amplicons were most common in cluster f

By defining amplicons as regions whose copy number is over three for at least two contiguous clones, 64 tumors contained at least one amplicon. A total of 90 distinct regions were amplified involving all chromosomes except chromosomes 2, 9, and 13. The number of amplicons per tumor ranged from one amplicon (13 tumors) to seventeen (one tumor). The mean was five amplicons for these 64 tumors. The size of amplicons ranged from a few kilobases containing one or a few genes as 6q25 amplification and ADR1 or 14q24.3 amplification and FOS (Additional file 1), to tens of megabases. As expected, the classic known breast cancer amplicons were the most common, including the CCND1 amplicon at 11q13 in 18 tumors, the ERBB2 amplicon at 17q12 in 17 tumors followed by 8p12 and 20q amplicons in 11 tumors.

Amplicons were seen in all the CGH clusters but their frequency varied from 32% in cluster b to 67% in cluster f (Table 2). The distribution of the number of amplicons by tumor was more specific. On average, there were only two amplicons per tumor in clusters a-e, but five in cluster f (Table 2). The increase in low level copy number changes in cluster f was thus accompanied by a corresponding increase in amplicons.

**Table 2:** Amplicons and p53 alterations according to array CGH clusters and G2I classes of tumors (%)

	**All**	**Array CGH clusters**						**G2I classes**		
		**a**	**b**	**c**	**d**	**e**	**f**	**1**	**2**	**3**
Total tumors	135	34	25	16	13	35	12	19	88	28
Tumors with amplicons	64 (47)	14 (41)	8 (32)	10 (63)	7 (54)	17 (49)	8 (67)	3 (16)	41 (47)	20 (71)
Amplicons	296	60	43	37	24	69	63	6	173	117
Amplicons per tumor (mean)	2.2	1.8	1.7	2.3	1.8	2.0	5.3	0.3	2.0	4.2
Amplicons per tumor in tumors with amplicons (mean)	4.6	4.3	5.4	3.7	3.4	4.1	7.9	2	4.2	5.9
5q23.3 ^(a)^	7 (5)	2 ( 6)	3 (12)	0	0	2 ( 6)	0	0	5 (6)	2 (7)
8p12a or 8p12-p11	11 (8)	2 (6)	1 (4)	2 (8)	3 (23)	2 ( 6)	1 (8)	0	8 (9)	3 (11)
11q13.2-q13.3	18 (13)	4 (12)	1 (4)	2 (8)	0	8 (23)	3 (25)	1 (5)	9 (10)	8 (29)
11q14.1a	11 (8)	3 (9)	0	2 (13)	0	5 (14)	1 (8)	1 (5)	7 (8)	3 (11)
12q13.11-q13.12	7 (5)	1 (3)	1 (4)	0	0	3 (9)	2 (17)	0	4 (5)	3 (11)
17q11.2b	6 (4)	1 (3)	1 (4)	0	0	2 (6)	2 (17)	0	3 (3)	3 (11)
17q12e	17 (3)	4 (12)	3 (12)	1 (4)	4 (31)	4 (11)	1 (8)	1 (5)	12 (14)	4 (14)
17q21.1-q21.2	8 (6)	2 (6)	2 (8)	0	2 (15)	2 (6)	0	1 (5)	5 (6)	2 (7)
17q21.31	8 (6)	1 (3)	0	1 (6)	2 (15)	3 (9)	1 (8)	1 (5)	5 (6)	2 (7)
17q21.33	8 (6)	1 (3)	0	1 (6)	2 (15)	2 (6)	2 (7)	0	4 (5)	4 (14)
20q ^(b)^	11 (8)	3 (9)	1 (4)	0	0	3 (9)	4 (33)	0	5 (6)	6 (21)
p53 alteration ^(c)^	39 (29)	11 (32)	5 (17)	5 (31)	4 (31)	7 (20)	7 (58)	0	24 (27)	15 (54)

^*a*)^ number of tumors (%) with the 11 most recurrent amplicons described in Additional file 2^*b*)^ tumors with at least one amplicon on 20q: at 20q11.21 , 20q13.2 or 20q13.31-q32; ^*c*)^ TP53 mutation or immunohistochemistry p53 accumulation.

### An array CGH-based index of genomic instability is predictive of clinical outcome

Due to evidence of a correlation between a highly rearranged genome (at the level of copy number variation, breakpoints, gene amplification) and clinical outcome, we built an index of genetic instability based on two parameters linked to the array CGH GNL status.

The first one corresponds to the fraction of the genome altered. It is the mean by chromosome arm of the proportion of lost or gained clones. This parameter varies from 0.004 to 0.73, with a mean value of 0.28.

The second one, corresponding to the number of altered genomic regions reflects the number of breakpoints within the genome. It is the total number of genomic regions showing a difference in copy number status with respect to the neighboring regions. In order to reduce the number of artifacts, we use a “local score” calculation to attribute a similar status (*i*.*e*. gain, normal or loss) to a genomic segment (see Additional file 2). The number of altered regions varies from 19 to 129 (mean: 64.7).

**Figure 3:**
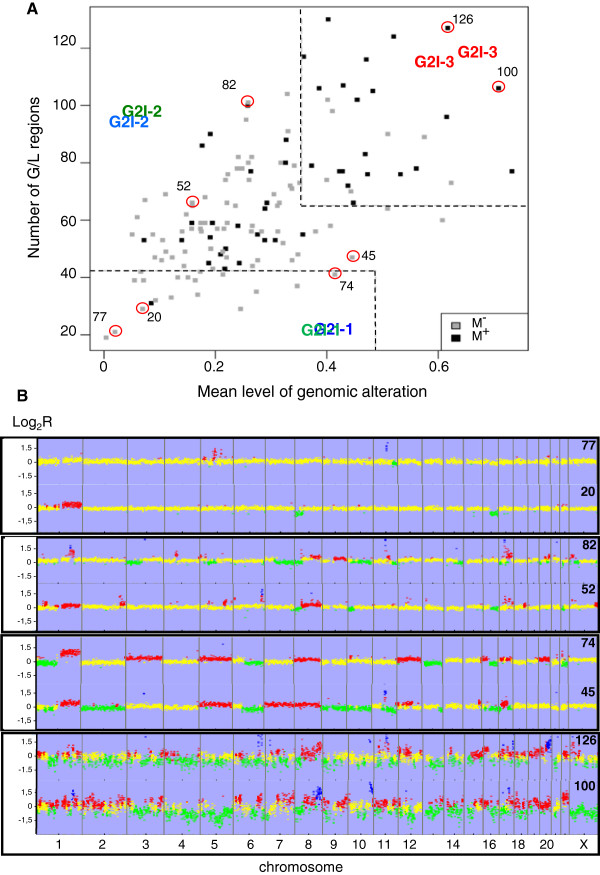
**Distribution of the tumors according to the genomic instability index (G2I).** (**A**) Scatter plot of the 135 tumors according to the two items of the G2I. Thresholds are marked by dash lines: overall level of genomic alteration < 0.48 for G2I-1 and > 0.35 for G2I-3, number of altered regions < 42 for G2I-1 and > 65 for G2I-3. M+: metastatic relapse, M- absence of relapse after 11 years of follow-up. Circles: tumors for which genomic profile is showed in B. (**B**) Examples of array CGH profiles of G2I tumors according to their position on the scatter plot. Lower left quadrant: flat profile with few rearrangements; lower right quadrant: profile with predominantly whole arm changes; upper left quadrant: profile with predominantly breakpoints; upper right quadrant: highly rearranged profile with huge number of breakpoints.

As shown in Figure 3A, the 135 tumors spread in a cloud of points with a very faint correlation between the two parameters. Tumors with chromosomal aneusomies are predominantly plotted in the lower right quadrant while tumors with numerous small rearrangements lie in the upper left quadrant (Figure 3B). In applying relapse status to each tumor (dark points on Figure 3A), it appears that the two tumoral populations (i.e. with and without relapse) show a large median overlap but that tumors lying in the lower left quadrant have a lower risk of relapse than tumors in the upper right quadrant thus individualizing three populations of tumors.

To define three grades of genomic instability, we adjusted thresholds for the two parameters that best discriminate tumors according to outcome (see the Additional file 2 for details).

Tumors in the low risk region (G2I-1 for Genomic Instability Index - grade 1) showing an overall level of genomic alteration below 48% and a number of altered regions < = 42, relapsed in one case out of 19. Tumors in the high risk region (G2I-3, for Genomic Instability Index - grade 3) showing an overall level of genomic alteration above 35% and a number of altered regions > = 65 relapsed in 21 out of 28 cases. The difference in risk of relapse between the G2I-1 and G2I-2 tumors was borderline significant (Odd ratio: 0.16 [0.2-1.2] p = 0.08) whereas that between the G2I-2 and G2I-3 tumors was highly significant (odd ratio: 8.5 [3.2-22.6] p < 0.001) in univariate analysis. Similar results were obtained in multivariate analysis adjusted on the Nottingham Prognostic Index (NPI) (Table 3). The contribution of each CGH cluster class to the three G2I groups is shown in Table 4 and examples of array CGH profiles from the four quadrants of the scatter plot are provided in Figure 3B.

**Table 3:** Association with relapse in the study group for the G2I and for various clinicopathological parameters

				**Univariate analysis**		**Multivariate analysis**	
**Clinicopathological parameters**	**All**^**(a)**^**n(%)**	**No Relapse n(%)**	**Relapse n(%)**	**OR [CI 95%]**	**p**	**OR [CI 95%]**	**p**
**G2I**^(b)^							
Class 1	19 (14)	18 (20)	1 (2,2)	0,16[0,2-1,2]	0,08	0,24[0.03-2]	0.19
Class2	88 (65)	65 (72,2)	23 (51,1)	1		1	
Class3	28 (21)	7 (7,8)	21 (46,7)	8,5[3 ,2-22,6]	<**0**,**001**	11.9[4.2-34.1]	<**0**.**001**
**SBR Grade**^(c)^						rejected	
1	25 (18)	21 (23,3)	4 (8,9)	1			
2	71 (53)	46 (51,1)	25 (55,6)	2,9[0,9-9,2]	0,08		
3	39 (29)	23 (25,6)	16 (35,6)	3,7[1,05-12,7]	**0**,**04**		
**Histological size**						rejected	
≤ 20 mm.	90 (67)	67 (74,4)	23 (51,1)	1			
> 20 mm.	45 (33)	23 (25,6)	22 (48,9)	2,8[1, 3–5, 9]	**0**,**008**		
HR status ^(d)^							
HR^-^	19 (14)	12 (13,3)	7 (15,6)	1,19[0 ,44-	0,72		
HR^+^	116 (86)	78 (86,7)	38 (84,4)	3,3]			
NPI ^(e)^							
1 + 2	30 (22)	24 (26,7)	6 (13,3)	1		1	
3	85 (63)	58 (64,4)	27 (60)	1,8[0,7-5,1]	0,23	1.38[0.4-4.4]	0.6
4	20 (15)	8 (8,9)	12 (26,7)	6[1,7-21,3]	**0**,**006**	7.2[1.8-29.3	**0**.**006**
TP53 alteration							
no	115 (85)	73 (81,1)	31 (68,9)	1			
yes	20 (15)	17 (18,9)	14 (31,1)	1,9[0,9-4,4]	0,11		
**mib1**^(f)^						rejected	
<20%	93 (69)	67 (74,4)	26 (57,8)	1			
≥20%	42 (31)	23 (25,6)	19 (42,2)	2,1[1–4,5]	**0**,**05**		
**IHC intrinsic class**^(g)^							
LA	83 (62)	59 (67)	24 (53,3)	1			
LB + Her2+ Basal	50 (38)	29 (33)	21 (37,6)	1,8[0,85-3,7]	0,12		
nd	2	2					

^(*a*)^ n: Number of tumors; % in parentheses; ^(*b*)^ G2I: Three classes of genomics instability index as defined here; ^(*c*)^ SBR: Scarff, Bloom and Richardson grade; ^(*d*)^ HR: Hormonal Receptor; HR^-^: negative hormonal receptor status evaluated by immunohistochemistry (IHC) with a threshold of 10% of tumors cells; ^(*e*)^ NPI: Nottingham Prognostic Index, 1 + 2: good or excellent, 3: moderate, 4: poor; ^(*f*)^ immunohistochemistry expression of mib1; ^(*g*)^ immunohistochemical intrinsic class: LA: Luminal A for HR^+^-tumors with mib1 expression in less than 20% of tumor cells, LB: Luminal B for HR^+^-tumors with mib1 expression in at least 20% of tumor cells or with Her2 score of 3, Her2: Her2-enriched tumors for HR^-^ and Her2^+^-tumors, Basal for HR^-^ and Her2^-^-tumors that express at least one of the following protein in more than 10% of tumor cells: egfr, ck5/6, or vimentin;

**Table 4:** distribution of the three classes of G2I according to array-CGH clusters (%)

	**Cluster a n = 34**	**Cluster b n = 25**	**Cluster c n = 16**	**Cluster d n = 13**	**Cluster e n = 35**	**Cluster f n = 12**	**All n = 135**
G2I-1	6 (18)	6 (24)	3 (19)	1 ( 8)	3 ( 9)	0	19 (14)
G2I-2	26 (76)	19 (76)	10 (62)	12 (92)	21 (60)	0	88 (65)
G2I-3	2 ( 6)	0	3 (19)	0	11 (31)	12 (100)	28 (21)

### Validation of the G2I on an independent data set

**Figure 4:**
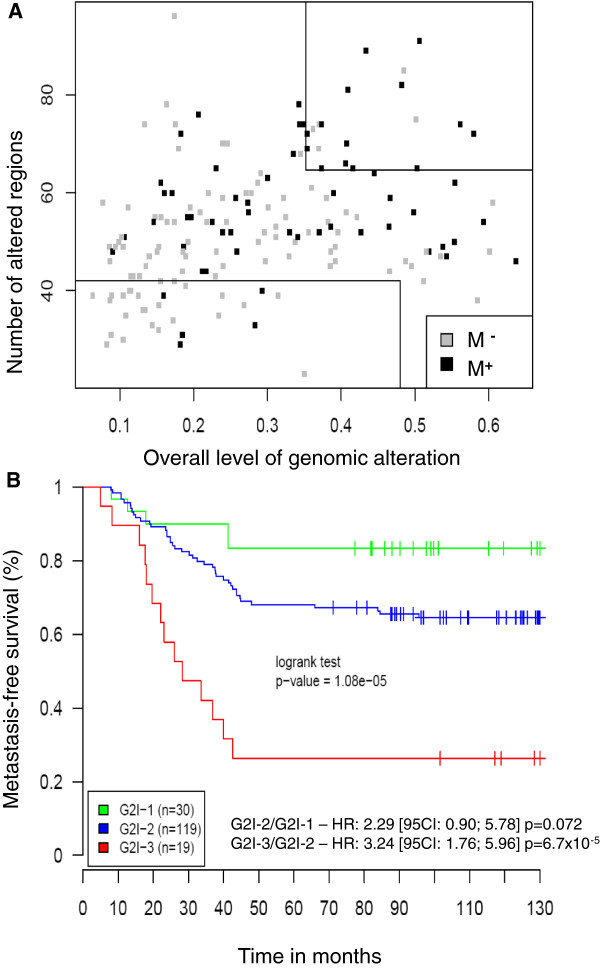
**Prognostic value of the G2I on independent data set.** (**A**) Scatter plot of 168 breast cancers from which BAC array CGH data were available [21] according to the two items and the previously defined thresholds of the G2I. M^+^: metastatic relapse; M^-^: absence of local or metastatic relapses after five years of follow-up. (**B**): Kaplan Meier curves showing metastasis-free survival at 10 years for the three G2I classes. Green: G2I-1, blue: G2I-2, red: G2I3; the number of tumors in each G2I class is given in parenthesis. HR: hazard ratio (log-rank test).

To validate the G2I on independent data, we analyzed 168 breast cancers without axillary lymph node involvement for which BAC array CGH data were available [21]. In this dataset, 57 patients developed metastases while 111 others did not show metastatic or loco regional recurrence after at least 5 years of follow-up (median follow-up: 10.8 years). Using the previously defined thresholds, the G2I could predict clinical outcome with a p-value of 1.08x10^-5^ (logrank test) since, among tumors scored as G2I-3, 74% developed metastases, whereas in the G2I-1 group only 16% did (Figure 4A). The ten year metastasis–free survival (Figure 4B) analyzed with the log-rank test showed a highly significant difference between (i) the G2I-3 and G2I-2 groups (hazard ratio: 3.24 [95CI: 1.76-5.96] p = 6.7x10^-5^ and (ii) a borderline significant difference between the G2I-1 and G2I-2 groups (hazard ratio: 2.29 [95CI: 0.90-5.78] p = 0.072).

### Comparison of the G2I and the array CGH clusters with classical prognostic parameters

The tumors used in this study were matched for age and axillary lymph node involvement but not for other factors, such as the size of the tumors, the histological Scarff Bloom and Richardson (SBR) grade or the hormonal receptor (HR) status.

A search for correlations between the G2I and classical prognostic factors did not show any correlation for histological size, steroid hormone receptor status, or Nottingham prognostic index (NPI) (Table 5). There was a correlation with the intrinsic classification although the basal tumors belong mainly to the G2I-2 group (Table 6) and with the Mib1 status suggesting a link with proliferation (Table 5).

**Table 5:** Association between G2I classes and clinico-pathological parameters

**Clinicopathological Parameters**	**Total n = 135 (%)**	**G2I-1 19**	**G2I-2 88**	**G2I-3 28**	**χ**^**2**^**test *p*-value**
Lymph node involvement					
pN0	75 (56)	12 (63)	43 (49)	20 (83)	0.086
pN^+^	60 (44)	7 (37)	45 (51)	8 (29)	
Age (years)					
<=55	70 (52)	8 (42)	51 (58)	11 (39)	0.149
>55	65 (48)	11 (58)	37 (42)	17 (61)	
Histological size (mm)					
<= 20	90 (67)	16 (84)	57 (65)	17 (61)	0.2
> 20	45 (33)	3 (16)	31 (35)	11 (39)	
Scarff Bloom Richardson (SBR) Grade					
1	25 (18)	6 (32)	15 (17)	4 (14)	0.037
2	71 (53)	12 (63)	48 (55)	11 (39)	
3	39 (29)	1 ( 5)	25 (28)	13 (39)	
Steroid Hormone Receptor status					
HR^+^ : > = 10%	116 (86)	18 (95)	74 (84)	24 (86)	0.48
HR^-^	19 (14)	1 ( 5)	14 (16)	4 (14)	
Nottingham prognostic index (NPI)					
1 + 2	30 (22)	6 (32)	20 (23)	4 (14)	0.37
3	85 (63)	13 (68)	50 (57)	22 (79)	
4	20 (15)	0 ( 0)	18 (20)	2 ( 7)	
Mib1					
<20%	93 (69)	18 (95)	60 (68)	15 (54)	**0**.**011**
≥20%	42 (31)	1 ( 5)	28 (32)	13 (46)	
Ihc intrinsic classification^(a)^					
LA	83 (62)	16 (84)	55 (64)	12 (43)	**0**.**014**
LB + her2+ Basal	50 (38)	3 (16)	31 (36)	16 (57)	
unclassified	2	0	2	0	
p53 status ^(b)^					
-	95 (71)	19 (100)	64 (73)	13 (46)	**0**.**0003**
+	39 (29)	0 ( 0)	24 (27)	15 (54)	
At least one amplicon					
No	71 (53)	16 (84)	47 (53)	8 (29)	**0**.**001**
Yes	64 (47)	3 (16)	41 (47)	20 (71)	

^(a)^ immunohistochemistry based on intrinsic classification, LA: luminal A, LB: luminal B, Her2: Her2-enriched; ^(b)^ TP53 mutation or immunohistochemical detection of p53 accumulation.

**Table 6:** Distribution of the tumors within the immunohistochemistry intrinsic classes according to SBR grade and array CGH-based genomic characteristics

	**All n = 135 (%)**	**Luminal A n = 83**	**Luminal B n = 33**	**HER2-enriched n = 10**	**Basal n = 7**	**Unclassified n = 2**
SBR Grade						
1	25 (18)	24 (29)	0	0	1 (14)	0
2	71 (53)	53 (64)	16 (49)	1 (10)	0	1
3	39 (29)	6 ( 7)	17 (52)	9 (90)	6 (86)	1
Array CGH clusters						
a	34 (25)	17 (20)	5 (15)	5 (50)	6 (86)	1
b	25 (18)	20 (24)	3 ( 9)	2 (20)	0	0
c	16 (12)	7 ( 8)	8 (24)	0	0	1
d	13 (10)	9 (11)	3 ( 9)	1 (10)	0	0
e	35 (26)	23 (28)	11 (33)	1 (10)	0	0
f	12 ( 9)	7 ( 8)	3 ( 9)	1 (10)	1 (14)	0
Nb. of amplicons						
0	71 (53)	56 (67)	7 (21)	1 (10)	6 (86)	1
≥1	64 (47)	27 (33)	26 (79)	9 (90)	1 (14)	1
Range	1–17	1–10	1–17	1–11	NA	1
Mean	4.9	3.6	5.6	4.9	9	1
G2I classes						
1	19 (14)	16 (19)	2 ( 6)	1 (10)	0	0
2	88 (65)	55 (66)	19 (58)	6 (60)	6 (86)	2
3	28 (21)	12 (15)	12 (36)	3 (30)	1 (14)	0

Interestingly, the G2I-3 remains associated with relapse with respect to the G2I-2 group in the following subgroups: tumors smaller than 20 mm (OR: 5.6 [1.7-17] p = 0.004); SBR grade 2 and 3 tumors (OR: 9.9 [1.9-51.5] p = 0.006 and OR: 10.6 [2.2-51.4] p = 0.004 respectively); steroid hormone receptor-positive tumors (OR: 8.37 [2.9-24.2] p < 0.001); NPI class 3 (moderate) tumors (OR: 14.5 [3.5-60.8] p < 0.001) and axillary lymph node negative tumors (OR: 17.5 [4.6-66.7] p < 0.001). This last result reflects the higher proportion of node negative tumors in the G2I-3 group than in the G2I-2 group (71% and 49% respectively; Additional file 4). Overall, tumors with grade 3 genetic instability were mainly luminal and lacked axillary lymph node involvement, but had a very high risk of metastatic relapse.

### Correlation with TP53 mutations

Because of the link between p53 and genome stability, we searched for alterations of the TP53 gene directly by DNA sequencing and indirectly by immunohistochemistry (IHC) for increased p53 protein expression. Point mutations were detected in 31 tumors (20 missense mutations and 11 truncated mutations) and p53 IHC expression was detected in 28 tumors with a good correlation between missense mutation and protein expression (Table 7).

**Table 7:** p53 alterations

**IHC status of p53**^**(a)**^	**Mutation status of *TP53***^**(b)**^		**Type of TP53 mutation**^**(c)**^		**All (%)**
	-	+	ms	tm	
-	96	11	1	10	107 (80)
^+^	7	20	19	1	27 (20)
All (%)	103 (77)	31 (23)	20 (15)	11 (8)	134 (100)

^(a)^ +: Immunohistochemical detection of p53 accumulation in at least 10% of tumor cells, -: no p53 signal; ^(b)^ +: detection of a mutation of *TP53* (listed in Additional file 2), -: wild type TP53; ^(c)^ ms: missense mutation, tm: truncated mutation (frameshift or nonsense).

The presence of a TP53 alteration (either a mutation or an increase in protein detection) was correlated with the G2I (Table 5) (p =0.0003). No TP53 alterations were detected in the G2I-1 tumors compared with TP53 alterations which were found in 54% of the G2I-3 tumors (Table 2). TP53 alterations were observed in all CGH clusters with a frequency of 20-30% of tumors in clusters a to d compared with 58% for tumors in the highly unstable cluster f (Table 2). The expected pattern of TP53 alterations was seen with respect to intrinsic classification: mutations or increased protein expression were seen in 70%, 57%, 42% and 15% of HER2-enriched, basal- like, luminal B and luminal A tumors, respectively.

### Genomic rearrangements specific to G2I-3 tumors

Tumors belonging to the G2I-3 group showed a high level of genetic alteration with a large number of small regions showing copy number variation, mainly losses. Some alterations were recurrent and specific to this group indicating a possible selection for these rearrangements. Additional file 4 shows the frequencies of gain and loss for each clone in the three G2I groups. Genomic regions showing significantly more gains or losses in the G2I-3 tumors compared with the two other groups of tumors with a p value < = 10^-4^ and a frequency of ≥ 50% are listed in Additional file 1. Six regions on chromosomes 12, 16, 17, and 20, show specific gains for the G2I-3 tumors, and a further 49 regions show specific genomic losses. Most regions contained multiple genes but a few were small enough to allow identifying potential driver genes mentioned in Additional file 1.

### A gene expression signature specific to G2I-3 tumors

High quality RNA was available for 46 of the 135 tumors. Fifteen of these belong to the G2I-3 group, 29 to the G2I-2 group, and two tumors to the G2I-1 group. We hybridized cDNA from these tumors to Affymetrix U133 Plus 2.0 genechips. Supervised analysis allowed us to define a signature of 300 probe sets showing differential expression between 14 of the 15 G2I-3 tumors and the rest (Additional file 4). The list of genes for which over or under expression is specific for G2I-3 tumors is provided in Additional file 1. The genes in this signature are not specifically linked to cell proliferation or to any DNA repair system. Several of the genes over-expressed in G2I-1 + 2 tumors are involved in signal transduction, in particular the hedgehog, VEGF and MAPK pathways (Additional file 1). The genes best distinguishing G2I-2 from G2I-3 tumors are not specifically localized at rearranged genomic regions (Additional file 1). However, several over-expressed genes belonging to this signature are located at genomic regions specifically gained in G2I-3 tumors such as JAG1 at 20p12.2 or RPN2, C20orf117, and DHX35 at 20q11.23. Conversely, under-expressed genes are located at specifically lost regions such as CD 109 at 6q13, ELOVL4 at 6q14.1, C9orf46, KIAA1432 and CDC37L1 at 9p24.1, LAMA1 and DLGAP1 at 18p11.31 or DSG3 at18q12.1 suggesting a gene dosage effect in the constitution of a part of the signature.

**Figure 5:**
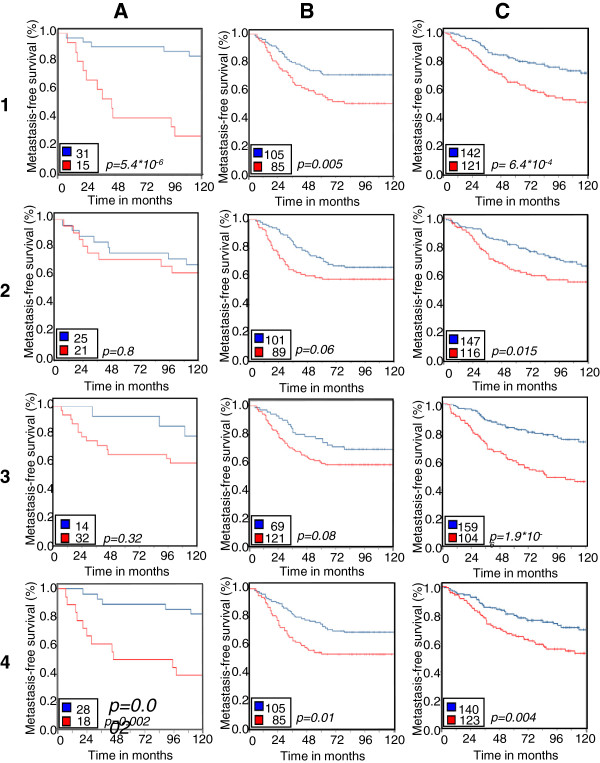
**comparison of prognostic value of the G2I transcriptomic signature with three other prognostic signatures.** Kaplan Meier curves showing the prognostic value of the G2I transcriptomic signature (line 1), of the 70-gene Amsterdam signature (line 2), of the Gene expression Grade Index (line 3) and of the intrinsic subtypes (line 4) on three independent sets of tumors. Column **A**: this study, column **B**: the Rotterdam study [25], column **C**: the study by Loi et al. [26] .

To test whether this signature has independent prognostic value, we compared it to three previously published prognostic signatures in three independent data sets including ours. The results are summarized in Figure 5. The Amsterdam signature [22], the genomic grade index (GGI) [23], and the intrinsic gene set [11] were all able to split the tumors into two groups according to outcome in the three sets of tumors. As expected, the G2I signature gave the best results for our own data set (p = 5.4x10^-6^) compared to the results for these tumors with the Amsterdam, GGI and intrinsic signature (p = 0.8; p = 0.32; and p = 0.002 respectively). The G2I transcriptomic signature also showed higher prognostic value than the three other signatures in the Rotterdam study [25]. It showed higher prognostic value (p = 6.4x10^-4^) than the Amsterdam and intrinsic signature (p = 0.015 and p = 0.004 respectively) in the Loi study [26] but the GGI signature gave the best results in these tumors, on which it was trained (Figure 5). We conclude that genomic instability is an important marker of poor prognosis whether it is assessed directly with CGH data or indirectly with gene expression data.

## Discussion

Array CGH analysis of breast carcinoma both on BAC array and on oligo array had previously highlighted the genomic heterogeneity of these tumors. The most popular classification distinguishes three classes of tumors. The first one, characterized by only few rearrangements is called “simplex” [1,27] or “1q/16q” [28,29], the second, called “complex sawtooth” [27,30] or “complex” [28,29], is characterized by a large number of rearrangements, including breakpoints and copy number variations for very small genomic segments. The third one called “complex firestorm” [27,30] or “mixed amplifier”, [28,29] is characterized by a phenomenon of gene amplification with a high copy number variation restricted to small genomic regions. Indeed, it is possible to allocate some specific tumors to such a class of genomic profiles and for example, tumor number 83 in our series showed a simplex profile with only a 1q gain and 11q and 16 q losses as sole rearrangements, tumor number 43 showed a typical complex sawtooth profile, and tumor number 100 a mixed amplifier profile. However, a large number of tumors in our series showed intermediary patterns and it was not possible to assign them to a specific class. For example, tumor number 7 showed a relatively flat profile with several amplicons on chromosomes 6 and 17 and tumor number 47 showed an intermediary profile between simplex and complex sawtooth.

In fact, three kinds of genomic rearrangements related to various kinds of genetic instability are detectable by array CGH methodologies. They are: i) whole chromosomal or whole chromosomal arm aneusomies related respectively to mitotic malsegregation or centromeric rearrangement, ii) DNA breakpoints with repair defects resulting in copy number variation for short genomic segments and iii) gene amplification. These three kinds of genomic rearrangements are more or less associated in a single tumor and show a continuous variation with a growing level of intensity from one tumor to another. Thus, a true classification based on genomic alteration criteria remains difficult to implement. The results obtained here suggest that it is possible to distinguish between two groups of tumors. One group shows gain or loss of entire chromosomes or entire chromosomal arms but lack breakpoint within the affected regions. This group corresponds to tumors from the clusters b to e which are characterized by combinations of specific rearranged chromosomal arms. The second group corresponds to tumors from the clusters a and f for which it is not possible to identify a copy number variation affecting an entire chromosome arm, either because of a flat profile (cluster a) or because of a huge number of DNA breakpoints (cluster f). In order to take into account this distinction, we constructed a genomic index based on two parameters representing these two kinds of alterations and showing a continuous distribution of the tumors with a growing level of alterations (Figure 3). Adverse outcome was observed for the most highly rearranged genotypes, corresponding mainly to tumors from clusters e and f.

The transcriptomic intrinsic classification of breast cancer [10] has led to search for correlations between the Sorlie classes and specific genomic profiles. It was effectively possible to correlate the luminal A class with the simplex profile, the luminal B and the Her2-enriched classes with the amplifier profile and the basal-like class with the complex sawtooth profile [1,28,31]. Moreover, a new classification into six classes taking into account these correlations was recently proposed [32]. Such a correlation was also found here between immunohistochemical intrinsic classes and genomic profile. Both the G2I-1 group and the cluster b (1q gain, 16q loss) are mainly composed of luminal A tumors (84% and 80%, respectively). The majority of the tumors belonging to luminal B and Her2-enriched classes show gene amplifications (79% and 90%, respectively). Some results, conversely, are more surprising. If percentages of luminal A tumors decrease progressively from the G2I-1 to the G2I-2 and G2I-3 groups (respectively, 84%, 62.5% and 43%), the fact that 12 luminal A tumors belong to the G2I-3 group was not expected. In the same way, it is surprising that seven of twelve cluster f tumors belong to the luminal A class. Seven tumors belong to the basal-like class. Only one of them appears in the cluster f and in the G2I-3 group. The six other basal-like tumors all belong to the G2I-2 group and to the array CGH cluster a. This cluster, without any specific chromosomal aneusomy, contains in fact two subgroups (Figure 1). The first one (right branch) shows tumors with a flat profile belonging mainly to the luminal A class. The second one (left branch) including the six basal-like tumors shows tumors without chromosomal or chromosomal arm aberrations but with copy number changes affecting small genomic regions that are different from one tumor to another. This profile corresponds to the previously described subtype of high grade ER-negative tumors with low genomic instability index [33]. The fact that six out of seven basal-like tumors did not show metastatic relapse is probably related to a series effect with a small number of cases. It therefore seems that some breast carcinomas of luminal A and luminal B phenotypes, showing important genetic instability with a large number of DNA breakpoints, frequent TP53 mutations, and frequent gene amplification are characterized by very poor outcome.

Prognostic value of genomic alteration in breast cancer has often been reported. Cytogenetic analysis had previously shown the correlation between the unbalanced der(1;16) and good prognosis [34], whereas homogeneously staining regions or gene amplifications were correlated with poor outcome [35,36]. These results were confirmed by array CGH approaches that show associations between gene amplification in Her2-enriched and luminal B classes [28,31,37,38], and poor prognosis or between 16q loss in luminal A tumors and good prognosis [39]. Subsequently, copy number variation concerning various genomic regions was shown to be related to outcome as loss on chromosome arms 19 and 18q [40] or more complex signatures including several regions, either distinct for ER positive and negative tumors [41], or common for these two kind of tumors [21]. The measurement of genetic instability was not so well documented. A signature of chromosome instability was inferred from transcriptomic data as functional aneuploidy related to a clear deviation in expression of contiguous genes from the same loci [17]. The application of this signature to four different published sets of breast cancer was highly predictive of outcome [17]. The fraction of the genome altered (FGA), calculated as the number of probes affected by gain or loss compared to the total number of probes represented on the array [42], was shown to correlate with the classification proposed by Jonsson et al. in which a higher level of FGA was observed for “basal complex” and “luminal complex” types of tumors than for the others [32]. The FGA after correction for tumoral cellularity and named “genome instability index” (GII) fails to find such a correlation but identified a subtype of basal like tumor with low instability [33]. In association with a three chromosomal region predictor, the CGH classifier proposed by Gravier et al. in node negative breast cancer used a measurement of genomic complexity corresponding after segmentation to the total number of segmental alterations along the genome with a threshold of 11. Using this single parameter, the prediction of metastatic relapse was highly significant (p = 0.00056) [21]. Recently, an array CGH-based score of genomic complexity called CAAI (Complex arm aberration index) was shown to have overall independent prognostic power [43]. All these data indicate that the type and the level of genetic instability are major determinants of outcome for breast cancer. These characteristics are probably set up very early during tumor development, conserved at late stages and common to any tumoral cell. They can be detected at the level of a primary tumor, even if only some cell clones will acquire metastatic power. The same explanation could be offered for the prognostic significance of transcriptomic signatures obtained from primary tumors that have been shown to be mainly related to the proliferative activity of the tumors [44].

From a clinical point of view, it is interesting to note that the prognostic value of the G2I is independent of other major prognostic factors except TP53 mutation (Table 5). A faint correlation is also found with others genomic alterations (in particular, the presence of amplicons), with the intrinsic classification and with Mib1 index but not for classical clinico-pathological parameters (Table 5). Moreover, the G2I maintains a strong predictive value in subclasses of tumors showing variable outcomes, such as small tumors, SBR grade 2 and 3 tumors, hormonal receptors positive tumors and tumors in the moderate class of the NPI. These data are in favour of an independent prognostic value for the G2I but evaluation of the benefit in clinical practice will require better definition of the thresholds used to define the groups and validation on an unselected population-based set of tumors. These investigations are currently in progress. The main result concerns the strong predictive value of the G2I in tumors without axillary lymph node involvement since 80% of G2I3 node negative tumors (16 out of 20) relapsed, whereas only 16% of the G2I-1 and G2I-2 node negative tumors (9 out of 55) did so (OR: 17.5 [4.6-66.7] p < 0.001). This information could have major implications for the indication of adjuvant therapies. The paradox of a poor outcome for tumors that do not show any evidence of lymphatic dissemination at the time of local treatment may suggest that these tumors with high genetic instability are not lymphophilic, instead showing a hematogenic mode of diffusion.

## Conclusion

Accurate evaluation of genetic instability allows the identification of a previously unrecognized group of breast cancers in which a DNA repair defect is probably involved. From a clinical perspective, the high metastatic risk observed for this class of tumors indicates that their treatment should include adjuvant therapies.

## Abbreviations

G2I: Genomic instability index; TMA: Tissue microarray; ER: Estrogen receptor; PR: Progesterone receptor, gnl, gain normal, loss; SBR: Scarff Bloom and Richardson; HR: Hormonal receptor; NPI: Nottingham prognostic index.

## Competing interests

The authors declare that they have no competing interests.

## Authors’ contributions

ML, FB, GMG and NS planned and supervised the work; CTL and MD provided tumor samples and clinical data; FB, CP and GB performed and interpreted the array CGH experiments; BO and CT contributed to the array-CGH methodology; SS and NJ performed TP53 analysis; GMG and IdM interpreted immunohistochemistry data; VB and MG performed statistical and bioinformatic analyses; AdR supervised bioinformatic analyses; FB, NJ and NE managed the data; FB, MG, AdR, GMG and ML wrote the manuscript. All authors read and approved the final manuscript.

## Pre-publication history

The pre-publication history for this paper can be accessed here:

http://www.biomedcentral.com/1755-8794/5/54/prepub

## Supplementary Material

Additional file 1 **Supplementary tables.** File containing six supplementary tables; samples annotations for the 135 studied tumors; incidence of the most frequent genomic gains and losses with respect to array-CGH clusters of tumors; description of genomic regions of amplification; description of recurrently gained and lost genomic regions in G2I-3 tumors; list of probe sets defining the transcriptomic signature of the G2I-3 tumors; Gene Ontology enrichment for the G2I-3 expression signature. Click here for file

Additional file 2 **Supplementary methods.** File containing methodological details. Click here for file

Additional file 3 **G2I_R tool.** File containing the R script used to generate the G2I with the tables of data and the instructions useful to reproduce the results. Click here for file

Additional file 4 **Supplementary figures.** File containing three supplementary figures; scatter plot of the 135 tumors according to the G2I and axillary lymph node involvement; frequency plot of genomic copy number variation in tumors belonging to the 3 classes of G2I; Hierarchical clustering showing the differential expression of 300 probe sets between G2I-3 and G2I-2 tumors. (PDF 279 kb) Click here for file
