# Supplementary material for: An array CGH based genomic instability index (G2I) is predictive of clinical outcome in breast cancer and reveals a subset of tumors without lymph node involvement but with poor prognosis
Source: BMC Med Genomics. 2012 Nov 27;5:54. doi: 10.1186/1755-8794-5-54 (PMC3558323; doi:10.1186/1755-8794-5-54)
Supplement: Additional file 1 — Supplementary tables. File containing six supplementary tables; samples annotations for the 135 studied tumors; incidence of the most frequent genomic gains and losses with respect to array-CGH clusters of tumors; description of genomic regions of amplification; description of recurrently gained and lost genomic regions in G2I-3 tumors; list of probe sets defining the transcriptomic signature of the G2I-3 tumors; Gene Ontology enrichment for the G2I-3 expression signature. [file 1755-8794-5-54-S1.pdf]

Supplementary table 1: samples annotations

ID [(a)]	age<br><br>(years)	SBR<br><br>Scarff, Bloom and Richardson grade	tumor size<br><br>(mm)	relapse<br><br>0 = no<br>1 = yes	delay<br><br>(months)	pN histo<br><br>Lymph node invasion<br>0 = negative<br>1 = positive	ER status (IHC[b])<br><br>ER: estrogen receptor<br>0: <10% cells<br>1: >=10% cells	PR status (IHC)<br><br>PR: progesteron receptor<br>0: <10% cells<br>1: >=10% cells	NPI (group)<br><br>1=excellent<br>2=good<br>3=moderate<br>4 = poor	Her2 status (IHC)<br><br>0: absence of signal<br>1-2-3: expression in at least 10% of cells with signal intensity of 1, 2 or 3	mib-1 status (IHC)<br><br>% of tumor cells expressing mib-1	IHC phenotype<br><br>Luminal A: HR =1, Her2 <3 and mib1 <20<br>Luninal B: HR=1, Her2<3 and mib1 >=20<br>Hrt2-enriched: Her2 =3<br>basal:ER and PR <10% ; Her2 <3; pcad or egfr or ck5/6 or vimentin: pos<br>nd:unclassified	array-CGH cluster	number of Amplicons	Number of altered regions<br><br>N	Overal level of genomic alterations<br><br>A	G2I<br><br>G2I-1=A<0,4876 and N<42,3638<br><br>G2I-3=A>0,3546 and N>65,0786	p53 status (IHC)<br><br>0: absence of signal<br>1: signal in at least 10% of tumor cells	TP53 mutation<br><br>0 : absence of mutation.<br>1: frameshift or nonsense mut.<br>2: misense mut.<br>nd:unclassified	description of TP53 mutation
RELB_001	42	2	20	0	132	0	1	1	2	0	4	luminal A	b	0	44	0,14	2	0	0	
RELB_002	40	3	25	1	28	1	0	0	4	0	55	basal	a	0	99	0,26	2	1	0	
RELB_003	77	2	15	0	132	1	1	0	3	0	20	luminal A	e	3	59	0,17	2	0	0	
RELB_004	40	1	15	0	132	0	1	1	1	0	5	luminal A	a	0	69	0,11	2	0	0	
RELB_005	47	2	25	0	132	1	1	1	3	0	10	luminal A	e	0	82	0,24	2	0	0	
RELB_006	52	1	17	0	132	0	1	1	2	0	3	luminal A	b	9	104	0,33	2	0	0	
RELB_007	46	2	18	0	132	0	1	1	2	3	15	luminal B	b	3	55	0,05	2	0	0	
RELB_008	46	1	15	0	132	0	1	1	2	0	2	luminal A	c	0	40	0,13	1	0	0	
RELB_009	54	3	27	1	68	1	1	1	4	0	26	Luminal B	e	0	54	0,28	2	0	0	
RELB_010	49	2	20	0	132	0	1	1	3	3	5	luminal B	d	3	42	0,17	1	0	0	
RELB_011	61	2	20	0	132	0	1	1	2	0	6	luminal A	d	0	49	0,11	2	0	0	
RELB_012	77	1	15	0	132	1	1	0	3	0	7	luminal A	b	0	67	0,07	2	0	0	
RELB_013	65	1	35	0	132	1	1	1	3	0	5	luminal A	a	0	80	0,26	2	0	0	
RELB_014	65	2	15	0	132	0	1	0	2	1	25	Luminal B	b	10	91	0,33	2	0	0	
RELB_015	49	2	22	1	34	0	1	1	3	0	21	Luminal B	e	3	106	0,43	3	0	0	
RELB_016	62	2	17	0	132	0	1	1	2	0	11	luminal A	e	3	72	0,62	3	0	0	
RELB_017	64	2	22	1	129	0	1	1	3	0	10	luminal A	e	0	76	0,73	3	0	2	Ex7 c.747 G>C R249S
RELB_018	54	2	20	0	132	1	1	1	3	0	2	luminal A	b	0	76	0,22	2	0	0	
RELB_019	53	3	30	1	98	1	1	0	4	0	22	Luminal B	e	5	105	0,39	3	0	0	
RELB_020	58	1	22	0	132	1	1	1	3	0	9	luminal A	b	0	29	0,07	1	0	0	
RELB_021	69	3	14	1	37	0	0	0	3	3	22	Her2-enriched	e	3	76	0,47	3	0	0	
RELB_022	38	3	14	0	132	1	1	1	3	0	22	Luminal B	c	0	47	0,12	2	0	0	
RELB_023	60	2	20	0	132	0	1	0	3	0	8	luminal A	e	0	47	0,09	2	0	0	
RELB_024	69	2	25	0	132	0	1	0	3	0	12	luminal A	e	1	78	0,25	2	0	0	
RELB_025	63	3	25	0	132	0	0	0	3	0	22	basal	a	0	72	0,35	2	0	0	
RELB_026	49	1	12	0	132	0	1	1	2	0	4	luminal A	a	0	74	0,22	2	0	0	
RELB_027	45	2	22	1	111	0	1	0	3	2*	27	luminal B	e	8	86	0,18	2	1	2	Ex8 c.818 G>A R273H
RELB_028	51	1	13	0	132	1	1	1	3	0	4	luminal A	a	0	66	0,16	2	0	0	
RELB_029	65	3	18	0	132	1	1	0	3	3	17	luminal B	c	11	90	0,51	3	1	2	Ex7 c.743 G>A R248Q<br>Ex4 c.98 C>A S33Y
RELB_030	60	3	16	0	132	1	1	0	3	0	13	luminal A	e	0	95	0,26	2	0	0	
RELB_031	47	2	25	1	46	1	1	1	3	0	35	Luminal B	e	0	78	0,37	3	0	0	
RELB_032	37	2	13	0	132	0	1	1	2	0	12	luminal A	a	0	58	0,27	2	0	0	
RELB_033	70	3	18	0	132	0	0	0	3	3	15	Her2-enriched	a	11	102	0,40	3	1	0	
RELB_034	49	2	13	0	132	0	1	1	2	0	16	luminal A	a	0	61	0,06	2	0	0	
RELB_035	52	1	15	0	132	1	1	0	3	0	3	luminal A	a	0	42	0,07	1	0	0	
RELB_036	40	3	20	1	47	0	1	1	3	0	21	Luminal B	c	0	79	0,33	2	0	0	
RELB_037	64	1	25	1	83	1	1	0	4	0	15	luminal A	a	3	59	0,16	2	0	0	
RELB_038	49	3	20	1	8	1	0	0	4	3	45	Her2-enriched	b	7	53	0,14	2	0	1	Ex8 c.898delC P301fsX44
RELB_039	65	3	15	0	132	0	0	0	3	0	26	unclassified	c	1	57	0,20	2	1	2	Ex5 c.488 A>G Y163C
RELB_040	55	2	30	1	45	0	1	1	3	0	27	Luminal B	e	2	72	0,44	3	1	nd	
RELB_041	66	2	17	1	66	1	1	1	3	3	15	luminal B	e	7	78	0,56	3	0	1	Ex8 c.868_891del24insA K291fsX7
RELB_042	43	2	15	0	132	1	1	1	3	0	17	luminal A	a	8	57	0,16	2	0	0	
RELB_043	48	1	16	1	111	0	1	1	1	0	0	luminal A	f	0	129	0,40	3	0	0	
RELB_044	66	3	20	1	23	0	0	0	3	3	32	Her2-enriched	f	9	105	0,48	3	1	2	Ex8 c.818 G>A R273H
RELB_045	66	2	20	0	132	1	1	1	3	1	16	luminal A	e	2	47	0,45	2	0	0	
RELB_046	52	1	14	0	132	1	1	1	3	0	0	luminal A	a	0	78	0,34	2	0	0	
RELB_047	53	2	12	0	132	0	0	0	2	2	18	unclassified	a	0	59	0,18	2	0	0	
RELB_048	65	2	30	1	59	1	1	1	4	0	2	luminal A	e	0	53	0,29	2	0	0	
RELB_049	55	3	25	0	132	1	0	0	4	0	17	basal	a	0	53	0,11	2	0	0	
RELB_050	55	3	22	0	132	1	1	1	4	0	15	luminal A	e	1	65	0,31	2	0	0	
RELB_051	49	2	10	0	132	0	1	0	3	3	15	luminal B	a	0	39	0,06	1	0	0	
RELB_052	71	3	30	0	132	1	0	0	4	3	23	Her2-enriched	b	1	65	0,16	2	1	2	Ex7 c.743 G>A R248Q
RELB_053	70	2	25	1	33	0	1	0	3	0	6	luminal A	f	0	101	0,45	3	0	0	
RELB_054	65	2	20	0	132	1	1	1	4	0	12	luminal A	e	0	60	0,61	2	0	0	
RELB_055	70	2	23	0	132	1	1	1	4	0	11	luminal A	e	0	46	0,34	2	0	0	
RELB_056	52	1	8	0	132	0	1	1	2	0	2	luminal A	d	4	68	0,18	2	0	0	
RELB_057	60	1	10	0	132	0	1	1	2	0	5	luminal A	e	0	39	0,16	1	0	0	
RELB_058	41	2	11	1	112	0	1	0	3	0	3	luminal A	d	0	59	0,19	2	0	0	
RELB_059	66	3	22	1	75	1	1	0	4	0	45	Luminal B	c	1	65	0,45	3	1	0	
RELB_060	41	3	15	0	132	0	1	0	3	0	6	luminal A	a	4	58	0,41	2	1	2	Ex7 c.703 A>G N235D
RELB_061	71	2	15	0	132	1	1	1	3	0	10	luminal A	c	10	47	0,22	2	0	0	
RELB_062	64	2	12	1	62	1	1	1	4	0	5	luminal A	b	0	50	0,22	2	0	0	
RELB_063	54	1	22	1	101	0	1	1	3	0	4	luminal A	b	0	43	0,22	2	0	0	
RELB_064	41	2	14	0	132	0	1	1	3	0	3	luminal A	e	0	46	0,10	2	0	0	
RELB_065	59	3	24	0	132	0	0	0	3	0	51	basal	a	0	78	0,24	2	1	0	
RELB_066	70	2	23	1	13	0	1	0	3	0	26	Luminal B	f	17	115	0,47	3	0	0	
RELB_067	44	2	20	0	132	0	1	1	2	3	23	luminal B	a	1	56	0,14	2	0	0	
RELB_068	62	2	20	0	132	1	1	1	3	0	21	Luminal B	a	10	59	0,22	2	0	0	
RELB_069	65	1	22	0	132	0	0	0	3	0	2	basal	a	0	49	0,19	2	0	0	
RELB_070	76	2	25	0	132	0	1	1	3	0	5	luminal A	b	2	56	0,18	2	0	0	
RELB_071	58	2	12	0	132	0	1	1	2	0	4	luminal A	e	0	57	0,22	2	0	0	
RELB_072	30	2	9	0	132	0	0	0	3	3	30	Her2-enriched	a	0	19	0,00	1	0	0	
RELB_073	52	1	20	0	132	1	1	1	3	0	0	luminal A	b	0	32	0,09	1	0	0	
RELB_074	68	2	18	0	132	1	1	1	3	0	15	luminal A	e	0	41	0,41	1	0	0	
RELB_075	77	2	17	1	95	0	1	1	3	0	11	luminal A	e	1	76	0,53	3	0	0	
RELB_076	56	2	20	0	132	0	1	1	2	0	4	luminal A	b	8	65	0,32	2	0	0	
RELB_077	75	2	20	0	132	0	1	0	3	0	0	luminal A	a	2	21	0,02	1	0	0	
RELB_078	46	3	20	1	16	1	1	1	3	0	62	Luminal B	c	0	77	0,43	3	0	1	Ex6 c.637 C>T R213X
RELB_079	44	2	15	0	132	0	1	1	3	3	21	luminal B	d	7	66	0,22	2	1	2	Ex7 c.711 G>A M237I
RELB_080	48	3	15	0	132	1	1	1	3	3	45	luminal B	e	10	82	0,27	2	1	2	Ex6 c.643 A>G S215G
RELB_081	61	2	18	1	108	1	1	1	3	0	3	luminal A	e	0	55	0,36	2	0	0	
RELB_082	65	3	25	0	132	0	0	0	3	3	18	Her2-enriched	a	4	101	0,26	2	1	2	Ex8 c.797 G>A G266E
RELB_083	68	2	23	0	132	0	1	1	3	0	15	luminal A	b	0	39	0,07	1	0	0	
RELB_084	57	2	18	0	132	1	1	1	3	0	8	luminal A	b	0	37	0,35	1	0	0	
RELB_085	65	2	20	0	132	0	1	1	2	0	20	luminal A	d	2	56	0,28	2	0	0	
RELB_086	65	2	23	1	68	1	1	1	4	0	11	luminal A	c	1	48	0,21	2	0	0	
RELB_087	42	3	20	0	132	1	0	0	3	0	60	basal	a	0	72	0,33	2	1	1	Ex10: c.1002_1008del7 R335X8
RELB_088	59	1	12	0	132	1	1	0	3	1	6	luminal A	f	0	88	0,58	3	0	0	
RELB_089	73	2	15	1	23	0	1	1	2	0	12	luminal A	b	0	31	0,08	1	0	0	
RELB_090	62	2	15	0	132	1	1	1	3	0	8	luminal A	d	0	52	0,32	2	0	0	

Supplementary table 1: samples annotations

**ID** (a)	**age**<br>(years)	**SBR**<br>Scarff, Bloom and Richardson grade	**tumor size**<br>(mm)	**relapse**<br>0 = no<br>1 = yes	**delay**<br>(months)	**pN histo**<br>Lymph node invasion<br>0 = negative<br>1 = positive	**ER status (IHC[b])**<br>ER: estrogen receptor<br>0: <10% cells<br>1: >=10% cells	**PR status (IHC)**<br>PR: progesteron receptor<br>0: <10% cells<br>1: >=10% cells	**NPI (group)**<br>1=excellent<br>2=good<br>3=moderate<br>4 = poor	**Her2 status (IHC)**<br>0: absence of signal<br>1-2-3: expression in at least 10% of cells with signal intensity of 1, 2 or 3	**mib-1 status (IHC)**<br>% of tumor cells expressing mib-1	**IHC phenotype**<br>Luminal A: HR =1, Her2 <3 and mib1 <20<br>Luninal B: HR=1, Her2<3 and mib1 >=20<br>Hrt2-enriched: Her2 =3<br>basal:ER and PR <10% ; Her2 <3; pcad or egfr or ck5/6 or vimentin: pos<br>nd:unclassified	**array-CGH cluster**	**number of Amplicons**	**Number of altered regions**<br>N	**Overal level of genomic alterations**<br>A	**G2I**<br>G2I-1=A<0,4876 and N<42,3638<br>G2I-3=A>0,3546 and N>65,0786	**p53 status (IHC)**<br>0: absence of signal<br>1: signal in at least 10% of tumor cells	**TP53 mutation**<br>0 : absence of mutation.<br>1: frameshift or nonsense mut.<br>2: misense mut.<br>nd:unclassified	**description of TP53 mutation**
RELB_091	63	2	14	0	132	0	1	1	3	0	6	luminal A	c	1	35	0,27	1	0	0	
RELB_092	62	2	12	0	132	0	1	1	3	0	15	luminal A	d	0	56	0,38	2	1	2	Ex8 c.844 C>T R282W
RELB_093	47	2	12	0	132	1	1	1	3	0	20	luminal A	a	0	47	0,35	2	0	1	Ex4 c.106_124del19 P36X1
RELB_094	46	2	25	0	132	1	1	1	3	0	16	luminal A	e	0	63	0,51	2	0	0	
RELB_095	47	1	20	0	132	0	1	1	2	0	8	luminal A	e	0	41	0,21	1	0	0	
RELB_096	45	2	20	1	89	0	1	1	3	0	8	luminal A	b	0	52	0,31	2	0	1	Ex4 c.227_279del53 A76fsX54
RELB_097	44	3	18	0	132	1	1	1	3	0	8	luminal A	c	0	29	0,25	1	0	0	
RELB_098	51	3	20	0	132	0	0	0	3	3	15	Her2-enriched	a	4	69	0,31	2	0	1	Ex5 c.546 C>A C182X
RELB_099	42	3	20	1	15	0	1	1	3	0	13	luminal A	e	0	82	0,47	3	1	2	Ex7 c.747 G>C R249S
RELB_100	70	3	23	1	8	0	0	0	3	0	60	Basal	f	9	106	0,71	3	0	1	Ex6 c.615 T>C Y205X
RELB_101	68	2	22	1	110	1	1	1	3	0	5	luminal A	c	0	64	0,29	2	0	0	
RELB_102	56	2	16	1	63	0	1	1	2	0	11	luminal A	b	0	90	0,19	2	0	0	
RELB_103	44	2	30	1	21	1	1	1	4	0	9	luminal A	e	1	88	0,33	2	0	0	
RELB_104	54	1	38	0	132	0	1	1	3	0	5	luminal A	b	0	46	0,28	2	1	2	Ex4 c.370 T>C C124R
RELB_105	73	3	25	0	132	0	1	1	3	2	28	Luminal B	a	2	56	0,26	2	0	0	
RELB_106	33	3	20	1	24	0	1	1	3	0	22	Luminal B	f	2	123	0,52	3	1	2	Ex5 c.376 T>A Y126N
RELB_107	46	1	20	0	132	0	1	1	2	0	7	luminal A	d	0	49	0,21	2	0	0	
RELB_108	66	3	25	1	11	1	0	0	4	3	12	Her2-enriched	a	2	76	0,26	2	1	0	
RELB_109	34	2	13	0	132	1	1	1	3	3	15	luminal B	e	6	65	0,24	2	0	0	
RELB_110	45	3	10	0	132	0	1	0	3	0	23	Luminal B	c	6	79	0,32	2	0	1	Ex4 c.375delG T125fsX45
RELB_111	48	2	13	0	132	0	1	1	3	0	22	Luminal B	c	2	44	0,27	2	0	0	
RELB_112	59	2	25	1	108	1	1	1	4	0	11	luminal A	e	0	45	0,19	2	0	0	
RELB_113	49	3	30	0	132	1	1	1	4	0	35	Luminal B	a	3	43	0,20	2	1	2	Ex7 c.712 T>C C238R
RELB_114	48	3	15	0	132	1	1	1	3	3	35	luminal B	d	3	73	0,26	2	0	1	Ex4 c.210insC
RELB_115	43	2	8	1	132	0	1	1	2	0	30	Luminal B	c	3	45	0,24	2	0	0	
RELB_116	66	3	30	0	132	1	1	1	4	0	21	Luminal B	b	0	55	0,09	2	0	0	
RELB_117	59	2	30	0	132	1	1	1	3	0	7	luminal A	b	0	33	0,17	1	0	0	
RELB_118	69	2	13	0	132	0	1	1	2	0	13	luminal A	a	0	40	0,15	1	0	0	
RELB_119	36	1	10	0	132	0	1	1	2	0	5	luminal A	b	0	49	0,15	2	1	2	Ex10 c.1025 G>C R342P
RELB_120	40	2	23	0	132	0	1	1	3	0	4	luminal A	b	0	56	0,17	2	0	0	
RELB_121	60	1	8	1	95	0	1	1	2	0	7	luminal A	f	1	95	0,62	3	0	0	
RELB_122	52	2	18	0	132	0	1	1	2	0	10	luminal A	a	1	70	0,44	3	0	0	
RELB_123	72	2	18	0	132	0	1	1	2	0	11	luminal A	a	0	33	0,12	1	0	0	
RELB_124	37	2	12	1	88	0	1	1	2	0	13	luminal A	c	1	54	0,19	2	0	0	
RELB_125	52	3	20	1	17	1	0	0	3	3	23	Her2-enriched	d	3	53	0,07	2	0	0	
RELB_126	69	3	23	1	21	0	1	1	3	0	10	luminal A	f	10	127	0,62	3	1	2	Ex8 839 G>T R280I
RELB_127	70	3	13	0	132	0	1	1	3	0	65	Luminal B	e	6	74	0,40	3	0	0	
RELB_128	42	1	20	0	132	1	1	1	3	0	5	luminal A	d	0	62	0,15	2	0	0	
RELB_129	55	2	15	1	124	1	1	1	3	0	18	luminal A	d	2	58	0,24	2	1	2	Ex5 c.473 G>T R158L
RELB_130	54	2	20	1	10	0	1	0	3	0	11	luminal A	f	7	76	0,42	3	1	0	
RELB_131	29	2	19	1	7	1	1	1	3	0	17	luminal A	a	5	66	0,29	2	1	2	Ex5 c.488 A>G Y163C
RELB_132	44	1	15	0	132	1	1	1	3	1	5	luminal A	f	0	97	0,41	3	1	2	Ex5 c.484 A>T I162F
RELB_133	43	3	25	0	132	1	1	1	3	0	26	Luminal B	e	7	56	0,31	2	1	0	
RELB_134	75	3	30	1	17	0	1	0	3	0	40	Luminal B	f	8	116	0,36	3	0	1	Ex4 c.297_304del8 Q100X46
RELB_135	65	2	30	0	132	1	1	0	4	1	17	luminal A	b	3	70	0,34	2	0	0	

(a) cases in grey indicate tumors with available transcriptomic data as well

(b) IHC: immunohistochemical data

Supplementary table 2: Incidence of the most frequent genomic gains and losses with respect to array CGH clusters

extent of copy number change [(a)]	chromosome band	genomic start position [(b)]	genomic end position[(b)]	status [(c)]	cluster a [(d)]	cluster b [(d)]	cluster c [(d)]	cluster d [(d)]	cluster e [(d)]	Gene ID or CNV	Gene description	regions with amplicons
chromosome arm	1p36.3-p34.3	849544	37432287	L					68			
	1q21-q44: +1q	142714793	245406794	G		97	76		69			
	7p22-p11.1	188237	57330946	G			73					
	7q11.2-q36	65773246	158457674	G			80					
	8p23-p12	139851	34482284	L				79				
	8q11.2-q24.3	48064196	146133391	G				99	73			
	16p13.3-p11.2	86672	33714444	G				71				
	16q12.2-q24.3	51142912	88643387	L		66		85	64			
	17p13-p11.2	343376	16463340	L				69				
chromosome bands	1p21	103873135	104049562	L				65	71	CNV		
	1p12-p11	120102485	121114115	G		61				CNV		1p12
	4q35	185584237	185711000	G					63	IRF2	interferon regulatory factor 2.	
	5p14	28156567	28338015	G				62	69	-		
	5p14-p13	28714271	29957832	G					61	PGBD3P2	piggyBac transposable element derived 3 pseudogene 2	
	6q16	100062194	100133623	L					63	CCNC	Cyclin C	
	6q27	170751274	170928001	L				62		CNV		
	8p12-8q11.1	36653064	47198510	G				70		various including FGFR1<br>LETM2<br>WHSC1L1	fibroblaste growth factor receptor 1.<br>Leucine zipper-EF-hand containing transmembrane protein 2.<br>Wolf-Hirschhorn syndrome candidate 1-Like 1	8p12 a et b ; 8p12-p11.21
	11q23-q24	111814998	125748324	L					71	various		
	12p13	12118026	12281072	L			63	69	63	BCL2L14<br>LRP6	BCL2-like 14 (apoptosis facilitator)<br>low density lipoprotein receptor-related protein 6	
	12q13	47263899	47441000	G				85		SNORA34;2A;2B<br>CCNT1	small nucleolar RNA, H/ACA box 34/2A/2B<br>Cycline T1.	12q13.11-13.12
	12q15	68555132	68606932	G				62		-		12q14.3-15
	12q21	83977453	84060921	G					63	LRRIQ1	leucine-rich repeats and IQ motif containing 1	12q21.31
	13q34	112494932	113999977	L				62		GRK1<br>GAS1<br>CUL4A<br>LAMP1<br>GRTP1<br>ADPRHL1<br>DCUN1D2<br>TFDP1<br>MCF2L	Growth arrest specific gene1<br>growth arrest-specific 1<br>Culin 4A<br>lysosomal-associated membrane protein 1<br>growth hormone regulated TBC protein 1.<br>ADP-ribosylhydrolase like 1<br>defective in cullin neddylation 1, domain containing 2<br>transcription factor Dp-1<br>MCF2-transforming sequence-like protein	
	15q22	61968824	62119201	G				69		DAPK2	death-associated protein kinase 2	15q22.2-q24.1
	16p13.3	2779832	4641451	G				62	63	various		
	16q12.1	45874737	50094137	L		64		72	57	-		
	17p13	7420977	7542427	L				77		TP53		
	17p13	7564112	13199220	L	64			73		various (PIK3R6)		
	17q11.2	23926254	24057738	G				77	69	SDF2	stromal cell-derived factor 2	17q11.1-q11.2 ; 17q11.2
	17q12	29835217	29998785	L				62		TMEM132E	unknown function	
	22q11.22	21211077	21411364	L			63			CNV		
	Xp11.1	58201229	58374974	L	62	60	81	77	77	CNV		

(a) extent of region with copy number change detected in at least 60% of tumors in a cluster, involving either a chromosome arm or a chromosome band

(b) genomic start or end position on the March 2006 human reference sequence (NCBI Build 36.1/Hg18)

(c) genomic status: L: loss or G: gain

Supplementary table 3: description of the amplicons

chromosome band	genomic start position (b)	genomic end position (b)	genes	Description of genes	total number of tumors	number of G2I-3 tumors
1p31.1	75168912	75597275	LHX8	LIM homeobox 8: Members of the LIM homeobox gene family	1	1
1p12	119870149	120037535	ZNF697	zinc finger protein 697	1	0
1q31.1-q31.2	188885607	190865412	RGS18 /RGS1 RGS21	regulator of G-protein signaling 18/1/21	1	0
1q32.1	201346093	202047453	ADORA1 - MYBPH<br>CHI3L1- CHIT1<br>BTG2 - FMOD<br>PRELP-OPTC-ATP2B4<br>SNORA77 - LAX1	Adenosine A1 receptor-proline/arginine-rich end leucine-rich repeat protein - Myosin binding protein H<br>Chitinase 3-like 1 (cartilage glycoprotein-39) - chitinase 1 (chitotriosidase)<br>B-cell translocation gene 2: member of the BTG/Tob family - Fibromodulin<br>Proline/arginine-rich end leucine-rich repeat protein - Opticin - ATPase, Ca++ transporting, plasma membrane 4.<br>Small nucleolar RNA, H/ACA box 77 - Lymphocyte transmembrane adaptor 1	2	2
3q25.33-q26.1	160964752	163801017	SCHIP1 - IL12<br>IFT80 - SMC4<br>TRIM59 - ARL14<br>B3GALNT1 - NMD3	Schwannomin interacting protein 1 - intraflagellar transport 80 homolog (Chlamydomonas) - Interleukine 12<br>Intraflagellar transport 80 homolog (Chlamydomonas) - Structural maintenance of chromosomes 4<br>Tripartite motif-containing 59 or RING finger 1-like - ADP-ribosylation factor-like 14.<br>Beta-1,3-N-acetylgalactosaminyltransferase 1 (globoside blood group) - NMD3 homolog:	1	1
3q26.2-q26.31	171682715	173687438	EIF5A2<br>TNIK<br>PLD1 - FNDC3B	Eukaryotic translation initiation factor 5A2<br>TRAF2 and NCK interacting kinase Stress-activated serine/threonine kinase<br>Phospholipase D1, phosphatidylcholine-specific - Factor for adipocyte differentiation 104	1	0
4q13.3-q21.1	75062607	76647213	PF4 - PPBP<br>CXCL 3/2/5<br>MTHFD2L<br>EPGN - EREG<br>AREG - BTC	Platelet factor 4 (chemokine (C-X-C motif) ligand 4) - Pro-platelet basic protein (chemokine (C-X-C motif) ligand 7)<br>Chemokine (C-X-C motif) ligand 3 / 2 / 5<br>Methylenetetrahydrofolate dehydrogenase (NADP+ dependent) 2-like protein.<br>Epithelial mitogen homolog (mouse) - Epiregulin<br>Amphiregulin (schwannoma-derived growth factor)/Colorectum cell-derived growth factor 2, 3 - Betacellulin	2	2
4q22.1	90883958	91062845	SNCA - MMRN1	Synuclein, alpha (non A4 component of amyloid precursor) - Multimerin 1	1	1
5p13.2-p13.1	38484979	38621958	EGFLAM - LIFR	EGF-like, fibronectin type III and laminin G domains - leukemia inhibitory factor receptor alpha	1	1
5q23.3	130203672	130353918	-	-	7	2
5q32	146744398	146950721	DPYSL3	Dihydropyrimidinase-like 3	2	0
6p24.3	9358225	9645938	no known gene	-	1	0
6q14.1	76118239	76312627	FILIP1	Filamin A interacting protein 1	1	0
6q21	106587688	106737871	PRDM1	PR domain containing 1, with ZNF domain	3	0
6q25.1-q25.2	151052348	153696621	ESR1 - MYCT1 - SYNE1	Estrogen receptor 1 - Myc target 1 - Synaptic nuclear envelope protein 1	4	2
7p15.1	29729747	29911984	WIPF3	WAS/WASL interacting protein family, member 3	1	1
8p12 a	37693145	37847478	ERLIN2 - PROSC - BRF2<br>RAB11FIP1	ER lipid raft associated 2 - Proline synthetase co-transcribed homolog (bacterial) - B-related factor 2<br>RAB11 family interacting protein 1 (class I)	11	3
8p12 b	38300020	38523430	WHSC1L1 - LETM2<br>FGFR1	Wolf-Hirschhorn syndrome candidate 1-like 1 - Leucine zipper-EF-hand containing transmembrane protein 2<br>Fibroblast growth factor receptor 1	5	3
8p12-p11.21	38388454	42044320	FGFR1 - TACC1<br>PLEKHA2<br>HTRA4 - TM2D2<br>ADAM9/32/2/18/3A<br>INDOL1 - ZMAT4	Fibroblast growth factor receptor 1 - Transforming, acidic coiled-coil containing protein 1<br>Pleckstrin homology domain containing, family A (phosphoinositide binding specific) member 2<br>HtrA serine peptidase 4 - TM2 domain containing 2<br>ADAM metallopeptidase domain 9/32/2/18/3A - indoleamine-pyrrole 2,3 dioxygenase-like 1<br>Zinc finger, matrin type 4	10	2
8q12.1	55683872	58525613	PLAG1	pleiomorphic adenoma gene 1	2	1
8q12.1-q12.2	61251559	61910067	CA8 - RAB2 - CDH7	Carbonic anhydrase VIII - RAB2A, member RAS oncogene family - Cadherin 7, type 2	4	2
8q13.1	66269708	67850992	PDE7A - MYBL1	Phosphodiesterase 7A - Nucleotides v-myb myeloblastosis viral oncogene homolog (avian)-like 1	5	2
8q21.11	75821214	75921964	PI15	peptidase inhibitor 15	5	2
8q21.13	80408445	81648505	STMN2 - HEY1 - TPD52	Stathmin-like 2 - Hairy/enhancer-of-split related with YRPW motif 1 - tumor protein D52	4	2
8q22.1	94050592	97062466	CDH17 - RAD54B	cadherin 17, LI cadherin (liver-intestine) - RAD54 homolog B (S. cerevisiae)	6	3
8q22.3	102004873	102268102	YWHAZ	Tyrosine 3-monooxygenase/tryptophan 5-monooxygenase activation protein, zeta polypeptide	3	2
8q23.3-q24.11	116334757	118046959	TRPS1 - EIF3H - RAD21	Trichorhinophalangeal syndrome I - RAD21 homolog (S. pombe)	5	1
8q24.11-q24.12	119096485	120383226	EXT1 - SAMD12<br>TNFRSF11B - COLEC10	Exostoses (multiple) 1 - Sterile alpha motif domain containing 12<br>Tumor necrosis factor receptor superfamily, member 11b - Collectin sub-family member 10 (C-type lectin)	5	3
8q24.13	123818644	124908744	DERL1 - WDR67 - ZHX1<br>ATAD2-MIRN548D1 - FBOXO32<br>ANX13	Der1-like domain family, member 1 - WD repeat domain 67 - Zinc fingers and homeoboxes 1<br>ATPase family, AAA domain containing 2 - F-box only protein 32<br>Annexin A13	6	3
8q24.21	127636652	129086494	POU5F1 - MYC	POU class 3 homeobox 1 - V-myc myelocytomatosis viral oncogene homolog (avian)	6	3
8q24.23	138579417	138778374	no known gene	-	4	2

Supplementary table 3: description of the amplicons

chromosome band	genomic start position (b)	genomic end position (b)	genes	Description of genes	total number of tumors	number of G2I-3 tumors
10p12.31	22567924	22718252	COMD3 - BMI1 - SPAG6	COMM domain containing 3 - BMI1 polycomb ring finger oncogene - Sperm associated antigen 6	2	1
10q22.2-q22.3	79296990	80682870	DLG5 - RPS24 - POLR3A<br>ZMIZ1	Discs large (DLG) homolog 5 - Ribosomal protein S24 - Polymerase (RNA) III (DNA directed) polypeptide A, 155kDa<br>Zinc finger, MIZ-type containing 1	3	0
10q26.12-q26.13	123070217	123327360	FGFR2	Fibroblast growth factor receptor 2/BEK fibroblast growth factor receptor	2	2
11q13.2-q13.3	68992359	69494732	CCND1 - ORAOV1 - FGF19/4/3	Cycline D1 - Oral cancer overexpressed 1 - Fibroblast growth factor 19 / 4/3	18	8
11q13.4	74417463	74580541	ORO2AT4 - SLCO2B1	Olfactory receptor, family 2, subfamily AT, member 4 - Solute carrier organic anion transporter family, member 2B1	3	1
11q14.1 a	76964811	78581745	RSF1 - INTS4 - THRSP<br>KCTD14/21<br>NDUFC2<br>ALG8<br>USP35-GAB2<br>NARS2-ODZ4	Remodeling and spacing factor 1 - Integrator complex subunit 4 - Thyroid hormone responsive (SPOT14 homolog, rat)<br>Potassium channel tetramerisation domain containing 14/21<br>NADH dehydrogenase (ubiquinone) 1, subcomplex unknown, 2, 14.5kDaU<br>Asparagine-linked glycosylation 8 homolog (S. cerevisiae, alpha-1,3-glucosyltransferase)<br>Ubiquitin specific peptidase 35 - GRB2-associated binding protein 2 -<br>Asparaginyl-tRNA synthetase 2, mitochondrial (putative) - Odz, odd Oz/ten-m homolog 4 (Drosophila)	11	3
11q14.1 b	83623372	83798264	DLG2	Discs, large homolog 2, chapsyn-110 (Drosophila)	3	1
12q13.11-q13.12	47263899	47441000	SNORA34/2A/2B - CCNT1	Small nucleolar RNA, H/ACA box 34/box2A - Cycline T1	7	3
12q14.1	57120242	57335694	no known gene	-	5	1
12q14.3	63783753	63965033	WIF1-LEMD3	WNT inhibitory factor 1 - LEM domain containing 3	4	1
12q14.3-q15	65848908	68135237	IFNG - IL26/22 - MDM1<br>MDM2 - RAP1B - NUP107	Interferon, gamma - Interleukin 26/ 22 - Mdm1 nuclear protein homolog (mouse)<br>Mdm2 p53 binding protein homolog (mouse) - RAP1B, member of RAS oncogene family - Nucleoporin 107kDa	2	1
12q21.31	80805511	80952302	no known gene		3	1
12q23.3	102794652	102924148	HSP90B1 - TDG	Heat shock protein 90kDa beta (Grp94), member - Thymine-DNA glycosylase	2	1
12q24.21	114829821	114989740	MED13L	Mediator complex subunit 13-like	1	0
12q24.31-q24.32	124397339	126794741	no kwown gene		1	0
14q24.3	74759155	74937247	FOS	v-fos FBJ murine osteosarcoma viral oncogene homolog	4	1
15q21.1	45554908	45746131	SEMA6D	Sema domain, transmembrane domain (TM), and cytoplasmic domain, (semaphorin) 6D	4	0
15q22.2-q24.1	58718485	71072936	various		1	0
15q25.2-q26.1	79990533	91436501	various		1	1
15q26.2-q26.3	95572059	97894328	ARRDC4 - IGF1R	Arrestin domain containing 4 - Insulin-like growth factor 1 receptor	4	0
16q12.1	47239429	47397252	no known gene		2	1
17q11.1-11.2	22685650	23921094	KSR1 - LGALS9 - NOS2A<br>NLK - PPY2 - TNFAIP1<br>POLDIP2 - SEBOX - VTN	Kinase suppressor of ras 1 - Lectin, galactoside-binding, soluble, 9 - Nitric oxide synthase 2A (inducible, hepatocytes)<br>Nemo-like kinase<br>Pancreatic polypeptide 2 - Tumor necrosis factor, alpha-induced protein 1 (endothelial)<br>Polymerase (DNA-directed), delta interacting protein 2 - SEBOX homeobox - Vitronectin/Somatomedin-B	2	1
17q11.2	23926254	24057738	SPAG5 -BCOX - FDF2	Sperm-associated antigen 5 - Breast cancer overexpressed gene 1 protein - Stromal cell-derived factor 2	6	3
17q12 a	31786567	31974170	TBC1D3G - ZNHIT3_MYO19	TBC1 domain family, member 3G - Zinc finger, HIT type 3 - Myosin 19	3	2
17q12 b	32855270	32991115	TADA2L - DUSP14<br>AP1GBP1	Transcriptional adaptor 2 (ADA2 homolog, yeast)-like - Dual specificity phosphatase 14/MAP kinase phosphatase 6<br>AP1 gamma subunit binding protein 1	4	1
17q12 c	32964235	33147865	AP1GBP1 - DDX52 - HNF1B	AP1 gamma subunit binding protein 1 - DEAD (Asp-Glu-Ala-Asp) box polypeptide 52/ATP-dependent RNA helicase - HNF1	3	2
17q12 d	34054485	34434812	MLLT6<br>PCGF2 - PSMB3<br>PIP4K2B - CCDC49 - RPL23<br>SNORA21 - LASP1	Myeloid/lymphoid or mixed-lineage leukemia (trithorax homolog,Drosophila); translocated to, 6<br>Polycomb group ring finger 2 - Proteasome (prosome, macropain) subunit, beta type, 3<br>Phosphatidylinositol-5-phosphate 4-kinase, type II, alpha - Coiled-coil domain containing 49 - Ribosomal protein L23<br>Small nucleolar RNA, H/ACA box 21 - LIM and SH3 protein 1	5	2
17q12 e	34987922	35394585	STARD3<br>TCAP - PNMT - PERLD1 - ERBB2<br>GRB7 - IKZF3 - GSDMB	Metastatic lymph node protein 64/StAR-related lipid transfer (START) domain containing 3<br>Titin-cap (telethonin) - Phenylethanolamine N-methyltransferase - Per1-like domain containing 1<br>Growth factor receptor-bound protein 7 - IKAROS family zinc finger 3 (Aiolos) - Gasdermin B	17	4
17q21.1-q21.2	35467801	35669669	NR1D1 - MSL-1<br>CACS3<br>RAPGEFL1 - WIPF2	Nuclear receptor subfamily 1, group D, member 1 - Male-specific lethal-1 homolog<br>Cancer susceptibility candidate 3/Metastatic lymph node protein 51<br>Rap guanine nucleotide exchange factor (GEF)-like 1 - WAS/WASL interacting protein family, member 2	8	2
17q21.31	37446175	39040025	STAT5B/5A/3 - HSD17B<br>TUBG1 - WNK4 - BECN1<br>AOC3 - BRCA1<br>NBR1	Signal transducer and activator of transcription 5B/5A/3 - Hydroxysteroid (17-beta) dehydrogenase 1<br>Tubulin, gamma 1 - WNK lysine deficient protein kinase 4 - Beclin 1<br>Autophagy related amine oxidase, copper containing 3 (vascular adhesion protein 1)<br>Neighbour of BRCA1 gene 1/ovarian carcinoma antigen CA125	8	2
17q21.33	44940639	45156078	NGFR - NXPH3 - SPOP	Nerve growth factor receptor (TNFR superfamily, member 16) - Neurexophilin 3 - Speckle-type POZ protein-like	8	4
17q22	53670992	538454449	LPO-MPO-BZRAP1-RNF43	lactoperoxidase - myeloperoxidase -benzodiazapine receptor (peripheral) associated protein 1 - ring finger protein 43	6	3
17q23.2-q23.3	58263128	58922997	MIRN633-TANC2	tetratricopeptide repeat, ankyrin repeat and coiled-coil containing 2	6	4

Supplementary table 3: description of the amplicons

chromosome band	genomic start position (b)	genomic end position (b)	genes	Description of genes	total number of tumors	number of G2I-3 tumors
17q23.3	59113749	59554357	MAP3K3 - LYK5 - CCDC47<br>DDX42<br>SMACD2<br>CSH1/2<br>GH1-ICAM2	Mitogen-activated protein kinase kinase kinase 3 - Protein kinase LYK5 - Coiled-coil domain containing 47<br>DEAD (Asp-Glu-Ala-Asp) box polypeptide 42<br>SWI/SNF related, matrix associated, actin dependent regulator of chromatin, subfamily d, member 2<br>Chorionic somatomammotropin hormone 1 (placental lactogen) /2 - Growth hormone 1<br>Intercellular adhesion molecule 2	6	3
17q24.1	60379640	61265765	GNA13 - RGS9 - AXIN2<br>CCDC46	Guanine nucleotide binding protein (G protein), alpha 13 - Regulator of G-protein signaling 9 - Axin 2 (conductin, axil)<br>Coiled-coil domain containing 46	2	1
17q24.2	63118104	63295003	PITPNC1<br>NOL1 - BTPF	Phosphatidylinositol transfer protein, cytoplasmic 1<br>Nucleolar protein 1, 120kDa/Proliferating-cell nucleolar antigen p120 - Bromodomain PHD finger transcription factor	4	2
17q25.1	70781219	71445677	GRB2 - LLGL2 - RECQL5<br>SAP30BP - ITGB4 - GALK1 - H3F3B<br>UNK - WBP2	Growth factor receptor-bound protein 2 - Lethal giant larvae homolog 2 (Drosophila) - RecQ protein-like 5<br>SAP30 binding protein -Integrin, beta 4 - Galactokinase 1 - H3 histone, family 3B<br>Unkempt homolog (Drosophila) - WW domain binding protein 2	3	2
18p11.31	3607103	4613737	DLGAP1 - GAPDHL11	Discs, large (Drosophila) homolog-associated protein 1 - Glyceraldehyde-3-phosphate dehydrogenase-like 11	1	1
18p11.23	8256377	8468885	PTRM	Protein tyrosine phosphatase, receptor type, M	1	1
18p11.21	12818154	13803216	PTPN2 - SEH1L - CEP192	Protein tyrosine phosphatase, non-receptor type 2 - SEH1-like (S. cerevisiae) - Centrosomal protein 192kDa	1	1
18p11.21-q22.1	58880584	64298616	BCL2- VPS4B<br>SERPINB4/5/12/13/3/11/7/8/2/10<br>HSMD<br>CDH7- CDH19<br>DSEL	B-cell CLL/lymphoma 2 - Vacuolar protein sorting 4 homolog B (S. cerevisiae)<br>Serpin peptidase inhibitor, clade B (ovalbumin), member 4/5/12/13/3/11/7/8/2/10<br>Histocompatibility (minor) serpin domain containing - Cadherin 7, type 2 cadherin 19, type 2<br>Dermatan sulfate epimerase-like	1	1
19p13.2	11023371	11151169	SMARCA4<br>LDLR<br>SPC24	SWI/SNF related, matrix associated, actin dependent regulator of chromatin, subfamily a, member 4<br>Low density lipoprotein receptor (familial hypercholesterolemia)<br>SPC24, NDC80 kinetochore complex component, homolog (S. cerevisiae)	4	0
19q13.32	41164341	41314590	ALKBH6 - CLIP3<br>THAP8 - WDR62 - POLR2I<br>TBCB	AlkB, alkylation repair homolog 6 (E. coli) - CAP-GLY domain containing linker protein 3<br>THAP domain containing 8 - WD repeat domain 62 - Polymerase (RNA) II (DNA directed) polypeptide I, 14.5kDa<br>Tubulin folding cofactor B	1	1
19q13.12-q13.31	48848178	49021209	PLAUR - IRGC<br>KCNN4<br>LYPD5	Plasminogen activator, urokinase receptor - Immunity-related GTPase family, cinema<br>Potassium intermediate/small conductance calcium-activated channel, subfamily N, member 4<br>LY6/PLAUR domain containing 5	1	1
20q11.21	30031501	30226018	XKR7 - HCK<br>TM9SF4	XK, Kell blood group complex subunit-related family, member 7 - Hemopoietic cell kinase<br>Transmembrane 9 superfamily protein member 4	6	4
20q12	40030026	40190025	PTPRT	Protein tyrosine phosphatase, receptor type, T	2	2
20q13.12a	41709495	41887619	MYBL2 - GTSF1L	Target of myb1 (chicken)/TOM1 - Gametocyte specific factor 1-like	3	2
20q13.12b	45130950	45339408	EYA2 - ZMYND8	Eyes absent homolog 2 (Drosophila) - Zinc finger, MYND-type containing 8	4	3
20q13.12-q13.13	45646762	45864691	NCOA3 - SULF2	Nuclear receptor coactivator 3/Amplified in breast cancer-1 protein - Sulfatase 2	4	3
20q13.13	48173498	48408361	CEBPB	CCAAT/enhancer binding protein (C/EBP), beta	3	2
20q13.2	50383552	52257683	TSHZ2 - ZNF217 - BCAS1	Teashirt zinc finger homeobox 2 - Zinc finger protein 217 - Breast carcinoma amplified sequence 1	7	3
20q13.31-q13.32	55139993	55922598	BMP7 -S PO11<br>PMEPA1	Bone morphogenetic protein 7 - SPO11 meiotic protein covalently bound to DSB homolog (S. cerevisiae)<br>Prostate transmembrane protein, androgen induced 1	7	4
20q13.32-q13.33	59571817	60208710	CDH4<br>TAF4	Cadherin 4, type 1, R-cadherin (retinal)<br>TAF4 RNA polymerase II, TATA box binding protein (TBP)-associated factor, 135kDa	1	1
21q21.2-q21.3	25400779	26168713	MIRN155-MRPL39<br>JAM2 - FOXP2	Mitochondrial ribosomal protein L39<br>Junctional adhesion molecule 2 - Forkhead box P2	1	0
21q22.13-q22.3	37360933	45142738	various		1	0
22q12.2	28266920	28417086	THOC5 - NIPSNAP1 - NF2	THO complex 5 - Nipsnap homolog 1(C. elegans) - Neurofibromin 2 (merlin)	3	2
22q13.32	47762144	47962891	no known gene	-	2	2
Xp22.2	15198599	15677747	ASB9/11 - PIGA<br>FIGF - PIR<br>BMX - ACE2	Ankyrin repeat and SOCS box-containing 9/11 - Phosphatidylinositol glycan anchor biosynthesis, class A<br>c-fos induced growth factor (vascular endothelial growth factor D) - Pirin (iron-binding nuclear protein)<br>BMX non-receptor tyrosine kinase - Angiotensin I converting enzyme (peptidyl-dipeptidase A) 2	1	1
Xq22.3-q27.3	107156265	145782456	various		1	1

(a) genomic start or end position on the March 2006 human reference sequence (NCBI Build 36.1 / Hg18)

Supplementary table 4: recurrently lost and gained genomic regions in G2I-3 tumours

chromosomal band	*start*	*end*	*GNL status*	*% L or G*	*s.d.*	*p-value*	*genes*
1p31	69433766	83736513	L	0,65	0,05	1,30802E-06	multiple
1p21	96364079	106958243	L	0,65	0,03	2,50454E-06	multiple - CDC14A
2p12(1cl)	77381801	77582691	L	0,50		3,48938E-06	
2q14.1	115940769	117433903	L	0,48	0,04	1,73344E-05	DPP10
2q14.3	127490577	127839679	L	0,50	0,00	8,16679E-05	
2q21-2q22	135635338	145268118	L	0,58	0,04	4,40923E-06	LRP1B
2q23-q24	153986952	155861786	L	0,52	0,02	6,18625E-07	KCNJ3
3p24	19033561	22313155	L	0,54	0,00	5,56944E-05	RAB5A - ZNF385D
3p12 (1cl)	75883399	75997474	L	0,50		0,000170757	
4p15.3-p14	17884311	36984301	L	0,61	0,07	0,000275663	CENTD1
4p13	42860555	45776198	L	0,55	0,02	0,000139081	GUF1 - GNPDA2
4q22-q25	90883958	109959159	L	0,54	0,03	1,65081E-06	CENPE - MTTP - SMARCAD1 - BDH2
4q28	132462917	137761298	L	0,53	0,03	6,73275E-07	PCDH10
4q31.3-q32	155803832	169783309	L	0,56	0,04	1,04497E-06	MAP9 -RAPGEF2 - NAF1 - KLHL2
5q14(1cl)	85444213	85587083	L	0,50		1,52201E-05	
5q21-q22	109440971	109844086	L	0,50	0,00	5,82345E-06	
5q23	119844084	120486136	L	0,52	0,02	0,000761202	
6p12(1cl)	48122339	48296486	L	0,50		6,31087E-07	
6q11.1-q14	62399682	83789522	L	0,59	0,07	0,00071993	TTK - MTO1 - COX7A2 - HMGN3 - FAM135A - CD109 - SOD25 - WTAP
6q16	93870393	98804093	L	0,64	0,04	0,001860349	KIAA0776 - MANEA
6q21-q22	112903193	129293585	L	0,61	0,04	0,000979954	ASF1A - …
7q21	83717661	83923151	L	0,52	0,02	6,31087E-07	
7q31-q32	118436901	126006508	L	0,50	0,00	1,25637E-05	ING3
9p24	2280507	8410998	L	0,59	0,02	8,45989E-05	multiple
9p23-p22	10288475	14848928	L	0,72	0,10	6,9881E-06	multiple
9p21	21733188	31833258	L	0,68	0,07	1,77424E-05	CDKN2A
9p11.1	44289153	45919960	L	0,62	0,02	2,96673E-07	
9q12	64136764	66762409	L	0,61	0,00	9,81087E-06	
10q21	53584260	67289025	L	0,58	0,03	5,4878E-08	PCDH15
11p14	22690875	29117657	L	0,51	0,01	2,02512E-06	SVIP
11p12	39034573	41333167	L	0,50	0,00	1,8033E-07	
11q14-q22	87924476	107886043	L	0,74	0,09	5,88923E-05	CASP - ATM
12q14-q21	61345783	70475338	G	0,52	0,02	0,000156027	multiple - WIF1 - MDM2
13q21	59407380	71304898	L	0,70	0,02	0,000426044	multiple
13q31	78745923	93116597	L	0,66	0,04	9,42409E-05	multiple
14q12	27635797	28334953	L	0,50	0,00	1,70201E-05	FOXG1
14q31-32	83206225	93819353	L	0,60	0,06	8,66485E-05	ATXN3 - SERPINA1
15q14	33551572	36541031	L	0,60	0,02	4,48E-06	ATPBD4
15q21	44046023	52245884	L	0,57	0,02	4,50165E-06	MYO5A
15q26 (1cl)	95572059	95680324	L	0,54		1,07007E-07	
16p13.3 (1 cl)	2779832	2953509	G	0,68		0,000671392	LYRM1 - CLN3
17q11.2	23823754	24040368	G	0,52	0,02	0,000165847	SDF2
17q25 (1 cl)	74580696	74795375	G	0,57		0,000322211	EIF4A3
18p11.2	14979979	15367712	L	0,63	0,02	5,94399E-06	ANKRD30B
18q12	23277550	38729325	L	0,58	0,06	0,000492029	ZNF397 - INO80C
18q22	61064094	68885157	L	0,68	0,04	7,59901E-05	SOCS6
20p13-p11.2	18098727	49736686	G	0,57	0,04	7,71466E-05	multiple
20q11.2-q13.3	29653482	62160261	G	0,75	0,05	1,30752E-05	multiple
21q11.2-q21	13602308	28419968	L	0,58	0,04	1,51232E-05	USP25
Xp22.2	10133499	15392707	L	0,52	0,03	6,00708E-05	multiple
Xp22.1	20566912	21313933	L	0,55	0,05	4,54962E-05	
Xp21-p11.4	31749009	38158504	L	0,53	0,01	0,000104597	multiple
Xq21	79544292	97276033	L	0,51	0,01	1,30823E-05	multiple
Xq25	120579120	126778500	L	0,56	0,03	2,20257E-06	XIAP
Xq27	141740494	145782456	L	0,50	0,00	8,16679E-05	SLITRK4 SPANX1 - SLITRK2

Supplementary table 5: list of probe sets defining the transcritomic signature of the G2I-3 tumours and mapping on array-CGH BAC clones

Expression data of the 46 breast tumors										Genomic data of the the 46 breast tumors							
probe set	***entrez Gene Symbol***	***chromosome***	***chromosome band***	***genomic start position***	***genomic end position***	***kegg***	***genes Cluster<br>a=genes under expressed in G2I-3<br>b=genes overexpressed in G2I-3***	***p-value***	***FC G2I-1 or -2 versus G2I-3***	***BAC clone name***	***BAC clone start position***	***BAC clone end position***	***BAC clone status***	***% of G2I-1 or-2 tumors with genomic Gain n32***	***% of G2I-1 or-2 tumors with genomic Loss n32***	***% of G2I-3 tumors with genomic Gain n14***	***% of G2I-3 tumors with genomic Loss n14***
203879_at	**PIK3CD**	1	p36.22	9634389	9711561	Apoptosis // B cell receptor signaling pathway // Chronic myeloid leukemia // Colorectal cancer // Endometrial cancer // ErbB signaling pathway // Fc epsilon RI signaling pathway // Focal adhesion // Glioma // Inositol phosphate metabolism // Insulin signaling pathway // Jak-STAT signaling pathway // Leukocyte transendothelial migration // Melanoma // Natural killer cell mediated cytotoxicity // Non-small cell lung cancer // Pancreatic cancer // Phosphatidylinositol signaling system // Prostate cancer // Regulation of actin cytoskeleton // Renal cell carcinoma // Small cell lung cancer // T cell receptor signaling pathway // Toll-like receptor signaling pathway // Type II diabetes mellitus // VEGF signaling pathway // mTOR signaling pathway	a	3,3E-03	0,49	FE0ADELA7ZE01	9215216	9384650	OUT.s	0,00	1,00	0,00	0,00
219058_x_at	TINAGL1	1	p35.2	31814746	31825872		a	1,8E-04	0,44	FE0ADELA4ZB01	31540381	31685941	OUT.s	0,09	0,13	0,21	0,36
1554140_at	WDR78	1	p31.3	67075873	67163057		a	2,0E-05	0,51	FE0BPADA4ZA03	66606377	66787022	OUT.s	0,06	0,09	0,21	0,29
207415_at	PLA2R1	2	q24.2	160511109	160627367		a	2,1E-03	0,13	FE0DBACA1ZF03	160370047	160524475	IN.e	0,16	0,09	0,07	0,29
228721_at	MAP3K4	3	p22.1	42952871	42959288	GnRH signaling pathway // MAPK signaling pathway	a	9,9E-05	0,72	FE0DBACA18ZD06	42212492	42410228	OUT.s	0,03	0,31	0,00	0,21
203854_at	CFI	4	q25	110881300	110942590	Complement and coagulation cascades	a	5,0E-04	0,86	FE0DBACA10ZB09	109755921	109959159	OUT.s	0,09	0,06	0,14	0,50
1555564_a_at	CFI	4	q25	110881302	110942645	Complement and coagulation cascades	a	7,9E-04	0,93	FE0DBACA10ZB09	109755921	109959159	OUT.s	0,09	0,06	0,14	0,50
203480_s_at	OTUD4	4	q31.22	146274252	146279814		a	5,0E-03	0,25	FE0DBACA17ZB08	145266257	145366827	OUT.s	0,13	0,03	0,14	0,36
223734_at	OSAP	4	q31.1	140406775	140420829		a	2,0E-04	0,30	FE0BPADA10ZG07	139770886	139939393	OUT.s	0,09	0,03	0,14	0,43
227235_at	NA	4	q32.1	156876294	156877661	NA	a	2,3E-04	0,95	FE0BPADA12ZC01	156675950	156846238	OUT.s	0,13	0,06	0,14	0,43
239580_at	NA	4	q32.1	156875444	156876254	NA	a	2,7E-04	0,71	FE0BPADA12ZC01	156675950	156846238	OUT.s	0,13	0,06	0,14	0,43
229427_at	NA	5	p15.2	9090796	9091792	NA	a	1,8E-04	0,79	FE0DBACA4ZD03	7387106	7553242	OUT.s	0,00	0,00	0,00	0,00
213169_at	NA	5	p15.2	9088141	9090509	NA	a	7,0E-04	0,81	FE0DBACA4ZD03	7387106	7553242	OUT.s	0,00	0,00	0,00	0,00
235657_at	CFI	5	q35.3	178886647	178887176	Complement and coagulation cascades	a	1,4E-04	0,65	FE0BPADA7ZH06	177813565	177977675	OUT.s	0,16	0,03	0,29	0,00
231867_at	ODZ2	5	q35.1	167558645	167623739		a	5,4E-04	1,17	FE0BPADA9ZA10	167258780	167396907	OUT.s	0,00	0,00	0,00	0,00
227475_at	FOXQ1	6	p25.3	1259110	1259993		a	4,5E-03	0,81	FE0DBACA24ZC08	230896	357473	OUT.s	0,09	0,13	0,21	0,29
216841_s_at	**SOD2**	6	q25.3	160023301	160034249		a	1,5E-03	0,70	FE0BPADA6ZA11	159028199	159243884	OUT.s	0,06	0,19	0,07	0,50
222942_s_at	**TIAM2**	6	q25.2	155511030	155623443	Regulation of actin cytoskeleton	a	1,1E-04	0,64	FE0DBACA5ZH10	154093732	154273929	OUT.s	0,09	0,28	0,00	0,64
203137_at	**WTAP**	6	q25.3	160068609	160090437		a	7,3E-04	0,62	FE0BPADA6ZA11	159028199	159243884	OUT.s	0,06	0,19	0,07	0,50
226545_at	**CD109**	6	q13	74592986	74594757		a	2,1E-04	1,22	FE0BPADA12ZF07	74097866	74274274	OUT.s	0,03	0,16	0,07	0,57
229900_at	**CD109**	6	q13	74577491	74590546		a	4,5E-04	0,77	FE0BPADA12ZF07	74097866	74274274	OUT.s	0,03	0,16	0,07	0,57
239719_at	**CD109**	6	q13	74590842	74591517		a	3,8E-04	0,62	FE0BPADA12ZF07	74097866	74274274	OUT.s	0,03	0,16	0,07	0,57
223946_at	**MED23**	6	q23.2	131936798	131991038		a	4,5E-03	0,63	FE0ADELA14ZH04	131620520	131752055	OUT.s	0,16	0,25	0,14	0,57
204089_x_at	MAP3K4	6	q26	161332811	161458371	GnRH signaling pathway // MAPK signaling pathway	a	3,8E-04	0,47	FE0DBACA1ZE02	160956196	161050953	OUT.s	0,06	0,31	0,07	0,71
216199_s_at	MAP3K4	6	q26	161449900	161458407	GnRH signaling pathway // MAPK signaling pathway	a	1,2E-03	0,46	FE0DBACA1ZE02	160956196	161050953	OUT.s	0,06	0,31	0,07	0,71
203837_at	MAP3K5	6	q23.3	136919880	137155349	MAPK signaling pathway	a	4,5E-04	0,74	FE0ADELA13ZG07	136892735	137046976	IN.e	0,16	0,25	0,14	0,57
204214_s_at	RAB32	6	q24.3	146906693	146917655		a	7,3E-05	0,72	FE0BPADA7ZC01	146065431	146266182	OUT.s	0,16	0,25	0,07	0,64
208671_at	SERINC1	6	q22.31	122806450	122834635		a	2,6E-04	0,33	FE0DBACA19ZG07	122067674	122255127	OUT.s	0,13	0,28	0,00	0,69
209447_at	SYNE1	6	q25.1	152484607	152502990		a	3,3E-03	0,50	FE0APADA2ZD10	152175458	152384017	OUT.s	0,19	0,22	0,07	0,50
226794_at	STXBP5	6	q24.3	147716163	147749219		a	1,4E-03	0,41	FE0BPADA6ZH03	147132229	147329173	OUT.s	0,16	0,25	0,07	0,64
219532_at	ELOVL4	6	q14.1	80681254	80713862		a	4,9E-03	0,38	FE0BPADA11ZA03	80323115	80486324	OUT.s	0,03	0,22	0,00	0,79
225897_at	MARCKS	6	q22.1	114289317	114291339		a	1,7E-03	0,54	FE0ADELA15ZG02	114217356	114383683	IN.se	0,13	0,28	0,00	0,71
236528_at	UBE2J1	6	q15	90103680	90104242		a	2,5E-03	0,28	FE0DBACA4ZD11	89884589	90038559	OUT.s	0,00	0,25	0,00	0,64
225184_at	ARID1B	6	q25.3	157495933	157572088		a	4,9E-04	0,45	FE0DBACA19ZF08	155844695	156033918	OUT.s	0,13	0,25	0,00	0,64
222805_at	MANEA	6	q16.1	96132139	96164044		a	1,9E-03	0,61	FE0DBACA19ZC07	95061616	95166348	OUT.s	0,03	0,29	0,00	0,77
222391_at	TMEM30A	6	q14.1	76019362	76051221		a	2,8E-03	0,41	FE0BPADA12ZF07	74097866	74274274	OUT.s	0,03	0,16	0,07	0,57
240020_at	NA	6	q14.3	84733237	84733862	NA	a	4,7E-04	0,18	FE0BPADA11ZC06	83775905	83925750	OUT.s	0,06	0,22	0,00	0,79
230589_at	NA	6	q21	112027770	112030188	NA	a	1,8E-04	0,19	FE0DBACA19ZF07	111942077	112048236	IN.se	0,07	0,33	0,07	0,57
1564438_at	NA	6	q25.2	155251821	155253590	NA	a	2,5E-04	0,13	FE0DBACA5ZH10	154093732	154273929	OUT.s	0,09	0,28	0,00	0,64
235227_at	NA	6	q24.3	147752262	147753297	NA	a	4,3E-04	0,50	FE0BPADA6ZH03	147132229	147329173	OUT.s	0,16	0,25	0,07	0,64
205890_s_at	UBD	6	NA	780660	784762		a	3,2E-03	1,25	FE0DBACA24ZC08	230896	357473	OUT.s	0,09	0,13	0,21	0,29
205816_at	ITGB8	7	p15.3	20337270	20416944	Cell adhesion molecules (CAMs) // ECM-receptor interaction // Focal adhesion // Regulation of actin cytoskeleton	a	5,6E-05	0,54	FE0DBACA19ZD09	19960406	20080502	OUT.s	0,00	0,00	0,00	0,50
229912_at	SDK1	7	p22.2	4272018	4272565		a	1,2E-06	0,80	FE0ADELA13ZC03	3513001	3662566	OUT.s	0,22	0,00	0,14	0,07
229407_at	SDK1	7	p22.2	4273836	4275155		a	2,8E-03	0,41	FE0ADELA13ZC03	3513001	3662566	OUT.s	0,22	0,00	0,14	0,07
226189_at	NA	7	p15.3	20419960	20421907	NA	a	6,5E-04	1,82	FE0DBACA19ZD09	19960406	20080502	OUT.s	0,00	0,00	0,00	0,50
243526_at	WDR86	7	q36.1	150709142	150709438		a	5,4E-04	0,40	FE0DBACA20ZB03	150360814	150500398	OUT.s	0,19	0,00	0,29	0,29
209227_at	**TUSC3**	8	p22	15442102	15667225		a	7,0E-04	1,00	FE0DBACA12ZC02	15547187	15733616	IN.s	0,09	0,47	0,07	0,64
205667_at	**WRN**	8	p12	31010875	31150818		a	3,4E-03	0,42	FE0DBACA23ZB05	31055555	31208199	IN.s	0,09	0,47	0,07	0,64
1557347_at	**MCPH1**	8	p23.1	6488518	6490536		a	4,3E-03	0,35	FE0BPADA5ZF03	6182469	6364998	OUT.s	0,09	0,47	0,07	0,64
32541_at	PPP3CC	8	p21.3	22354549	22454600	Apoptosis // Axon guidance // B cell receptor signaling pathway // Calcium signaling pathway // Long-term potentiation // MAPK signaling pathway // Natural killer cell mediated cytotoxicity // T cell receptor signaling pathway // VEGF signaling pathway // Wnt signaling pathway	a	2,5E-03	0,40	FE0APADA1ZA02	22048391	22230663	OUT.s	0,09	0,47	0,07	0,64
223475_at	CRISPLD1	8	q21.11	76059666	76108087		a	6,5E-04	2,59	FE0BPADA5ZB11	76034751	76219867	IN.se	0,41	0,00	0,71	0,07
236453_at	SYNE1	9	p11.2	45454181	45454631		a	1,8E-04	0,52	FE0APADA2ZH09	44418232	44589317	OUT.s	0,06	0,09	0,07	0,50
219265_at	MOBKL2B	9	p21.2	27319128	27519779		a	1,3E-03	0,34	FE0DBACA12ZB08	27430391	27597320	IN.s	0,03	0,31	0,07	0,64

Supplementary table 5: list of probe sets defining the transcritomic signature of the G2I-3 tumours and mapping on array-CGH BAC clones

218992_at	C9orf46	9	p24.1	5347972	5427860		a	4,3E-03	0,55	FE0BPADA4ZG06	5009594	5181448	OUT.s	0,16	0,16	0,07	0,64
226844_at	MOBKL2B	9	p21.2	27315785	27316900		a	9,3E-05	0,93	FE0DBACA27ZD11	27163232	27323328	IN.se	0,03	0,31	0,07	0,64
219343_at	CDC37L1	9	p24.1	4669668	4696501		a	2,2E-03	0,55	FE0DBACA5ZB10	3277783	3446865	OUT.s	0,16	0,19	0,08	0,77
205818_at	**DBC1**	9	q33.1	120968728	121171522		a	4,3E-03	0,32	FE0DBACA3ZF08	121159848	121310135	IN.s	0,06	0,13	0,00	0,21
204457_s_at	GAS1	9	q21.33	88749097	88751924	Hedgehog signaling pathway	a	1,9E-03	0,77	FE0DBACA12ZH02	88305358	88478980	OUT.s	0,06	0,13	0,00	0,29
213083_at	SLC35D2	9	q22.32	98122808	98185775		a	2,8E-05	0,47	FE0ADELA8ZA12	97484626	97658608	OUT.s	0,13	0,09	0,07	0,21
240275_at	**ARMC3**	10	p12.2	23361824	23366517		a	1,5E-03	0,30	FE0DBACA5ZH09	23263838	23454971	IN.se	0,06	0,06	0,21	0,14
202565_s_at	SVIL	10	p11.23	29786282	30064736		a	1,9E-03	0,74	FE0DBACA20ZB10	29585210	29749487	OUT.s	0,00	0,00	0,50	0,00
221824_s_at	MARCH8	10	q11.21	45270039	45272252		a	2,1E-03	0,94	FE0DBACA11ZB04	44800643	44988426	OUT.s	0,03	0,09	0,07	0,29
232846_s_at	CDH23	10	q22.1	73120233	73137268		a	8,6E-04	0,25	FE0DBACA20ZE11	73028181	73138672	IN.se	0,00	0,09	0,07	0,29
204490_s_at	**CD44**	11	p13	35117311	35207597	ECM-receptor interaction // Hematopoietic cell lineage	a	2,0E-03	0,90	FE0DBACA10ZD02	33914327	34095862	OUT.s	0,16	0,06	0,14	0,07
212014_x_at	**CD44**	11	p13	35117303	35208819	ECM-receptor interaction // Hematopoietic cell lineage	a	2,8E-03	0,98	FE0DBACA10ZD02	33914327	34095862	OUT.s	0,16	0,06	0,14	0,07
232947_at	**DKK3**	11	p15.3	11949170	11951776		a	2,5E-04	0,18	FE0BPADA6ZF03	11757785	11956710	IN.se	0,09	0,19	0,14	0,21
230214_at	MRVI1	11	p15.4	10553882	10554448		a	4,8E-03	0,69	FE0APADA1ZD05	10526853	10691597	IN.se	0,06	0,19	0,07	0,07
206528_at	TRPC6	11	q22.1	100828632	100959461		a	3,2E-04	0,25	FE0BPADA8ZA08	99932478	100132546	OUT.s	0,00	0,00	0,00	0,50
207723_s_at	KLRC3	12	p13.2	10456179	10464461	Antigen processing and presentation // Natural killer cell mediated cytotoxicity	a	3,3E-04	0,40	FE0DBACA9ZF01	9623735	9799377	OUT.s	0,09	0,06	0,36	0,29
202177_at	GAS6	13	q34	113546912	113590396		a	9,6E-04	0,66	FE0DBACA10ZF04	112314989	112494932	OUT.s	0,03	0,34	0,14	0,29
1598_g_at	GAS6	13	q34	113546912	113590396		a	2,9E-03	0,45	FE0DBACA10ZF04	112314989	112494932	OUT.s	0,03	0,34	0,14	0,29
205880_at	**PRKD1**	14	q12	29115439	29466678		a	2,6E-03	0,52	FE0DBACA8ZF03	29232717	29398694	IN.se	0,09	0,06	0,21	0,36
202255_s_at	SIPA1L1	14	q24.2	71065794	71275871		a	4,4E-03	0,33	FE0ADELA7ZH04	71015652	71147599	IN.e	0,06	0,23	0,14	0,07
239582_at	**PML**	15	q24.1	72126749	72127215		a	3,8E-04	0,32	FE0ADELA10ZB10	71930953	72092985	OUT.s	0,03	0,09	0,21	0,14
219229_at	SLCO3A1	15	q26.1	90197949	90507783		a	3,3E-04	0,68	FE0DBACA2ZD03	89800041	89955265	OUT.s	0,13	0,09	0,29	0,14
227048_at	LAMA1	18	p11.31	6931743	6940970	Cell Communication // ECM-receptor interaction // Focal adhesion // Prion disease // Small cell lung cancer	a	3,1E-05	0,69	FE0DBACA2ZE12	6626632	6760758	OUT.s	0,09	0,16	0,14	0,71
225016_at	APCDD1	18	p11.22	10444647	10478698		a	8,9E-04	1,06	FE0DBACA13ZC05	9999836	10178681	OUT.s	0,09	0,16	0,21	0,36
222869_s_at	ELAC1	18	q21.2	46748383	46768487		a	2,9E-04	0,55	FE0ADELA8ZC04	46732724	46887984	IN.se	0,03	0,29	0,07	0,50
203240_at	FCGBP	19	q13.2	45045810	45132373		a	1,9E-05	1,15	FE0DBACA7ZC05	43987923	44207444	OUT.s	0,03	0,00	0,29	0,29
209726_at	CA11	19	q13.33	53833083	53841069		a	8,6E-04	0,87	FE0DBACA1ZF02	52549610	52717102	OUT.s	0,09	0,03	0,36	0,00
219011_at	PLEKHA4	19	q13.33	54032166	54063670		a	2,5E-03	0,47	FE0DBACA1ZF02	52549610	52717102	OUT.s	0,09	0,03	0,36	0,00
235182_at	C20orf82	20	p12.1	13228345	13229298		a	6,8E-04	1,12	FE0BPADA6ZC01	12584077	12761267	OUT.s	0,31	0,03	0,71	0,07
1568742_at	NA	20	q13.33	58333293	58334599	NA	a	2,2E-05	0,32	FE0DBACA7ZA04	57746350	57934405	OUT.s	0,31	0,00	0,50	0,21
54051_at	PKNOX1	21	q22.3	43325413	43327023		a	1,5E-03	0,15	FE0BPADA6ZC08	43237444	43423508	IN.se	0,09	0,03	0,07	0,21
227051_at	NA	21	q22.3	41572895	41576325	NA	a	5,5E-04	1,23	FE0DBACA16ZG10	41111032	41272665	OUT.s	0,06	0,13	0,07	0,21
222062_at	IL27RA	NA	NA	NA	NA		a	2,1E-03	1,00	NA	NA	NA	NA	NA	NA	NA	NA
204061_at	PRKX	X	p22.33	3532412	3641649	Calcium signaling pathway // Gap junction // GnRH signaling pathway // Hedgehog signaling pathway // Insulin signaling pathway // Long-term potentiation // MAPK signaling pathway // Melanogenesis // Olfactory transduction // Taste transduction // Wnt signaling pathway	a	3,9E-03	0,87	FE0ADELA8ZD06	3114773	3306982	OUT.s	0,31	0,03	0,29	0,43
218332_at	BEX1	X	q22.1	102204236	102205737		a	1,6E-04	1,95	FE0DBACA22ZC10	101868569	102092519	OUT.s	0,09	0,09	0,07	0,29
228742_at	NA	Y	p11.31	2494069	2494660	NA	a	3,8E-03	0,47	NA	NA	NA	NA	NA	NA	NA	NA
1566965_at	**DPYD**	1	p21.3	97367213	97367926	Pantothenate and CoA biosynthesis // Pyrimidine metabolism // beta-Alanine metabolism	b	3,0E-03	-0,14	FE0DBACA14ZF01	96524039	96670384	OUT.s	0,06	0,22	0,14	0,57
1561892_at	ZMYM6	1	p34.3	35244219	35245780		b	5,4E-04	-0,18	FE0ADELA1ZD03	34840968	34967050	OUT.s	0,00	0,16	0,14	0,36
202017_at	**EPHX1**	1	q42.12	224079598	224099884	Metabolism of xenobiotics by cytochrome P450	b	3,4E-04	-0,95	FE0DBACA1ZD11	223414280	223593296	OUT.s	0,47	0,00	0,71	0,00
1561411_at	NA	1	q31.3	197241793	197256789	NA	b	1,0E-03	-0,20	FE0DBACA15ZH05	196801681	196953076	OUT.s	0,47	0,00	0,64	0,00
215328_at	EFR3B	2	p23.3	25118502	25224261		b	4,7E-03	-0,16	FE0DBACA17ZD11	24912656	25171590	IN.e	0,06	0,09	0,07	0,07
1553188_s_at	PARD3B	2	q33.2	205118760	206188782		b	2,6E-03	-0,19	FE0DBACA9ZA06	206169966	206382976	IN.s	0,03	0,09	0,00	0,29
225709_at	ARL6IP6	2	q23.3	153282673	153326013		b	4,9E-03	-0,21	FE0DBACA8ZC12	151982665	152174394	OUT.s	0,19	0,09	0,07	0,36
239192_at	PARD3B	2	q33.2	205118760	205538190		b	3,0E-03	-0,12	FE0DBACA18ZE04	204940027	204966390	OUT.s	0,03	0,09	0,00	0,29
207398_at	HOXD13	2	q31.1	176665777	176667732		b	7,1E-04	-0,25	FE0BPADA6ZE12	176341416	176532053	OUT.s	0,09	0,09	0,00	0,21
240483_at	ZNF717	3	p12.3	75872973	75873578		b	5,3E-04	-0,23	FE0ADELA4ZF07	74333644	74492463	OUT.s	0,04	0,29	0,10	0,40
221133_s_at	CLDN18	3	q22.3	139211748	139232694	Cell adhesion molecules (CAMs) // Leukocyte transendothelial migration // Tight junction	b	2,4E-03	-0,15	FE0ADELA4ZE08	138893610	139047468	OUT.s	0,00	0,00	0,00	0,00
207396_s_at	ALG3	3	q27.1	185442906	185449453	Glycan structures - biosynthesis 1 // N-Glycan biosynthesis	b	3,6E-03	-0,40	FE0DBACA17ZF04	184871891	185039510	OUT.s	0,22	0,00	0,29	0,07
232756_at	KALRN	3	q21.2	125642981	125646172		b	4,1E-03	-0,16	FE0BPADA8ZD05	125400950	125564390	OUT.s	0,09	0,13	0,29	0,00
204743_at	TAGLN3	3	q13.2	113200912	113215420		b	1,3E-03	-0,26	FE0DBACA17ZB03	112433580	112599755	OUT.s	0,16	0,09	0,00	0,36
230725_at	NA	5	q15	94063384	94076381	NA	b	6,6E-04	-0,17	FE0DBACA19ZA05	93979406	94091427	IN.se	0,20	0,17	0,33	0,33
232035_at	HIST1H4H	6	p22.1	26389332	26393741		b	4,5E-03	-1,48	FE0DBACA28ZB05	26134514	26284914	OUT.s	0,00	0,00	0,00	0,00
214516_at	HIST1H4B	6	p22.1	26135012	26135484		b	9,2E-04	-0,19	FE0DBACA28ZA05	26033294	26183414	IN.se	0,13	0,03	0,29	0,07
208496_x_at	HIST1H3G	6	p22.1	26379180	26379591		b	3,9E-03	-0,46	FE0DBACA28ZB05	26134514	26284914	OUT.s	0,00	0,00	0,00	0,00
1563511_at	DKFZp451B082	6	q26	163679363	163688055		b	1,1E-03	-0,16	FE0APADA1ZC05	163146613	163343895	OUT.s	0,03	0,25	0,00	0,71
223998_at	TTLL2	6	q27	167658595	167675323		b	4,8E-04	-0,14	FE0DBACA19ZA09	167030525	167160672	OUT.s	0,00	0,00	0,00	0,00
203694_s_at	DHX16	6	NA	2069456	2089337		b	4,3E-03	-0,28	FE0BPADA5ZH02	1942880	2096921	IN.se	0,06	0,09	0,21	0,21
236510_at	NA	6	NA	1726080	1726738	NA	b	1,1E-03	-0,29	FE0BPADA8ZC08	1381097	1568749	OUT.s	0,06	0,09	0,21	0,29
236967_at	NA	7	q36.3	156496360	156501881	NA	b	4,4E-03	-0,84	FE0DBACA5ZC06	156032397	156182661	OUT.s	0,22	0,00	0,29	0,29
1569361_a_at	NBR2	8	q11.23	55540707	55542541		b	9,3E-04	-0,15	FE0ADELA15ZC04	54844920	55029042	OUT.s	0,38	0,09	0,57	0,07
227774_s_at	IMPAD1	8	q12.1	58041355	58068542		b	4,6E-03	-0,22	FE0BPADA11ZE05	57073697	57235953	OUT.s	0,38	0,06	0,57	0,07
1557885_at	C9orf41	9	q21.13	76757703	76802097		b	2,6E-04	-0,15	FE0DBACA12ZG03	76025411	76195645	OUT.s	0,03	0,10	0,00	0,36
220902_at	FLJ12616	10	q26.13	125752559	125754480		b	1,3E-03	-0,25	FE0DBACA20ZG12	125384674	125476488	OUT.s	0,00	0,16	0,21	0,29
224411_at	PLA2G12B	10	q22.1	74365345	74384486	Arachidonic acid metabolism // Ether lipid metabolism // Fc epsilon RI signaling pathway // Glycerophospholipid metabolism // GnRH signaling pathway // Linoleic acid metabolism // Long-term depression // MAPK signaling pathway // VEGF signaling pathway	b	4,0E-03	-0,13	FE0BPADA11ZG09	74242499	74396459	IN.se	0,03	0,09	0,14	0,29
1558262_at	SPTY2D1	11	p15.1	18581087	18582006		b	1,9E-03	-0,12	FE0ADELA7ZH12	18398009	18551519	OUT.s	0,00	0,00	0,00	0,00
220303_at	PDZD3	11	q23.3	118561393	118565863		b	1,3E-04	-0,25	FE0ADELA15ZC09	117764287	117952972	OUT.s	0,03	0,44	0,00	0,64
231625_at	SLC22A9	11	q12.3	62897784	62930674		b	3,0E-03	-0,19	FE0DBACA10ZD07	62459050	62622184	OUT.s	0,06	0,13	0,00	0,21
1552526_at	FAM71C	12	q23.1	98565661	98567955		b	1,7E-03	-0,22	FE0ADELA1ZH11	98274728	98406085	OUT.s	0,09	0,06	0,36	0,14

Supplementary table 5: list of probe sets defining the transcritomic signature of the G2I-3 tumours and mapping on array-CGH BAC clones

1560573_at	LOC387895	12	q24.32	125781199	125822908		b	4,2E-03	-0,16	FE0DBACA10ZD03	125229752	125398297	OUT.s	0,06	0,09	0,21	0,07
217603_at	ATP6V0A2	12	q24.31	122804389	122805284	Cholera - Infection // Epithelial cell signaling in Helicobacter pylori infection // Oxidative phosphorylation	b	3,4E-03	-0,20	FE0DBACA15ZG02	122467061	122617425	OUT.s	0,06	0,06	0,29	0,07
1562722_at	NBR2	13	q21.1	56639331	56642353		b	1,5E-03	-0,19	FE0DBACA21ZG08	56555820	56758789	IN.se	0,03	0,34	0,07	0,50
215041_s_at	DOCK9	13	q32.3	98338766	98536648		b	2,6E-03	-0,24	FE0BPADA5ZA11	97949385	98122616	OUT.s	0,06	0,25	0,21	0,36
208034_s_at	PROZ	13	q34	112860968	112874695		b	9,1E-04	-0,21	FE0DBACA10ZF04	112314989	112494932	OUT.s	0,03	0,34	0,14	0,29
1569699_at	AK7	14	q32.2	95928230	95935381	Purine metabolism	b	1,8E-03	-0,23	FE0DBACA25ZF01	95222988	95384707	OUT.s	0,00	0,00	0,00	0,00
1570156_s_at	FMN1	15	q13.3	30849385	30850684		b	4,5E-03	-0,33	FE0DBACA7ZD12	30654184	30830821	OUT.s	0,00	0,28	0,07	0,43
1555437_at	EARS2	16	p12.1	23443843	23476166	Aminoacyl-tRNA biosynthesis // Glutamate metabolism // Porphyrin and chlorophyll metabolism	b	1,5E-03	-0,17	FE0BPADA8ZF04	22201758	22344702	OUT.s	0,34	0,03	0,71	0,00
1561092_at	KLHDC4	16	q24.2	85959936	86317302		b	7,4E-04	-0,17	FE0DBACA22ZB02	85825997	85913555	OUT.s	0,06	0,50	0,14	0,50
229056_at	**TP53I13**	17	q11.2	24918604	24922833		b	1,6E-03	-0,22	FE0DBACA24ZF11	23991558	24125208	OUT.s	0,09	0,16	0,29	0,00
232987_at	NBR2	17	q21.32	41958894	41961406		b	1,9E-04	-0,23	FE0ADELA8ZE08	41844639	42228602	IN.se	0,19	0,34	0,36	0,21
1560078_at	**LAMA3**	18	q11.2	19523744	19695766	Cell Communication // ECM-receptor interaction // Focal adhesion // Small cell lung cancer	b	4,1E-03	-0,34	FE0DBACA23ZF09	19307985	19450405	OUT.s	0,09	0,16	0,36	0,21
211362_s_at	SERPINB13	18	q21.33	59406862	59415650		b	2,2E-03	-0,19	FE0ADELA9ZE04	59104900	59292908	OUT.s	0,09	0,25	0,14	0,43
227881_s_at	C18orf17	18	q11.2	19848853	19854250		b	3,6E-05	-0,28	FE0DBACA23ZF09	19307985	19450405	OUT.s	0,09	0,16	0,36	0,21
217316_at	OR7A10	19	p13.12	14812759	14813689		b	3,6E-03	-0,21	FE0BPADA4ZH07	14304753	14499555	OUT.s	0,06	0,03	0,36	0,14
224011_at	SLC1A5	19	q13.32	51982911	51984136		b	5,7E-05	-0,24	FE0DBACA7ZA02	51898533	52062055	IN.se	0,03	0,03	0,31	0,15
1554952_s_at	NLRP12	19	q13.41	58988808	59019460		b	3,0E-03	-0,20	FE0DBACA2ZH02	57749715	57900027	OUT.s	0,09	0,00	0,36	0,00
209097_s_at	**JAG1**	20	p12.2	10566333	10602755	Notch signaling pathway	b	1,8E-03	-0,27	FE0DBACA28ZE02	10477896	10618405	IN.se	0,22	0,06	0,71	0,07
219570_at	KIF16B	20	p12.1	16200749	16302999		b	1,6E-03	-0,82	FE0DBACA1ZA07	15271792	15416382	OUT.s	0,28	0,09	0,71	0,07
214157_at	**GNAS**	20	q13.32	56850151	56909306	Calcium signaling pathway // Cholera - Infection // Gap junction // GnRH signaling pathway // Long-term depression // Melanogenesis // Taste transduction	b	6,2E-04	-1,33	FE0DBACA3ZH10	55772261	55922598	OUT.s	0,39	0,00	0,57	0,14
217917_s_at	**DYNLRB1**	20	q11.22	32567878	32592421		b	4,0E-03	-0,30	FE0ADELA11ZE12	31992437	32133000	OUT.s	0,25	0,03	0,64	0,14
213491_x_at	RPN2	20	q11.23	35285209	35303436	Glycan structures - biosynthesis 1 // N-Glycan biosynthesis	b	2,8E-03	-0,42	FE0ADELA9ZA06	35073715	35239014	OUT.s	0,25	0,06	0,71	0,00
1554857_at	ELMO2	20	q13.12	44434881	44451176		b	4,4E-04	-0,22	FE0DBACA25ZG07	43536618	43662990	OUT.s	0,32	0,00	0,71	0,00
208689_s_at	RPN2	20	q11.23	35241136	35303233	Glycan structures - biosynthesis 1 // N-Glycan biosynthesis	b	1,4E-03	-0,43	FE0ADELA9ZA06	35073715	35239014	OUT.s	0,25	0,06	0,71	0,00
241362_at	C20orf117	20	q11.23	34845355	34846091		b	1,7E-03	-0,20	FE0BPADA11ZE06	34169000	34346591	OUT.s	0,25	0,00	0,71	0,00
213399_x_at	RPN2	20	q11.23	35294171	35303436	Glycan structures - biosynthesis 1 // N-Glycan biosynthesis	b	1,6E-03	-0,41	FE0ADELA9ZA06	35073715	35239014	OUT.s	0,25	0,06	0,71	0,00
1552652_at	HPS4	22	q12.1	25177445	25209820		b	1,9E-03	-0,19	FE0DBACA6ZG04	24369614	24533971	OUT.s	0,06	0,25	0,07	0,43
223039_at	C22orf13	22	q11.23	23266409	23267514		b	7,9E-04	-0,49	FE0DBACA23ZC03	22867215	23061304	OUT.s	0,09	0,25	0,07	0,36
226133_s_at	TBC1D10A	22	q12.2	29017979	29052914		b	1,6E-03	-0,26	FE0ADELA13ZF05	28374240	28545060	OUT.s	0,13	0,19	0,14	0,36
204646_at	**DPYD**	1	p21.3	97315889	98159167	Pantothenate and CoA biosynthesis // Pyrimidine metabolism // beta-Alanine metabolism	a	1,4E-03	0,75	FE0BPADA5ZA10	97942019	98136193	IN.se	0,06	0,22	0,14	0,57
204363_at	**F3**	1	p21.3	94767460	94779903	Complement and coagulation cascades	a	2,3E-03	0,89	FE0ADELA7ZC03	94503701	94707287	OUT.s	0,09	0,19	0,07	0,57
221898_at	**PDPN**	1	p36.21	13815522	13817039		a	1,2E-03	0,69	FE0DBACA14ZD02	13714504	13848606	IN.se	0,00	0,34	0,14	0,43
202236_s_at	**SLC16A1**	1	p13.2	113256972	113273465		a	1,6E-03	0,99	FE0DBACA1ZG07	112869167	113015766	OUT.s	0,03	0,16	0,14	0,43
202234_s_at	**SLC16A1**	1	p13.2	113255991	113300317		a	1,7E-03	0,80	FE0BPADA6ZD05	113282311	113471064	IN.s	0,06	0,13	0,14	0,43
209900_s_at	**SLC16A1**	1	p13.2	113256375	113300328		a	2,7E-03	0,84	FE0BPADA6ZD05	113282311	113471064	IN.s	0,06	0,13	0,14	0,43
209413_at	B4GALT2	1	p34.1	44217693	44229426	Galactose metabolism // Glycan structures - biosynthesis 1 // Glycan structures - biosynthesis 2 // Glycosphingolipid biosynthesis - neo-lactoseries // Keratan sulfate biosynthesis // N-Glycan biosynthesis	a	2,2E-03	0,32	FE0ADELA1ZG08	43832683	43969267	OUT.s	0,09	0,06	0,14	0,43
220769_s_at	WDR78	1	p31.3	67052243	67113149		a	3,9E-04	0,54	FE0BPADA4ZA03	66606377	66787022	OUT.s	0,06	0,09	0,21	0,29
228297_at	CNN3	1	p21.3	95135100	95135515		a	1,2E-03	0,68	FE0ADELA7ZC03	94503701	94707287	OUT.s	0,09	0,19	0,07	0,57
202049_s_at	ZMYM4	1	p34.3	35507154	35660127		a	1,9E-03	0,44	FE0ADELA1ZA10	35476386	35592820	IN.e	0,00	0,16	0,14	0,36
219905_at	ERMAP	1	p34.2	43081930	43083242		a	2,4E-03	0,44	FE0ADELA7ZF05	42569840	42722857	OUT.s	0,16	0,06	0,14	0,36
235122_at	NA	1	p34.2	41744623	41746967	NA	a	6,7E-04	0,72	FE0ADELA1ZF08	41165048	41301394	OUT.s	0,09	0,13	0,14	0,36
202377_at	NA	1	p31.3	65658925	65672077	NA	a	2,5E-03	0,43	FE0DBACA15ZF05	65395414	65593152	OUT.s	0,03	0,10	0,21	0,29
204268_at	**S100A2**	1	q21.3	151800444	151804632		a	1,3E-03	1,43	FE0ADELA10ZB11	151108642	151284417	OUT.s	0,41	0,03	0,64	0,00
212609_s_at	**AKT3**	1	q44	241729648	241733354	Adipocytokine signaling pathway // Apoptosis // B cell receptor signaling pathway // Chronic myeloid leukemia // Colorectal cancer // Endometrial cancer // ErbB signaling pathway // Fc epsilon RI signaling pathway // Focal adhesion // Glioma // Insulin signaling pathway // Jak-STAT signaling pathway // MAPK signaling pathway // Melanoma // Non-small cell lung cancer // Pancreatic cancer // Prostate cancer // Renal cell carcinoma // Small cell lung cancer // T cell receptor signaling pathway // Tight junction // Toll-like receptor signaling pathway // VEGF signaling pathway // mTOR signaling pathway	a	2,1E-03	0,58	FE0BPADA10ZB04	241049232	241213107	OUT.s	0,47	0,00	0,64	0,00
222880_at	**AKT3**	1	q44	241733820	242073095	Adipocytokine signaling pathway // Apoptosis // B cell receptor signaling pathway // Chronic myeloid leukemia // Colorectal cancer // Endometrial cancer // ErbB signaling pathway // Fc epsilon RI signaling pathway // Focal adhesion // Glioma // Insulin signaling pathway // Jak-STAT signaling pathway // MAPK signaling pathway // Melanoma // Non-small cell lung cancer // Pancreatic cancer // Prostate cancer // Renal cell carcinoma // Small cell lung cancer // T cell receptor signaling pathway // Tight junction // Toll-like receptor signaling pathway // VEGF signaling pathway // mTOR signaling pathway	a	2,5E-03	0,57	FE0BPADA10ZB04	241049232	241213107	OUT.s	0,47	0,00	0,64	0,00
209270_at	LAMB3	1	q32.2	207854944	207892297	Cell Communication // ECM-receptor interaction // Focal adhesion // Small cell lung cancer	a	2,2E-03	0,81	FE0DBACA14ZF05	207724559	207869795	IN.e	0,50	0,00	0,57	0,00
208274_at	OCLM	1	q31.1	184636326	184637210		b	1,2E-03	-0,16	FE0ADELA1ZB11	183590388	183773372	OUT.s	0,69	0,00	0,57	0,07
237098_at	C1orf21	1	q25.3	182852858	182853574		b	2,9E-03	-0,25	FE0DBACA13ZG12	182448584	182622218	OUT.s	0,47	0,00	0,64	0,00
226755_at	NA	1	q32.2	207670653	207672517	NA	a	1,1E-03	0,91	FE0ADELA1ZC11	206464881	206594557	OUT.s	0,50	0,00	0,57	0,00
1555942_a_at	NA	1	q32.2	207668786	207672516	NA	a	1,6E-03	0,43	FE0ADELA1ZC11	206464881	206594557	OUT.s	0,50	0,00	0,57	0,00

Supplementary table 5: list of probe sets defining the transcritomic signature of the G2I-3 tumours and mapping on array-CGH BAC clones

244428_at	**DNMT3A**	2	p23.3	25357825	25358721	Methionine metabolism	a	2,2E-04	0,53	FE0ADELA4ZG03	25347585	25544449	IN.se	0,06	0,09	0,07	0,07
240130_at	NA	2	p16.3	48640685	48641111	NA	b	2,4E-03	-0,13	FE0ADELA9ZF04	47865648	48007408	OUT.s	0,09	0,06	0,00	0,07
227449_at	EPHA4	2	q36.1	221990998	221991867	Axon guidance	a	1,5E-03	0,66	FE0BPADA13ZA01	221296967	221446303	OUT.s	0,09	0,13	0,00	0,21
242828_at	FIGN	2	q24.3	164170631	164171446		a	1,2E-03	0,50	FE0DBACA18ZF02	163626982	163795524	OUT.s	0,13	0,09	0,00	0,29
221935_s_at	C3orf64	3	p14.1	69107052	69109490		a	1,5E-03	0,91	FE0BPADA7ZB11	68279314	68457599	OUT.s	0,03	0,34	0,14	0,29
229831_at	CNTN3	3	p12.3	74394411	74618331		a	1,9E-03	1,17	FE0DBACA5ZA03	74333643	74492281	IN.e	0,06	0,34	0,07	0,43
209863_s_at	**TP63**	3	q28	190990159	191097749		a	2,7E-03	1,25	FE0APADA2ZB11	190906402	191068102	IN.e	0,22	0,03	0,14	0,29
211194_s_at	**TP63**	3	q28	190990225	191095984		a	2,8E-03	0,71	FE0APADA2ZB11	190906402	191068102	IN.e	0,22	0,03	0,14	0,29
205227_at	IL1RAP	3	q28	191714584	191851995	Apoptosis // Cytokine-cytokine receptor interaction	a	6,0E-04	0,33	FE0ADELA4ZC09	191651376	191835476	IN.e	0,00	0,00	0,50	0,50
218717_s_at	LEPREL1	3	q28	191157315	191320803		a	6,3E-04	0,79	FE0APADA2ZB11	190906402	191068102	OUT.s	0,22	0,03	0,14	0,29
202555_s_at	MYLK	3	q21.1	124815581	124903051	Calcium signaling pathway // Focal adhesion // Regulation of actin cytoskeleton	a	8,8E-04	0,85	FE0DBACA1ZG10	123351131	123501875	OUT.s	0,09	0,09	0,21	0,07
210079_x_at	KCNAB1	3	q25.31	157343594	157737497		b	8,9E-04	-0,23	FE0BPADA11ZA10	156874008	157024204	OUT.s	0,00	0,00	0,50	0,00
224823_at	MYLK	3	q21.1	124813773	124815680	Calcium signaling pathway // Focal adhesion // Regulation of actin cytoskeleton	a	2,9E-03	0,82	FE0DBACA1ZG10	123351131	123501875	OUT.s	0,09	0,09	0,21	0,07
220733_at	SLC26A1	4	p16.3	971772	977183		b	1,8E-03	-0,24	FE0ADELA13ZG08	55703	207193	OUT.s	0,00	0,09	0,07	0,36
204924_at	**TLR2**	4	q31.3	154843487	154845980	Toll-like receptor signaling pathway	a	2,4E-03	0,58	FE0BPADA7ZA08	154717641	154885253	IN.se	0,13	0,03	0,14	0,36
221942_s_at	GUCY1A3	4	q32.1	156857818	156872180	Gap junction // Long-term depression // Purine metabolism	a	9,3E-04	0,97	FE0BPADA12ZC01	156675950	156846238	OUT.s	0,13	0,06	0,14	0,43
227088_at	PDE5A	4	q27	120635001	120636706	Purine metabolism	a	2,1E-03	1,05	FE0APADA2ZH05	120171769	120330659	OUT.s	0,13	0,03	0,14	0,29
1556698_a_at	GPRIN3	4	q22.1	90384448	90391368		a	2,2E-03	0,31	FE0DBACA23ZF12	89342577	89493214	OUT.s	0,09	0,03	0,21	0,36
229530_at	NA	4	q32.1	156873364	156874431	NA	a	3,0E-04	0,98	FE0BPADA12ZC01	156675950	156846238	OUT.s	0,13	0,06	0,14	0,43
205405_at	SEMA5A	5	p15.2	9091858	9599158	Axon guidance	a	1,6E-04	0,72	FE0DBACA11ZG07	9480400	9682601	IN.s	0,16	0,00	0,14	0,21
220076_at	ANKH	5	p15.2	14757909	14924616		a	4,0E-04	0,63	FE0BPADA7ZH03	13126726	13314258	OUT.s	0,19	0,03	0,14	0,29
244650_at	FAM105A	5	p15.2	14664467	14665027		a	2,6E-03	0,64	FE0BPADA7ZH03	13126726	13314258	OUT.s	0,19	0,03	0,14	0,29
205117_at	**FGF1**	5	q31.3	141951926	142045820	MAPK signaling pathway // Melanoma // Regulation of actin cytoskeleton	a	2,9E-03	0,72	FE0ADELA15ZF01	141852242	141998311	IN.e	0,19	0,13	0,21	0,07
219932_at	SLC27A6	5	q23.3	128329576	128396887	PPAR signaling pathway	a	4,5E-04	0,77	FE0DBACA19ZA03	128378376	128611237	IN.s	0,19	0,13	0,14	0,14
226576_at	ARHGAP26	5	q31.3	142587059	142588763		a	6,2E-04	0,89	FE0DBACA20ZF03	142528844	142679287	IN.se	0,22	0,16	0,21	0,29
244548_at	ARHGAP26	5	q31.3	142306312	142306869		a	6,8E-04	0,30	FE0DBACA19ZF02	141941600	142028404	OUT.s	0,22	0,13	0,21	0,07
205068_s_at	ARHGAP26	5	q31.3	142130475	142586243		a	1,7E-03	0,67	FE0DBACA20ZF03	142528844	142679287	IN.s	0,22	0,16	0,21	0,29
227056_at	KIAA0141	5	q31.3	141293045	141295232		a	2,9E-03	0,40	FE0DBACA4ZG04	141031072	141196441	OUT.s	0,19	0,16	0,29	0,07
220375_s_at	NA	5	q31.1	134396868	134708269	NA	b	2,3E-03	-0,24	FE0DBACA16ZG06	134419761	134591443	IN.se	0,19	0,19	0,29	0,07
208569_at	HIST1H2AB	6	p22.1	26141382	26141775		b	6,0E-04	-0,20	FE0DBACA28ZA05	26033294	26183414	IN.se	0,13	0,03	0,29	0,07
234682_at	BTBD9	6	p21.2	38668240	38673848		b	6,0E-04	-0,17	FE0BPADA6ZB07	38488141	38660731	OUT.s	0,16	0,00	0,29	0,14
209911_x_at	HIST1H2BD	6	p22.1	26266351	26279553		b	2,5E-03	-0,99	FE0DBACA28ZB05	26134514	26284914	IN.se	0,00	0,00	0,00	0,00
203836_s_at	MAP3K5	6	q23.3	136920458	137155257	MAPK signaling pathway	a	2,1E-03	0,80	FE0ADELA13ZG07	136892735	137046976	IN.e	0,16	0,25	0,14	0,57
237347_at	KHDC1	6	q13	74031348	74031979		a	1,2E-03	0,44	FE0BPADA4ZF07	73016383	73175246	OUT.s	0,06	0,16	0,07	0,50
1556211_a_at	AIG1	6	q24.2	143699221	143701999		a	1,5E-03	0,54	FE0DBACA3ZE11	143362387	143512585	OUT.s	0,16	0,25	0,07	0,64
229317_at	KPNA5	6	q22.2	117162729	117163496		a	2,0E-03	0,68	FE0BPADA7ZF09	115807782	116004630	OUT.s	0,09	0,28	0,00	0,71
223886_s_at	RNF146	6	q22.33	127629712	127651200		a	2,2E-03	0,36	FE0BPADA12ZE08	127510880	127664223	IN.se	0,13	0,28	0,00	0,71
204165_at	WASF1	6	q21	110527714	110606609	Adherens junction // Regulation of actin cytoskeleton	a	2,5E-03	0,81	FE0DBACA5ZE08	109464083	109649020	OUT.s	0,09	0,28	0,07	0,57
202318_s_at	SENP6	6	q14.1	76368945	76482326		a	2,8E-03	0,43	FE0ADELA7ZF01	76145606	76312627	OUT.s	0,03	0,22	0,00	0,69
243904_at	NA	6	q24.3	147751269	147752298	NA	a	5,1E-04	0,38	FE0BPADA6ZH03	147132229	147329173	OUT.s	0,16	0,25	0,07	0,64
243719_at	STK19	6	NA	3393557	3394054		b	2,1E-03	-0,20	FE0DBACA5ZC08	2641344	2815440	OUT.s	0,06	0,09	0,21	0,21
235229_at	NA	6	NA	777607	779212	NA	a	1,3E-03	1,15	FE0DBACA24ZC08	230896	357473	OUT.s	0,09	0,13	0,21	0,29
213943_at	**TWIST1**	7	p21.1	19121615	19123809		a	1,1E-03	0,86	FE0DBACA19ZC09	18859764	19033257	OUT.s	0,28	0,03	0,29	0,14
229228_at	CREB5	7	p15.1	28831565	28832033	Prostate cancer	a	1,6E-03	0,41	FE0DBACA2ZE04	28560076	28724097	OUT.s	0,22	0,06	0,29	0,00
239218_at	NA	7	p15.1	31789663	31790010	NA	a	1,5E-03	0,32	FE0DBACA19ZD10	30721972	30874417	OUT.s	0,22	0,00	0,29	0,00
209993_at	**ABCB1**	7	q21.12	86971261	87068119	ABC transporters - General	a	3,8E-04	0,49	FE0ADELA15ZF03	86539096	86700050	OUT.s	0,25	0,00	0,29	0,43
209465_x_at	**PTN**	7	q33	136562627	136679118		a	1,3E-03	1,16	FE0ADELA9ZA05	136491374	136652222	IN.e	0,22	0,03	0,29	0,29
228110_x_at	NA	7	q11.21	65842259	65843112	NA	b	2,3E-03	-0,50	FE0BPADA11ZG05	65773246	65925888	IN.se	0,16	0,00	0,36	0,07
200762_at	DPYSL2	8	p21.2	26491372	26571607	Axon guidance	a	2,3E-03	0,78	FE0DBACA4ZD02	26308669	26476364	OUT.s	0,00	0,50	0,00	1,00
210397_at	DEFB1	8	p23.1	6715568	6722860		a	2,3E-03	0,97	FE0BPADA5ZF03	6182469	6364998	OUT.s	0,09	0,47	0,07	0,64
238458_at	EFHA2	8	p22	17023322	17024516		a	4,8E-04	0,39	FE0DBACA12ZC02	15547187	15733616	OUT.s	0,09	0,47	0,07	0,64
222731_at	ZDHHC2	8	p22	17058206	17124612		a	5,1E-04	1,02	FE0DBACA12ZC02	15547187	15733616	OUT.s	0,09	0,47	0,07	0,64
232302_at	PDLIM2	8	p21.3	22493988	22511483		b	1,7E-03	-0,13	FE0APADA1ZA02	22048391	22230663	OUT.s	0,09	0,47	0,07	0,64
222730_s_at	ZDHHC2	8	p22	17058206	17124612		a	2,7E-03	0,69	FE0DBACA12ZC02	15547187	15733616	OUT.s	0,09	0,47	0,07	0,64
204076_at	ENTPD4	8	p21.3	23342619	23371106	Purine metabolism // Pyrimidine metabolism	a	2,9E-03	0,41	FE0DBACA12ZD02	23345195	23521961	IN.s	0,09	0,50	0,07	0,64
229568_at	MOBKL2B	9	p21.2	27315207	27315813		a	6,7E-05	0,78	FE0DBACA27ZD11	27163232	27323328	IN.se	0,03	0,31	0,07	0,64
236100_at	FBXO10	9	p13.2	37531238	37566250		b	7,8E-04	-0,18	FE0ADELA10ZA10	36895443	37054507	OUT.s	0,13	0,22	0,07	0,36
226221_at	KIAA1432	9	p24.1	5743207	5767038		a	1,2E-03	0,33	FE0BPADA4ZG06	5009594	5181448	OUT.s	0,16	0,16	0,07	0,64
237169_at	**TNC**	9	q33.1	116821677	116822177	Cell Communication // ECM-receptor interaction // Focal adhesion	a	2,2E-03	0,63	FE0ADELA15ZA06	116408174	116563549	OUT.s	0,06	0,13	0,07	0,21
201645_at	**TNC**	9	q33.1	116822633	116920260	Cell Communication // ECM-receptor interaction // Focal adhesion	a	2,4E-03	1,44	FE0ADELA15ZA06	116408174	116563549	OUT.s	0,06	0,13	0,07	0,21
224941_at	PAPPA	9	q33.1	118200432	118203974		a	8,6E-04	0,78	FE0DBACA25ZF04	117345113	117478548	OUT.s	0,06	0,13	0,07	0,21
224942_at	PAPPA	9	q33.1	118200432	118203974		a	1,8E-03	0,51	FE0DBACA25ZF04	117345113	117478548	OUT.s	0,06	0,13	0,07	0,21
236233_at	TRIM32	9	q33.1	118502816	118503399		a	1,2E-03	0,39	FE0DBACA25ZF04	117345113	117478548	OUT.s	0,06	0,13	0,07	0,21
221840_at	PTPRE	10	q26.2	129729127	129774163		a	8,1E-04	0,82	FE0DBACA1ZA03	129558368	129708510	OUT.s	0,00	0,16	0,14	0,14
236144_at	CPXM2	10	q26.13	125496001	125506793		a	1,5E-03	0,57	FE0DBACA20ZG12	125384674	125476488	OUT.s	0,00	0,16	0,21	0,29
227614_at	HKDC1	10	q21.3	70688608	70697314		b	2,1E-03	-0,16	FE0DBACA11ZD02	70694754	70862995	IN.s	0,03	0,13	0,14	0,29
209835_x_at	**CD44**	11	p13	35117304	35207591	ECM-receptor interaction // Hematopoietic cell lineage	a	2,2E-03	0,94	FE0DBACA10ZD02	33914327	34095862	OUT.s	0,16	0,06	0,14	0,07
204489_s_at	**CD44**	11	p13	35117398	35208149	ECM-receptor interaction // Hematopoietic cell lineage	a	2,4E-03	0,91	FE0DBACA10ZD02	33914327	34095862	OUT.s	0,16	0,06	0,14	0,07
223708_at	**C1QTNF4**	11	p11.2	47567791	47572479		a	2,0E-03	0,25	FE0ADELA8ZF12	47194435	47352746	OUT.s	0,13	0,00	0,14	0,00
203278_s_at	PHF21A	11	p11.2	45910690	45943585		a	1,2E-03	0,29	FE0DBACA9ZF07	44720149	44923425	OUT.s	0,13	0,00	0,15	0,00
1554153_a_at	PHF21A	11	p11.2	45911609	46099252		a	2,0E-03	0,38	FE0BPADA11ZC04	46038629	46204140	IN.s	0,16	0,00	0,14	0,07
234958_at	PPFIBP2	11	p15.4	7492584	7494800		b	2,1E-03	-0,21	FE0DBACA9ZD07	6543782	6698976	OUT.s	0,09	0,13	0,07	0,14
205680_at	**MMP10**	11	q22.2	102146443	102156554		a	2,2E-03	1,62	FE0ADELA7ZA09	101950797	102132690	OUT.s	0,03	0,31	0,00	0,71
227727_at	MRGPRF	11	q13.2	68528433	68529578		a	1,9E-03	0,59	FE0DBACA1ZE01	68501091	68644746	IN.se	0,28	0,03	0,21	0,00
211904_x_at	**RAD52**	12	p13.33	904889	912485		b	2,3E-03	-0,22	FE0DBACA26ZG03	767281	912613	IN.se	0,13	0,06	0,36	0,07

Supplementary table 5: list of probe sets defining the transcritomic signature of the G2I-3 tumours and mapping on array-CGH BAC clones

206785_s_at	NA	12	p13.2	10474474	10479897	NA	a	2,3E-03	0,46	FE0DBACA9ZF01	9623735	9799377	OUT.s	0,09	0,06	0,36	0,29
213711_at	KRT81	12	q13.13	50965963	50971516	Cell Communication	a	2,6E-03	0,71	FE0DBACA14ZC10	50551168	50763736	OUT.s	0,06	0,03	0,43	0,00
232505_at	DCTN2	12	q13.3	56217965	56220099		b	2,7E-03	-0,18	FE0ADELA15ZE10	56048376	56258422	IN.se	0,09	0,00	0,50	0,07
213680_at	KRT6B	12	q13.13	51126701	51127546	Cell Communication	a	1,9E-03	1,85	FE0DBACA14ZC10	50551168	50763736	OUT.s	0,06	0,03	0,43	0,00
243891_at	P76	12	q24.13	112294001	112294528		b	2,3E-03	-0,32	FE0DBACA21ZH06	111640268	111790683	OUT.s	0,06	0,06	0,21	0,14
203413_at	NELL2	12	q12	43188331	43556405		a	2,9E-03	1,60	FE0DBACA14ZD12	42529874	42691001	OUT.s	0,06	0,06	0,29	0,21
1559924_at	NA	12	q13.11	45051411	45053828	NA	b	1,0E-03	-0,15	FE0DBACA9ZA01	44779104	44962703	OUT.s	0,03	0,06	0,43	0,07
241470_x_at	NA	12	q21.31	84718611	84718906	NA	a	2,0E-03	0,34	FE0DBACA5ZF10	84479444	84673896	OUT.s	0,13	0,06	0,36	0,36
226136_at	NA	12	q21.2	74179310	74181975	NA	a	2,0E-03	0,57	FE0DBACA9ZB01	73729731	73899705	OUT.s	0,10	0,10	0,46	0,23
232054_at	PCDH20	13	q21.31	60881821	60887282		a	1,2E-03	0,60	FE0DBACA10ZE10	60770296	60969701	IN.se	0,03	0,34	0,08	0,54
216569_at	NA	13	q14.11	41841349	41841793	NA	b	2,8E-03	-0,21	FE0BPADA4ZE03	41403116	41593081	OUT.s	0,00	0,34	0,00	0,43
240841_at	INSM2	14	q13.2	35075545	35076011		b	2,0E-03	-0,22	FE0DBACA21ZD09	33496774	33569017	OUT.s	0,13	0,06	0,29	0,21
215971_at	NA	14	q23.1	59634394	59635415	NA	b	1,6E-03	-0,20	FE0BPADA11ZD07	58786413	58957923	OUT.s	0,13	0,19	0,29	0,14
227367_at	SLCO3A1	15	q26.1	90508725	90510137		a	8,5E-05	0,68	FE0DBACA2ZD03	89800041	89955265	OUT.s	0,13	0,09	0,29	0,14
238868_at	UACA	15	q23	68733948	68734526		a	2,8E-03	0,43	FE0BPADA10ZG04	67720612	67874479	OUT.s	0,03	0,06	0,15	0,15
203828_s_at	IL32	16	p13.3	3055665	3059552		a	2,0E-03	0,77	FE0DBACA13ZB03	2779832	2953509	OUT.s	0,29	0,00	0,79	0,00
227256_at	USP31	16	p12.1	22980235	22981746		a	1,4E-03	0,71	FE0BPADA8ZF04	22201758	22344702	OUT.s	0,34	0,03	0,71	0,00
228118_x_at	C16orf13	16	p13.3	624430	626315		b	1,7E-03	-0,15	FE0ADELA8ZH07	86672	202986	OUT.s	0,34	0,03	0,71	0,00
232051_at	CCDC102A	16	q13	56103714	56112559		a	1,3E-03	0,35	FE0DBACA13ZA04	56002623	56156729	IN.se	0,03	0,53	0,14	0,43
1566680_at	PDLIM2	16	q23.1	77794020	77796340		b	1,8E-03	-0,18	FE0DBACA5ZC10	77599670	77781420	OUT.s	0,09	0,56	0,14	0,50
205031_at	EFNB3	17	p13.1	7549428	7555370	Axon guidance	a	2,0E-03	0,75	FE0DBACA23ZA02	7541951	7542427	OUT.s	0,00	0,47	0,15	0,69
213093_at	**PRKCA**	17	q24.2	62235503	62237322	Calcium signaling pathway // Cholera - Infection // ErbB signaling pathway // Fc epsilon RI signaling pathway // Focal adhesion // Gap junction // Glioma // GnRH signaling pathway // Leukocyte transendothelial migration // Long-term depression // Long-term potentiation // MAPK signaling pathway // Melanogenesis // Natural killer cell mediated cytotoxicity // Non-small cell lung cancer // Pathogenic Escherichia coli infection - EHEC // Pathogenic Escherichia coli infection - EPEC // Phosphatidylinositol signaling system // Tight junction // VEGF signaling pathway // Wnt signaling pathway	a	1,6E-03	0,70	FE0ADELA8ZF03	62228259	62408926	IN.se	0,28	0,06	0,43	0,14
205157_s_at	**KRT17**	17	q21.2	37029220	37034335	Cell Communication	a	1,4E-03	1,87	FE0DBACA16ZF01	35737426	35916210	OUT.s	0,13	0,22	0,29	0,21
212236_x_at	**KRT17**	17	q21.2	37029219	37034332	Cell Communication	a	1,5E-03	1,72	FE0DBACA16ZF01	35737426	35916210	OUT.s	0,13	0,22	0,29	0,21
1560563_at	NA	17	q25.3	78265073	78267418	NA	b	2,3E-03	-0,16	FE0ADELA8ZH08	78134453	78311260	IN.se	0,19	0,13	0,21	0,21
213924_at	MPPE1	18	p11.21	11874195	11875819		a	1,8E-04	0,72	FE0ADELA8ZB04	11468522	11648086	OUT.s	0,09	0,13	0,36	0,14
206355_at	GNAL	18	p11.21	11742185	11872233	Calcium signaling pathway // Olfactory transduction	a	2,3E-04	0,54	FE0ADELA8ZB04	11468522	11648086	OUT.s	0,09	0,13	0,36	0,14
206490_at	DLGAP1	18	p11.31	3492143	3835296		a	1,4E-03	0,30	FE0DBACA13ZG06	3607103	3765893	IN.se	0,09	0,16	0,14	0,71
204855_at	**SERPINB5**	18	q21.33	59295198	59323297		a	1,6E-03	1,68	FE0ADELA9ZE04	59104900	59292908	OUT.s	0,09	0,25	0,14	0,43
235075_at	**DSG3**	18	q12.1	27310854	27312666	Cell Communication	a	1,2E-03	1,02	FE0DBACA7ZF05	27064581	27208580	OUT.s	0,09	0,16	0,07	0,64
205595_at	**DSG3**	18	q12.1	27281755	27310474	Cell Communication	a	1,2E-03	1,01	FE0DBACA7ZF05	27064581	27208580	OUT.s	0,09	0,16	0,07	0,64
204297_at	**PIK3C3**	18	q12.3	37789207	37915358	Inositol phosphate metabolism // Phosphatidylinositol signaling system // Regulation of autophagy	a	9,2E-04	0,37	FE0DBACA6ZA11	37831231	37996975	IN.s	0,06	0,22	0,07	0,64
228681_x_at	DAPK3	19	p13.3	3909975	3910467		b	7,0E-04	-0,19	FE0BPADA9ZA03	3747566	3897695	OUT.s	0,06	0,06	0,14	0,14
224111_x_at	KLF16	19	p13.3	1803398	1814496		b	2,7E-03	-0,18	FE0BPADA7ZB10	1756515	1947280	IN.se	0,03	0,06	0,14	0,14
221417_x_at	S1PR5	19	p13.2	10485490	10486687	Neuroactive ligand-receptor interaction	b	2,9E-03	-0,15	FE0ADELA8ZA05	9986246	10114243	OUT.s	0,13	0,06	0,29	0,21
222242_s_at	**KLK5**	19	q13.33	56138698	56147701		a	8,4E-04	1,35	FE0BPADA12ZA11	55987853	56202469	IN.se	0,09	0,03	0,43	0,00
232679_at	PAPPA	19	q13.11	39995071	39996323		a	1,8E-03	0,40	FE0DBACA6ZH12	38838477	39020980	OUT.s	0,03	0,09	0,14	0,29
223496_s_at	CCDC8	19	q13.32	51605930	51608681		a	2,1E-03	0,58	FE0DBACA26ZD08	50052290	50202708	OUT.s	0,06	0,03	0,29	0,21
229924_s_at	**JAG1**	20	p12.2	10587301	10602274	Notch signaling pathway	b	1,7E-03	-0,22	FE0DBACA28ZE02	10477896	10618405	IN.se	0,22	0,06	0,71	0,07
1569906_s_at	PHF20	20	q11.22	33823383	33983298		b	5,5E-04	-0,40	FE0BPADA5ZH06	33234286	33386029	OUT.s	0,25	0,00	0,64	0,14
229578_at	JPH2	20	q13.12	42019620	42174337		a	1,9E-03	0,55	FE0DBACA23ZC08	41725281	41887619	OUT.s	0,31	0,00	0,64	0,00
1561132_at	DHX35	20	q11.23	37085926	37086544		b	2,5E-03	-0,17	FE0DBACA6ZE12	36528191	36711090	OUT.s	0,25	0,00	0,71	0,00
241398_at	MPPED1	22	q13.2	42234490	42235052		b	2,9E-03	-0,22	FE0BPADA9ZF06	40929623	41097973	OUT.s	0,03	0,22	0,07	0,43
244758_at	ZNF452	NA	NA	NA	NA		a	2,6E-03	0,64	NA	NA	NA	NA	NA	NA	NA	NA
231147_at	NA	NA	NA	NA	NA	NA	b	7,4E-04	-0,18	NA	NA	NA	NA	NA	NA	NA	NA
1561910_at	NA	NA	NA	NA	NA	NA	b	7,5E-04	-0,22	NA	NA	NA	NA	NA	NA	NA	NA
230685_at	NA	NA	NA	NA	NA	NA	b	2,1E-03	-0,27	NA	NA	NA	NA	NA	NA	NA	NA
205541_s_at	GSPT2	X	p11.22	51503283	51505763		a	2,6E-04	1,42	FE0APADA1ZD04	51060132	51210370	OUT.s	0,03	0,13	0,14	0,36
219855_at	NUDT11	X	p11.22	51249603	51256100		a	3,3E-04	0,37	FE0APADA1ZD04	51060132	51210370	OUT.s	0,03	0,13	0,14	0,36
221933_at	NLGN4X	X	p22.32	5818068	6155888		a	1,2E-03	1,17	FE0APADA2ZA04	5831199	5996100	IN.se	0,06	0,13	0,21	0,50
236277_at	PAK3	X	q22.3	110258548	110259083	Axon guidance // ErbB signaling pathway // Focal adhesion // Regulation of actin cytoskeleton // Renal cell carcinoma // T cell receptor signaling pathway	a	1,1E-03	0,37	FE0BPADA5ZG02	108719433	108898995	OUT.s	0,06	0,09	0,14	0,21
227688_at	LRCH2	X	q23	114251434	114320321		a	1,0E-03	0,35	FE0DBACA15ZE10	114016334	114190846	OUT.s	0,06	0,09	0,14	0,36
203903_s_at	HEPH	X	q12	65300833	65403952		a	1,7E-03	0,75	FE0DBACA15ZG08	64678210	64856089	OUT.s	0,03	0,06	0,07	0,14
222235_s_at	CSGALNACT2	X	q22.3	105457417	105459476	Chondroitin sulfate biosynthesis // Glycan structures - biosynthesis 1	a	2,1E-03	0,36	FE0BPADA12ZB02	104914165	105076568	OUT.s	0,06	0,09	0,07	0,29

a) BAC clone status: OUT.s proximal to the 5'end of the probe set ; IN.s overlap of the 5'end of the probe set; IN.e overlap of the 3'end of the probe set; IN.se; spanning the probe set; NA not applicable

Supplementary table 6: gene ontology enrichment for the G2I-3 expression signature

name	***gene Symbol***	***gene Cluster***
Effects of calcineurin in Keratinocyte Differentiation	MARCKS;PPP3CC	a
ECM-receptor interaction	LAMA1;ITGB8;CD44	a
Natural killer cell mediated cytotoxicity	PPP3CC;KLRC3;PIK3CD	a
Hedgehog signaling pathway	PRKX;GAS1	a
Acute myeloid leukemia	PML;PIK3CD	a
B cell receptor signaling pathway	PIK3CD;PPP3CC	a
VEGF signaling pathway	PPP3CC;PIK3CD	a
Long-term potentiation	PPP3CC;PRKX	a
Focal adhesion	ITGB8;PIK3CD;LAMA1	a
Regulation of actin cytoskeleton	TIAM2;PIK3CD;ITGB8	a
Apoptosis	PPP3CC;PIK3CD	a
Small cell lung cancer	PIK3CD;LAMA1	a
T cell receptor signaling pathway	PIK3CD;PPP3CC	a
GnRH signaling pathway	MAP3K4;PRKX	a
MAPK signaling pathway	PRKX;MAP3K4;PPP3CC	a
N-Glycan biosynthesis	ALG3;RPN2	b
Cholera - Infection	GNAS;ATP6V0A2	b
Long-term depression	PLA2G12B;GNAS	b
GnRH signaling pathway	PLA2G12B;GNAS	b
Glycan structures - biosynthesis 1	RPN2;ALG3	b
