# Supplementary material for: An array CGH based genomic instability index (G2I) is predictive of clinical outcome in breast cancer and reveals a subset of tumors without lymph node involvement but with poor prognosis
Source: BMC Med Genomics. 2012 Nov 27;5:54. doi: 10.1186/1755-8794-5-54 (PMC3558323; doi:10.1186/1755-8794-5-54)
Supplement: Additional file 2 — Supplementary methods. File containing methodological details. [file 1755-8794-5-54-S2.doc]

**Supplementary methods**

**CGH array: hybridization and pre-treatment**

***1. DNA labeling and hybridization protocols:*** 0.6 µg of tumor DNA and 0.6 µg of a pool of 20 normal DNA were labeled with Cy3-dCTP and Cy5-dCTP (Amersham, GE Healthcare) respectively, using the BioPrime DNA Labeling System (Invitrogen, Cergy-Pontoise, France). Labeled products were purified on YM-30 Microcon centrifugal filters. Labeled tumor and reference DNAs were quantified on a NanoDrop 1000 spectrophotometer (Thermoscientific), ethanol-precipitated together with a 15-fold excess of human Cot-1 DNA (Roche) and 10µL of salmon sperm DNA, then resuspended in 63 µl of hybridization buffer (50% formamide, 10% dextran sulfate, 2X SSC). The probes were denatured at 100°C for 1 min 30 sec, and repetitive sequences were blocked by pre-annealing at 37°C for 30 min. Pre-annealed probes were loaded onto microarray slides that were enclosed in humidity chambers (Corning) and hybridized at 37°C for 17 h. Posthybridization washes were as follows: 1 wash at 54°C in SSC(2X) SDS(0.1%) for 5 min, followed by 2 washes at RT in SSC(1X), 1 wash at RT in SSC(0.2X), 1 wash at RT in water for 2 min, and 15 washes in isopropyl alcohol. Slides were dried by centrifugation 2 min at 400 rpm (Jouan, CR412). Slides were scanned with a Scanarray 4000 (Packard Instruments Inc…. USA) and analyzed with GenePix Pro 5.1 image analysis software, which defined the spots and determined the median intensities for the Cy3 and Cy5 signals of each BAC clone.

***2. Spot filtering and normalization:*** The following filtering parameters to discard spots were retained: signal to noise ratio for the reference channel less than 3, inter-replicate standard deviation larger than 0.1, number of residual replicates less than 2. All others parameters were default parameters. The mean percentage of remained clones by micro array was: 94.46 (SD: 1.69). The average variability among replicates was: 0.049 (range: 0.034 – 0.060). For clones in which at least two replicates remained after filtering, an average normalized log2-ratio value was calculated excluding clones with a median log ratio larger than 2 for the normalization process (and re-included afterwards).

***3. Smoothing***: the iterative, data-adaptive smoothing technique Adaptive Weights Smoothing (AWS, http://www.wias-berlin.de/project-areas/stat/publications/paper.html; Polzehl and Spokoiny) was then applied to the normalized log2-ratio values as adapted in the R GLAD package v1.8 (Ref 1) This yielded smoothed log2-ratios values in homogeneous segments along the chromosome.

***4. Determination of DNA copy number:*** for each sample, the level (L_N_) corresponding to a normal (i.e. diploid) copy number is determined as the first mode of the distribution of the smoothed log2-ratio values across all autosomes. The standard deviation (SD) of the difference between normalized and smoothed log2-ratio values is calculated. Then for all clones in a segment, the ‘GNL’ copy number status (G: gain - N: normal - L: loss) is determined as follows: based on the segment smoothed log-ratio value (X): if X > L_N_ + SD then status=gain (G), if X < L_N_ – SD then status=loss (L), else status=normal. In a given segment, outlier clones that yielded normalized log2-ratio values (Y) such that Y > L_N_ + 3 × SD (respectively Y < L_N_ - 3 × SD) are classified as gains (respectively losses). Amplicons were defined by median log ratios greater than 2.

**Gene expression: hybridization and pre-treatment**

***1. RNA extraction and Quality Control:*** tumor samples (10 to 50 mg) were powdered under liquid nitrogen. RNA were extracted using RNAeasy Mini Kits (Qiagen, Courtaboeuf, France). Aliquots of the RNA were analyzed by electrophoresis on a Bioanalyser 2100 (version A.02 S1292, Agilent Technologies, Waldbronn, Germany) and quantified using Nano Drop™ ND-1000 (Nyxor Biotech). Stringent criteria for RNA quality were applied to rule out degradation, especially a 28s/18s ratio above 1.8 for microarray.

***2. cRNA probe production and labeling:*** 3 mg of total RNA were amplified and labeled according to the manufacturer’s one-cycle target labeling protocol ([http://www.affymetrix.com](http://www.affymetrix.com/)). 10 mg of cRNA were used per hybridization (GeneChip Fluidics Station 400; Affymetrix, Santa Clara, CA). The labeled cRNAs were hybridized to HG-U133 plus 2.0 Affymetrix GeneChip arrays (Affymetrix, Santa Clara, CA). Chips were scanned with a Affymetrix GeneChip Scanner 3000 and subsequent images analyzed using GCOS 1.4 (Affymetrix).

***3. Affymetrix chip quality control:*** we used the R package affyQCReport to generate a QC report for all chips (CEL files) from the CIT discovery series. All chips that didn’t pass this QC filtering step were removed from further analysis.

***4. Normalization:*** raw feature data from Affymetrix HG-U133A Plus 2.0 GeneChip^TM^ microarrays are normalized using Robust Multi-array Average (RMA) method (R package affy) .

***5. Probe set filtering:*** probe sets corresponding to control genes and those for whom the 90e percentile of the log intensity did not reach log_2_(10) are masked, yielding a total of 52,188 probe sets available for further analyses.

**Tissue micro-array and immunohistochemistry**

Immunohistochemical analysis was performed on a Dako autostainer with antibodies specific for Estrogen receptor (clone 1D5, Dako Trappes France, 60 minutes, 1:25), Progesterone receptor (clone PgR636, Dako Trappes France, 60 minutes, 1:60), Her2 (polyclonal AO485, Dako®, Trappes France, 60 minutes, dilution 1:1500), MIb1 (clone Mib1, Dako Trappes France, 60 minutes, 1:100), p53 (clone DO7, Dako Trappes France), EGFR and CK5/6 (clone D5/16B4, Dako®, Trappes France, 60 minutes, dilution 1:50). Antigen retrieval was performed by heating 5μm tissue sections in 0.01M citrate buffer pH=6 in a pressure cooker for 20 minutes. A labeled streptavidin-biotin- peroxydase method (LSAB kit K5001, Dako®, France) was used for visualization of the immunoreactions with Diaminobenzidine as a chromogen.

**Agglomerative hierarchical clustering**

Agglomerative hierarchical clustering with Pearson correlation as a similarity measure and the Ward method to minimize the sum of the variances were used to produce compact spherical clusters (ref 2).

**Statistical tests**

***1. Differential expression:*** to identify genes differentially expressed between the sample subgroups, based on the RMA log_2_ single-intensity expression data, we used Welch’s T-tests (t.test function, R package stats).

***2. Differential genomic status:*** to identify clones / regions with differential genomic status, based on the GNL (Gain/Normal/Loss) copy number status, we used the chi-square test (or the equivalent Fisher’s-exact test when appropriate) (chisq.test and fisher.test functions, R package stats).

***3. Comparison to prognostic parameters:*** association of the G2I and the aCGH clusters classification with classical prognostic parameters was tested by applying the chi-square test (or the equivalent Fisher’s-exact test when appropriate).

***4. P-value adjustment:*** p-value adjustment for multiple-testing was performed using the p.adjust function from stats R package which estimates the FDR using the Benjamini and Hochberg (BH) method.

**Genomic instability index (G2I)**

The proposed score is based on two items: (i) the overall level of genomic alteration (noted
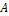
) and (ii) the number of altered genomic regions (noted
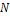
).

*Computing the overall level of genomic alteration (A):* The overall level of genomic alteration (A) is simply computed as the mean over each chromosome arm of the proportion of altered clones which is the number of altered clones (determined as ‘Gain’ or ‘Loss’) over the total number of clones for an arm:


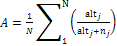
,

with
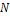
 the total number of arms,
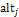
 the count of altered clones and
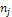
 the count of unaltered clones for the arm
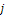
.

*Computing the number of altered regions (N):* Several approaches can be used to assess the number of altered genomic regions, either by using sliding-windows or by the determination of breakpoints. Here we have developed a novel approach based on local-score statistics. This statistic is generally used to delimit and count regions with a particular required property in a given signal. This has already been the subject of a important theoretical work (ref 3; ref 4; ref 5) and has been successfully applied to a wide range of bioinformatic applications (ref 6; ref 7; ref 8; ref 9). Here, local score statistics are used to delimit and count the number of altered genomic regions (deleted, gained or amplified arms) along all the genome. Conceptually, the size of a region can include from one clone to a whole chromosome arm. The local score region determination is applied to each CGH profile as described and implemented in Guedj et al. (ref 8) and Aschard et al. (ref 9), and the number of regions
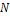
 is then retrieved for each sample.

*Computing the thresholds*
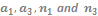
*:* By applying a set of appropriate thresholds on these two items, we are able to define three groups of genomic scores 1, 2 and 3, characterized by an increasing level of genomic perturbation. For a given sample
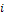
, let
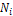
 and
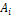
 equal the computed values
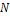
 and
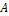
 respectively. Let
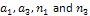
 the thresholds:


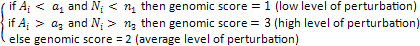


The estimates for
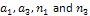
 result from an approach coupling a classification-based process with a numerical-constrained minimization. It is implemented in the g2i.learn R function (provided in the supplemental material), with parameter method set to “NM” (default value). Note that this approach uses random initialization steps, meaning that output can vary from one run to another.

We first compute the thresholds a3 and n3 (G3 identification), using the best solution over 500 iterations of the following procedure:

1. Random initialization of a3 and n3 (using ad hoc uniform distributions)
2. Look for a3 and n3 optimizing the function f(a3,n3) defined as the p-value of the logrank-test for differential metastatic relapse between G3 and non-G3 samples, G3 being defined based on a3 and n3. Here optimization is performed with the Nelder and Mead algorithm (ref 10; function optim from R package stats).

Once a3 and n3 have been obtained, G3 is defined and related samples are removed from the next step. We then compute the thresholds a1 and n1 (G1 identification), using the best solution over 500 iterations of the following procedure:

1. Random initialization of a1 and n1 (using ad hoc uniform distributions)
2. Look for a1 and n1 optimizing the function f(a1,n1) defined as the p-value of the logrank-test for differential metastatic relapse between G1 and non-G1 samples, G1 being defined based on a1 and n1. Here again optimization is performed with the Nelder and Mead algorithm. (ref 10). All previously identified G3 samples are not taken into account in this step.

**Validation of the Genomic Instability Index on an independent dataset**

Raw CGH BAC array data corresponding to 168 pT1T2pN0 invasive ductal carcinoma patients from an independent study (ref 11) were obtained from GEO website (http://www.ncbi.nlm.nih.gov/geo, dataset [GSE19159](http://www.ncbi.nlm.nih.gov/projects/geo/query/acc.cgi?acc=GSE19159)). These data were pre-treated (normalization + smoothing + Gain/Normal/Loss calling) as for our own series (see above chapter ‘CGHarray: hybridization and pre-treatment’). We then calculated the G2I score on this series using the formula described above [see chapter ‘Genomic instability index (G2I)’]. Finally we used Kaplan-Meier curves and a logrank test to assess differences between the predicted groups.

**Comparison of four prognostic molecular signatures on three independent datasets**

*Application of the G2I signature:* we used a nearest centroids predictor to classify tumors in two groups of good and poor prognosis; centroids were evaluated on training sets of tumors described later.

*Application of the Amsterdam signature:* the matching between the Amsterdam signature (ref 12) and an Affymetrix U133PPlus2 chip was based on the gene symbols, resulting in a total of 52 identified common genes over 70. We used a nearest centroids predictor to classify tumors in two groups of good and poor prognostic; centroids were evaluated on training sets of tumors described later.

*Application of the GGI:* we strictly followed the approach described by Sotiriou (ref 13) to attribute a GGI-score to each tumor and according to a determined threshold based on SBR, to divide a set of tumors into two groups of good and poor prognosis. The threshold used was evaluated on training sets of tumors described later.

*Application of the intrinsic signature:* the matching between the Norway/Stanford used by Sorlie and al. (ref 14) and an Affymetrix U133PPlus2 chip was based on the gene symbols, resulting in a total of 334 identified common genes. We used a nearest centroids predictor to classify tumors according to one of the five subtypes (basal, ERBB2+, luminal-B, luminal-A, normal-like) and then we made two groups: good prognostic by combining luminal-A and normal-like tumors, and poor prognostic by combining basal, ERBB2+ and luminal-B tumors. Centroids for each subtype were evaluated on the Norway/Stanford dataset.

## *Application to three independent datasets*

Application to RELB: the four prognostic signatures were applied to the RELB dataset. It should be noted that, as this dataset was used to construct the G2I molecular signature, performances of this signature on this dataset are positively biased. In order to attenuate this effect, we also positively biased the results of the Amsterdam and GGI signatures by using RELB as a training set to evaluate the centroids before applying the nearest centroids prediction on the same tumors. The centroids from the intrinsic signature are based on the independent Norway/Stanford dataset.

Application to Wang05: the four prognostic signatures were applied to Wang (ref 15). One third of the dataset (= 96 tumors including60 tumors of good prognostic and 36 tumors of poor prognostic) was used as a training set for the G2I, Amsterdam and GGI signatures. The centroids from the intrinsic signature are based on the independent Norway/Stanford dataset. Then the centroids were applied to the remaining independent set of tumous [= 190 tumors including 119 tumors of good prognosis with a median follow-up of 101 months (range: 20-171) and 71 tumors of poor prognostic) in order to compare the four signatures.

Application to Loi07: the four prognostic signatures were applied to Loi (ref 16). One third of the dataset (= 132 tumors divided into 85 tumors of good prognostic and 47 tumors of poor prognostic) was used as a training set for the G2I, Amsterdam and GGI signatures. The centroids from the intrinsic signature are based on the independent Norway/Stanford dataset. Then the centroids were applied to the remaining independent set of tumors (= 261 tumors including 169 tumors of good prognostic and 92 tumors of poor prognostic) in order to compare the four signatures. The median follow-up is 9.18 years (range: not done).

References

Ref 1: Irizarry R.A., Hobbs B., Collin F., Beazer-Barclay Y.D., Antonellis K.J., Scherf U., and Speed T.P. Exploration, normalization, and summaries of high density oligonucleotide array probe level data. Biostatistics 2003; 4 :249-2643.

Ref 2: Hartigan JA. Clustering Algorithm. New York: Wiley. 1975

Ref 3: Altschul S and Erickson B. Locally optimal subalignments similarity and its significance levels. Bulletin of Mathematical Biology. 1986; 48: 633-660.

Ref 4: Karlin S and Dembo A. Limit distributions of maximal segmental score among markov-dependant partial sums. Advances in Applied Probability. 1992; 24: 113-140.

Ref 5: Mercier S and Daudin J. Exact distribution for the local score of one i.i.d. random sequence. Journal of Computational Biology. 2001; 8: 373-380.

Ref 6: Karlin S and Brendel V. Chance and significance in protein and dna sequence analysis. Science. 1992; 257: 39-49.

Ref 7: Altschul S, Gish W, Miller W, Myers E and Lipman D. Basic local alignment search tool. Journal of Molecular Biology. 1990; 215: 403-410.

Ref 8: Guedj M, Robelin D, Hoebeke M, Lamarine M, Wojcik J and Nuel G. Detecting local high-scoring segments: a first-stage approach for genome-wide association studies. Stat. Appli. Genet. Mol. Bio. 2006; 5:22.

Ref 9: Aschard H, Guedj M and Demenais F. A multiple-marker two-step approach for genome-wide association studies. BMC Proceedings. 2007; 1:S134.

Ref 10: Nelder JA and Mead R. A simplex algorithm for function minimization. Computer Journal. 1965; 7: 308–313.

Ref 11 : Gravier E, Pierron G, Vincent-Salomon A, Gruel N, Raynal V, Savignoni A, De RY, Pierga JY, Lucchesi C, Reyal F, et al.. A prognostic DNA signature for T1T2 node-negative breast cancer patients. Genes Chromosomes Cancer 2010; 49:1125-1134.

Ref 12: Van't Veer LJ, Dai H, van de Vijver MJ, He YD, Hart AA, Mao M, Peterse HL, van der Kooy K, Marton MJ, Witteveen AT, et al.. Gene expression profiling predicts clinical outcome of breast cancer. Nature 2002; 415:530-536.

Ref 13: Sotiriou C, Wirapati P, Loi S, Harris A, Fox S, Smeds J, Nordgren H, Farmer P, Praz V, Haibe-Kains B, et al.. Gene expression profiling in breast cancer: understanding the molecular basis of histologic grade to improve prognosis. J Natl.Cancer Inst 2006; 98:262-272.

Ref 14: Sorlie T, Perou CM, Tibshirani R, Aas T, Geisler S, Johnsen H, Hastie T, Eisen MB, Van de Rijn M, Jeffrey SS, et al.. Gene expression patterns of breast carcinomas distinguish tumor subclasses with clinical implications. Proc Natl.Acad.Sci.U.S.A 2001; 98:10869-10874.

Ref 15: Wang Y, Klijn JG, Zhang Y, Sieuwerts AM, Look MP, Yang F, Talantov D, Timmermans M, Meijer-van Gelder ME, Yu J, et al. Gene-expression profiles to predict distant metastasis of lymph-node-negative primary breast cancer. Lancet 2005.;365:671-679.

Ref 16: Loi S, Haibe-Kains B, Desmedt C, Lallemand F, Tutt AM, Gillet C, Ellis P, Harris A, Bergh J, Foekens JA, et al. Definition of clinically distinct molecular subtypes in estrogen receptor-positive breast carcinomas through genomic grade. J Clin Oncol 2007; 25:1239-1246.
