# Supplementary material for: An array CGH based genomic instability index (G2I) is predictive of clinical outcome in breast cancer and reveals a subset of tumors without lymph node involvement but with poor prognosis
Source: BMC Med Genomics. 2012 Nov 27;5:54. doi: 10.1186/1755-8794-5-54 (PMC3558323; doi:10.1186/1755-8794-5-54)

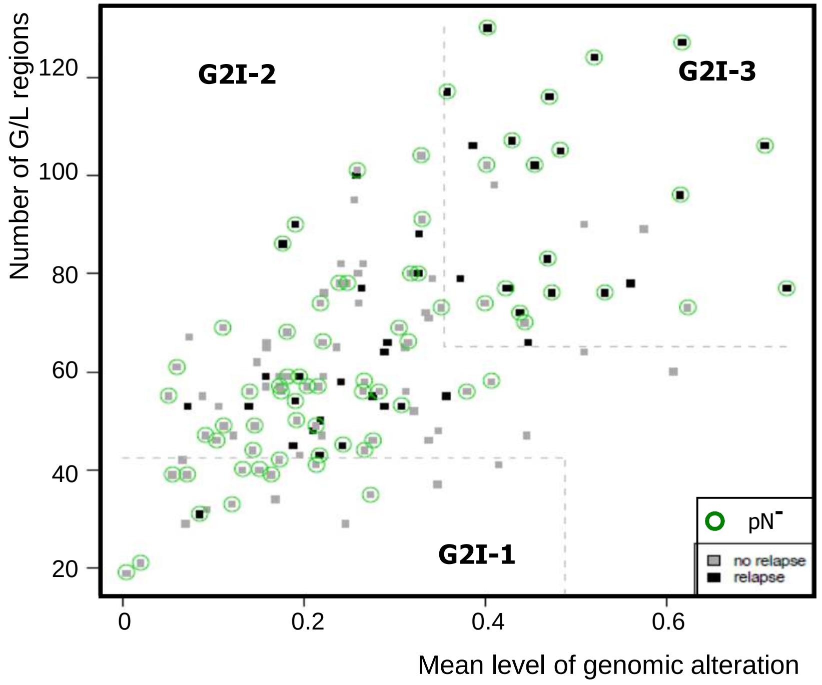

Supplementary figure 1: scatter plot of the 135 tumors according to the two items of the G2I showing the relatively high frequency of G2I-3 tumors without axillary lymph node involvement in comparison to the G2I-2 tumors (20/28 versus 43/88)

Supplementary figure 2: Frequency plot of copy number alterations in tumors belonging to the three classes of the G2I. Plot D shows the specificity of the numerous losses of material observed with a high frequency in G2I-3 tumors (in green in this plot).

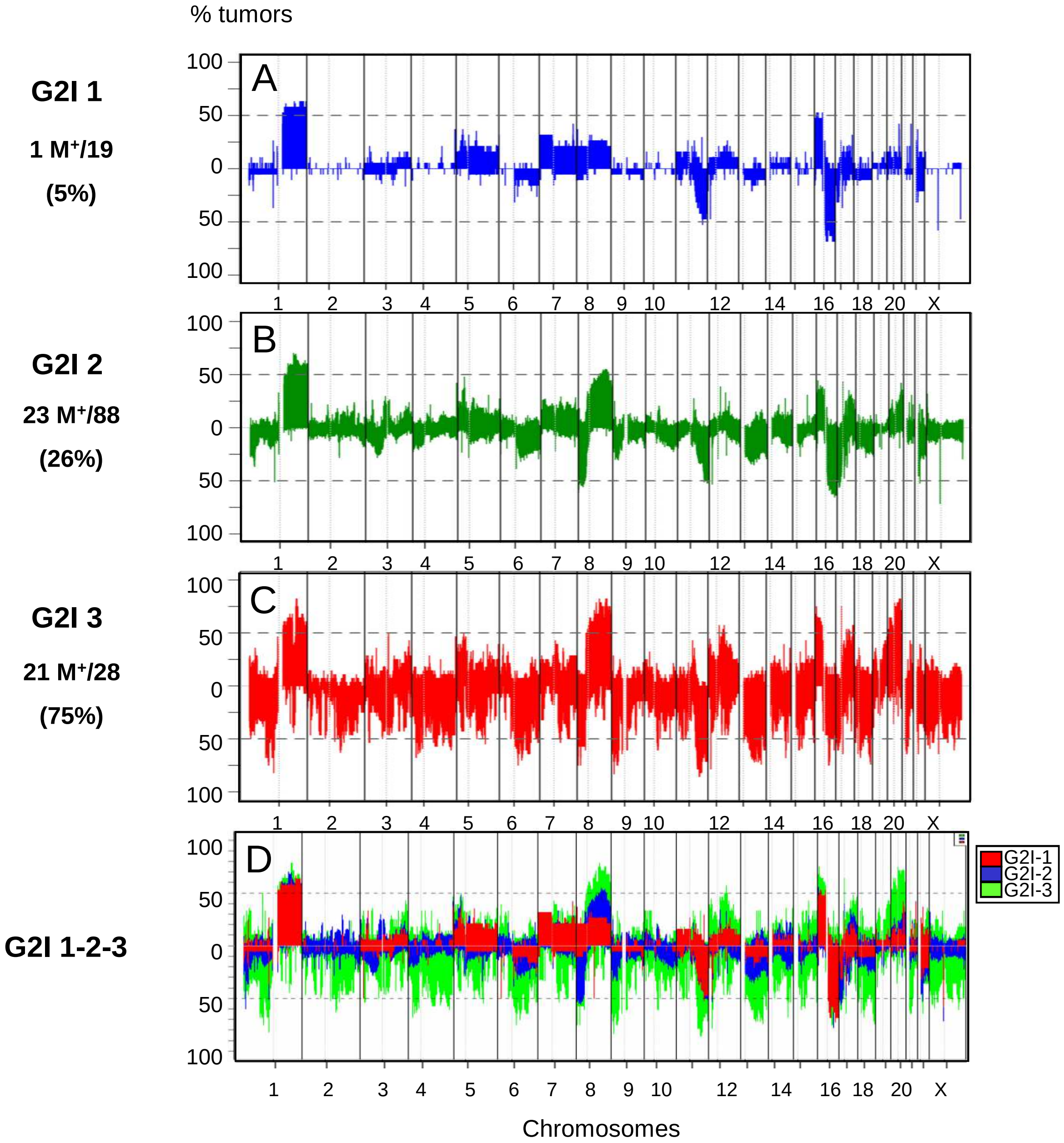

Supplementary figure 3: supervised hierarchical clustering showing the differential expression of 300 probe sets (listed in sup. table 9) between G2I-3 and G2I-2 tumors.

MR, black: metastatic relapse, white: no relapse; HR, black: steroid hormonal receptors positive, white: negative; her2, black: Immunohistochemistry Her2 3+, white: 1+ and 2+; p53, black: TP53 mutation or immunohistochemistry over expression, white: wild type; NPI, Nottingham Prognostic Index, red: NPI-1 or excellent, blue: NPI-2 or good, green: NPI-3 or moderate, orange: NPI-4 or poor prognostic; G2I: Genomic instability index, red: G2I-1, blue: G2I-2, green: G2I-3 class of tumors.

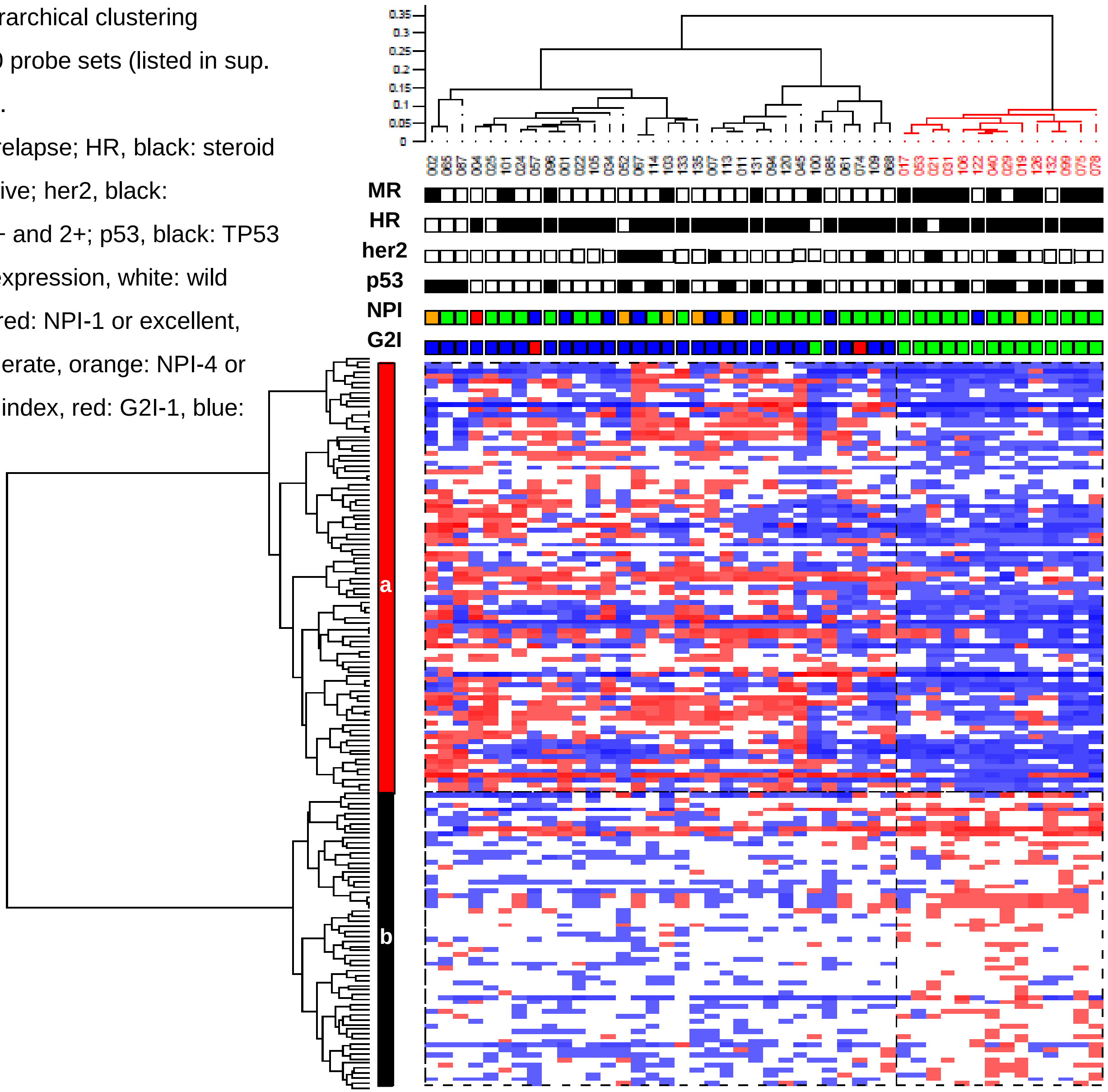

Supplement: Additional file 4 — Supplementary figures. File containing three supplementary figures; scatter plot of the 135 tumors according to the G2I and axillary lymph node involvement; frequency plot of genomic copy number variation in tumors belonging to the 3 classes of G2I; Hierarchical clustering showing the differential expression of 300 probe sets between G2I-3 and G2I-2 tumors. (PDF 279 kb) [file 1755-8794-5-54-S4.pdf]
